# Tachykinins: Neuropeptides That Are Ancient, Diverse, Widespread and Functionally Pleiotropic

**DOI:** 10.3389/fnins.2019.01262

**Published:** 2019-11-20

**Authors:** Dick R. Nässel, Meet Zandawala, Tsuyoshi Kawada, Honoo Satake

**Affiliations:** ^1^Department of Zoology, Stockholm University, Stockholm, Sweden; ^2^Department of Neuroscience, Brown University, Providence, RI, United States; ^3^Bioorganic Research Institute, Suntory Foundation for Life Sciences, Kyoto, Japan

**Keywords:** substance P, neurokinin, neurokinin receptor, natalisin, G protein-coupled receptor, co-transmission, neuropeptide evolution, tachykinin-related peptide

## Abstract

Tachykinins (TKs) are ancient neuropeptides present throughout the bilaterians and are, with some exceptions, characterized by a conserved FX_1_GX_2_Ramide carboxy terminus among protostomes and FXGLMamide in deuterostomes. The best-known TK is the vertebrate substance P, which in mammals, together with other TKs, has been implicated in health and disease with important roles in pain, inflammation, cancer, depressive disorder, immune system, gut function, hematopoiesis, sensory processing, and hormone regulation. The invertebrate TKs are also known to have multiple functions in the central nervous system and intestine and these have been investigated in more detail in the fly *Drosophila* and some other arthropods. Here, we review the protostome and deuterostome organization and evolution of TK precursors, peptides and their receptors, as well as their functions, which appear to be partly conserved across Bilateria. We also outline the distribution of TKs in the brains of representative organisms. In *Drosophila*, recent studies have revealed roles of TKs in early olfactory processing, neuromodulation in circuits controlling locomotion and food search, nociception, aggression, metabolic stress, and hormone release. TK signaling also regulates lipid metabolism in the *Drosophila* intestine. In crustaceans, TK is an important neuromodulator in rhythm-generating motor circuits in the stomatogastric nervous system and a presynaptic modulator of photoreceptor cells. Several additional functional roles of invertebrate TKs can be inferred from their distribution in various brain circuits. In addition, there are a few interesting cases where invertebrate TKs are injected into prey animals as vasodilators from salivary glands or paralyzing agents from venom glands. In these cases, the peptides are produced in the glands of the predator with sequences mimicking the prey TKs. Lastly, the TK-signaling system appears to have duplicated in Panarthropoda (comprising arthropods, onychophores, and tardigrades) to give rise to a novel type of peptides, natalisins, with a distinct receptor. The distribution and functions of natalisins are distinct from the TKs. In general, it appears that TKs are widely distributed and act in circuits at short range as neuromodulators or cotransmitters.

## Introduction

Substance P, the prototypic tachykinin (TK), was the first neuropeptide ever to be isolated from brain tissue already in 1931 (Von Euler and Gaddum, 1931). For a long time it was the sole brain neuropeptide known, and was joined only in the 1950s by the pituitary peptides, oxytocin and vasopressin (Turner et al., 1951; Du Vigneaud et al., 1953; Du Vigneaud, 1955). Today, the number of neuropeptides identified in the animal kingdom is huge and hard to overview [see (Jekely, 2013; Mirabeau and Joly, 2013; Nässel and Zandawala, 2019)]. Also, the number of TK family members has grown immensely over the years, and we know now that there often are several structural and functional representatives in each species.

Already from the outset it was recognized that substance P is produced both in the brain and the intestine (Von Euler and Gaddum, 1931; Hökfelt et al., 2001). Today, it is clear that TKs are utilized by neurons in the CNS, by neurons and enteroendocrine cells associated with the intestine (Otsuka and Yoshioka, 1993; Nässel, 1999; Hökfelt et al., 2001; Satake et al., 2013; Steinhoff et al., 2014), as well as by other cells in mammals such as hematopoietic cells (Zhang et al., 2000; Morteau et al., 2001), endothelial cells, Leydig cells and immune cells [see (Almeida et al., 2004)]. Thus, they are widespread and pleiotropic, and are not only neuropeptides, but also produced by other cell types.

Although the first TK was identified in 1931, it was not until 1971 that substance P was purified and sequenced from 20 kg bovine hypothalamus (Chang et al., 1971), and subsequently synthesized (Tregear et al., 1971). This enabled production of antisera and their application in radioimmunoassay (Powell et al., 1973) and immunocytochemistry (Hökfelt et al., 1975) to localize substance P and demonstrate its release. Thereafter, important experimental work ensued, including development of TK agonists and antagonists [see (Otsuka and Yoshioka, 1993; Hökfelt et al., 2001)], identification of TK receptors [see (Masu et al., 1987; Nakanishi, 1991)], developing genetic approaches and discovering important roles in health and disease [see (Otsuka and Yoshioka, 1993; Hökfelt et al., 2001; Onaga, 2014; Steinhoff et al., 2014)]. The discovery of the roles of substance P and other TKs in pain, inflammation, cancer, depressive disorder, immune function, gut function, hematopoiesis, sensory processing and hormone regulation [see (Hökfelt et al., 2001; Onaga, 2014; Steinhoff et al., 2014; Zieglgänsberger, 2019)] has lead to extensive research into the pharmacology and molecular biology of this signaling system as a therapeutic target [see (Steinhoff et al., 2014)], resulting in a huge number of publications annually. However, it is hard to find recent comprehensive reviews on TKs that cover distribution and functions.

**FIGURE 1:**
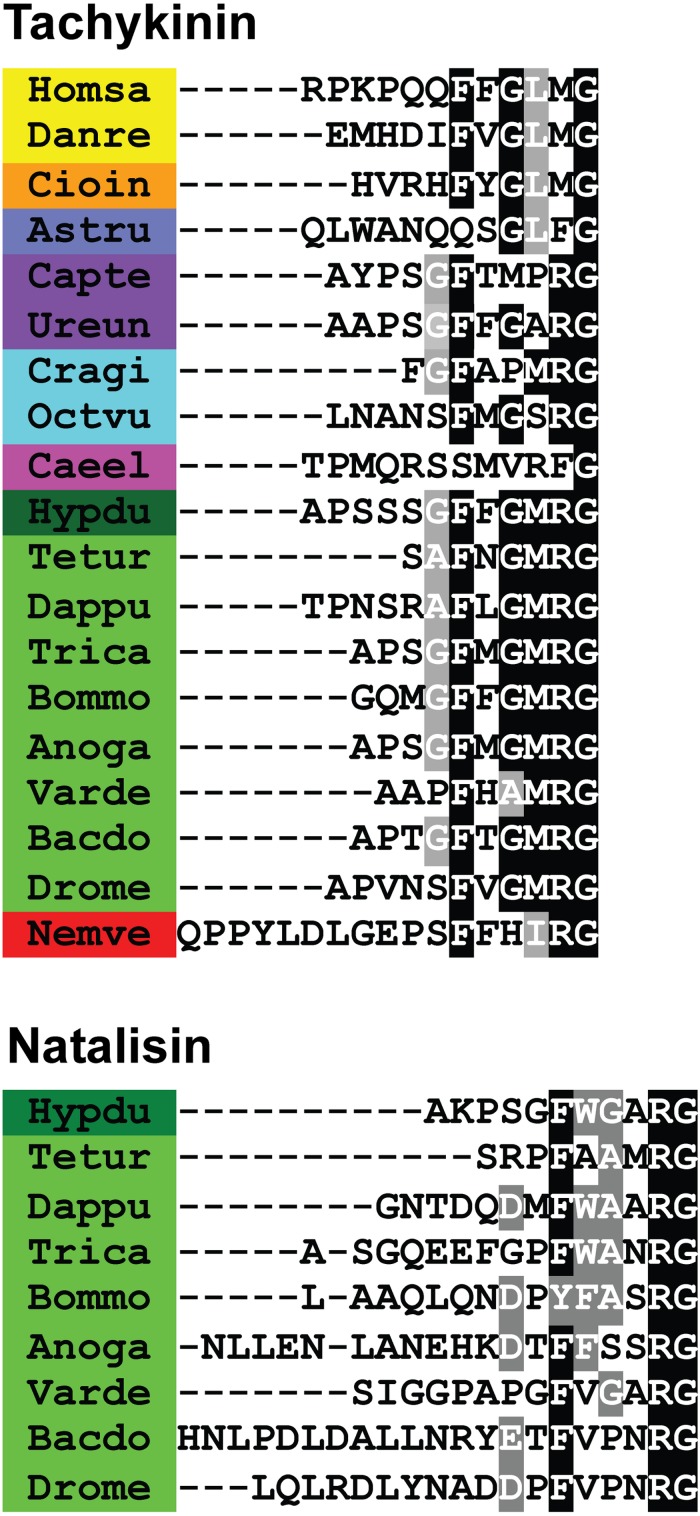
Sequence alignments of **(A)** tachykinin and **(B)** natalisin peptides from select species. Note that C-terminal amidation is not shown; it is represented by the amidation signal G. Conserved residues are highlighted in black (identical) or gray (similar). Species belonging to the same phyla have been highlighted with the same color. Species names are as follows: Homsa (*Homo sapiens*), Danre (*Danio rerio*), Cioin (*Ciona intestinalis*), Astru (*Asterias rubens*), Capte (*Capitella teleta*), Ureun (*Urechis unicinctus*), Cragi (*Crassostrea gigas*), Octvu (*Octopus vulgaris*), Caeel (*Caenorhabditis elegans*), Hypdu (*Hypsibius dujardini*), Tetur (*Tetranychus urticae*), Dappu (*Daphnia pulex*), Trica (*Tribolium castaneum*), Bommo (*Bombyx mori*), Anoga (*Anopheles gambiae*), Varde (*Varroa destructor*), Bacdo (*Bactrocera dorsalis*), Drome (*Drosophila melanogaster*) and Nemve (*Nematostella vectensis*). Note that the *A. rubens, C. elegans* and *N. vectensis* peptides are unlikely to be TKs as they deviate substantially from the canonical TK sequences (see also text).

Tachykinins have also been explored outside mammals and other vertebrates. The first TK to be identified in an invertebrate was eledoisin, isolated from salivary glands of the cephalopod *Eledone moschata* (Erspamer and Anastasi, 1962). Eledoisin was actually the first TK to be sequenced, but since the sequence of substance P was not yet known, the structural relationship was realized only later. The authors, however, recognized that the action of eledoisin on mammalian smooth muscle is similar to that of substance P (Erspamer and Anastasi, 1962; Erspamer and Erspamer, 1962). Many years later, four TKs were isolated from the brain and retrocerebral glands of the locust *Locusta migratoria* (Schoofs et al., 1990a, b). Today, multiple TKs (more than 350 sequences) have been identified from over 50 insect species [see the DINeR database^1^ (Yeoh et al., 2017)], and numerous ones from other invertebrates and protochordates [see e.g., Kawada et al. (2010), Veenstra (2010, 2011, 2016), Conzelmann et al. (2013), Palamiuc et al. (2017), Zandawala et al. (2017), Dubos et al. (2018), Koziol (2018) and Figure 1 and Supplementary Table S1]. Also in invertebrates, the common TKs are produced by neurons of the CNS and by endocrine cells of the intestine, but the presence of invertebrate TKs in other cell types has not been reported thus far. Functional analysis has revealed that invertebrate TKs are also pleiotropic. Moreover, recent genetic work in *Drosophila*, suggests that many TK functions are conserved over evolution.

In this review, we first comment on TK terminology in invertebrates since at present it may seem somewhat complex and confusing. Furthermore, we discuss the evolution of genes encoding TK precursors and receptors as well as outline TK signaling systems in various phyla across the animal kingdom. Next, we discuss the distribution of TKs and functions of TK signaling systems; here, we are more comprehensive in dealing with invertebrates since vertebrate TK literature is very extensive. Furthermore, we highlight the functions of TK-signaling that are conserved across different animal phyla. Of note, TKs generally appear to signal over a relatively short range within defined neuronal circuits as neuromodulators or cotransmitters. Only a few examples of intestinal TKs acting as local circulating hormones are available. We also discuss a sister group of the TKs, the natalisins, that seems to have arisen by a gene duplication in the Panarthropoda (comprising arthropods, onychophores, and tardigrades) lineage and appears restricted to this group. The natalisins and their receptors constitute a distinct signaling system that has not been investigated in detail thus far.

## Structure of Tachykinin Peptides and Organization of Genes Encoding Their Precursors

We start this section with a commentary on TK terminology and continue with describing TK precursors, peptides and receptors in mammals where knowledge is the largest and then move on with other vertebrates and last invertebrates.

### A Note on Major Types of Tachykinins and Terminology

Substance P (RPKPQQFFGLMamide), and other mammalian TKs, are characterized by an FXGLMamide carboxy terminus, and these peptides act on either of three TK receptors (GPCRs; NK1R – NK3R) (Otsuka and Yoshioka, 1993; Onaga, 2014; Steinhoff et al., 2014). The first invertebrate neuropeptides referred to as TKs (locustatachykinin I-IV; LomTK I-IV), were isolated from the brain and retrocerebral complex of locusts, and have a different carboxy terminus, FX_1_GX_2_Ramide (Schoofs et al., 1990a, b). This sequence is shared by many other invertebrate TKs and only one type of insect TK receptor is known so far (Nässel, 1999; Van Loy et al., 2010; Satake et al., 2013). The structural difference in the active core of the two groups of TK peptides renders the FX_1_GX_2_Ramides inactive on the vertebrate-type TK receptors and conversely, vertebrate TKs do not activate invertebrate receptors (Satake et al., 2013). Thus, these authors suggested that the FX_1_GX_2_Ramides should be designated tachykinin-related peptides (TKRPs) to distinguish them from vertebrate TKs with FXGLMamide. In the literature, the individual TKs and TKRPs have been given many different names. In invertebrates, these commonly include a prefix indicating the species of origin (e.g., LomTK in *Locusta migratoria*) and then numbers if multiple peptide paracopies (isoforms) exist on the same precursor (LomTK-I, LomTK-II etc).

**FIGURE 2:**
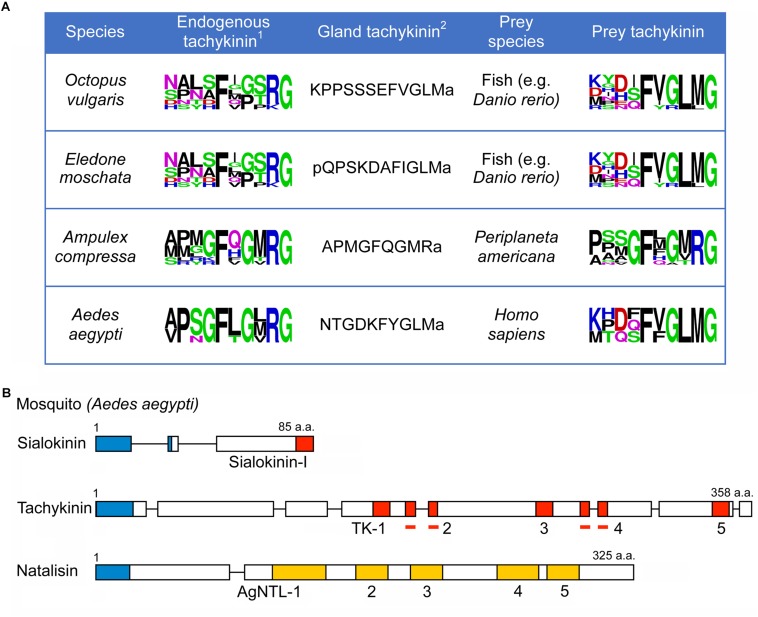
Tachykinins produced by glands utilized on prey animals. **(A)** Sequences of TKs produced by salivary- or venom glands of cephalopods (*O. vulgaris* and *E. moschata*) a wasp (*A. compressa*) and a mosquito (*A. aegypti*) compared to the endogenous TKs and TKs of prey animals (shown as SeqLogos). We use the zebrafish *D. rerio* as an example for the possible cephalopod prey^1^. The endogenous TK of *E. moschata* is not known; that of *O. vulgaris* is shown instead^2^. Only one sequence is shown. **(B)** Scheme of organization of tachykinin precursors and a natalisin precursor of *A. aegypti*. The salivary gland TK is designated sialokinin-I. Red boxes represent tachykinins, and yellow boxes indicate natalisins. In addition, signal peptides are indicated by blue boxes. Bars indicate tachykinins (TK-2 and TK-4) that are part of multiple exons.

To further complicate the terminology of TKs, there are peptides with an FXGLMamide carboxy terminus produced by salivary glands of mosquitos, sialokinins (Champagne and Ribeiro, 1994) and in cephalopods, eledoisin and octopus-tachykinin (Erspamer and Anastasi, 1962; Kanda et al., 2003; Satake et al., 2003) (Figure 2 and Supplementary Table S2). These TKs are delivered to prey and meant to act on exogenous receptors, not within the “sender animal” (predator). The peptides of this kind were referred to as invertebrate TKs (Inv-TKs) (Satake et al., 2003), to distinguish them from TKRPs. Similarly, exocrine glands in amphibian skin produce FXGLMamide-type TKs that have been given different exotic names [see (Lazarus and Attila, 1993)]. A recent finding adds to the TK complexity; in the parasitoid Jewel wasp (*Nasonia vitripennis*), the toxin glands produce a precursor encoding multiple FQGMRamide containing peptides (Arvidson et al., 2019). The wasp injects the toxin that contains FQGMRamide peptide and other components into the cockroach brain to paralyze the host by acting on the cockroach TK receptor in circuits of the central complex. In summary, TKs for exogenous use are produced to act on receptors of target animals and the salivary gland ones deviate structurally from native TKs. We will discuss these in more detail later. In the present review, we use the names originally given to the different TKs when relevant (see Supplementary Tables 1, 2), but for the sake of simplicity we will henceforth use the term TKs for all FXGLMamide and FX_1_GX_2_Ramides when we discuss the peptides in general.

A related, but distinct, invertebrate peptide signaling system is constituted by the natalisins (NTL) and their receptors (Jiang et al., 2013). These will be discussed separately. Also, note that especially in early papers (but also some more recent ones) a family of neuropeptides designated leucokinins (LKs) has been considered related to TKs [see e.g., Holman et al. (1986), Nässel and Lundquist (1991), Al-Anzi et al. (2010)]. The LKs have an FXSWGamide carboxy terminus, and analysis based on precursor structure (and receptors) show that they are not homologous to TKs (Jekely, 2013; Mirabeau and Joly, 2013).

### TKs and Their Receptors in Mammals

**FIGURE 3:**
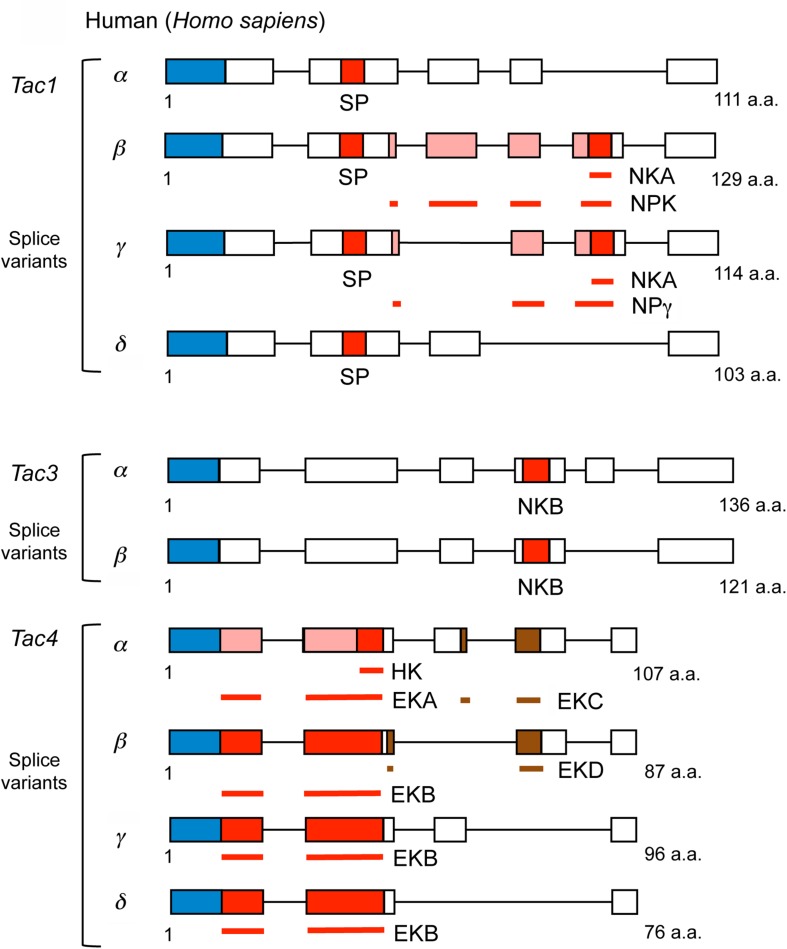
Scheme of human tachykinin precursors (*Tac1, Tac3*, and *Tac4* and their splice variants). Boxes and lines show exons and introns, respectively. NPK and NPg contain NKA sequences indicated by the red box at C-terminus, the N-termini of NPK and NPγ are represented by pink boxes, and brown boxes represent EKC or EKD that are not tachykinins. In addition, signal peptides are indicated by blue boxes. Bars indicate tachykinin or natalisin peptides that are part of multiple exons: NKA, NPK, NPγ, EKA, EKB, EKC, EKD, respectively. The Tac4 peptides are designated endokinins A - D (EKA – EKD); hemokinin-1 (HK) represents the C-terminal portion of EKA. The N-terminus of EKA is shown as pink boxes. Primary sequence data from Steinhoff et al. (2014).

**FIGURE 4:**
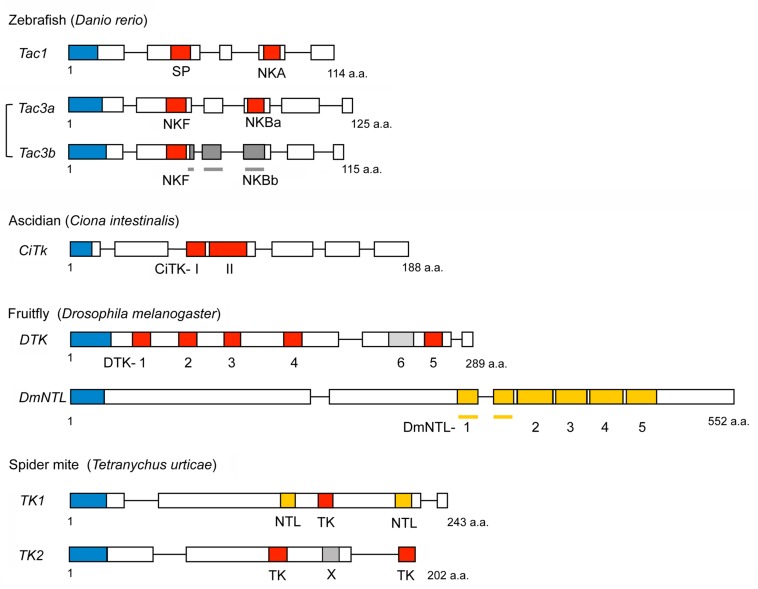
Schemes of representative non-mammalian tachykinin precursors and arthropod natalisin (NTL) precursors. Boxes and lines show exons and introns, respectively. Red boxes represent tachykinins, and yellow boxes indicate natalisins (signal peptides are indicated by blue boxes). Peptides with deviating sequences are shown in different shades of gray. NKBb is not a tachykinin. DTK6 also differs from *Drosophila* TKs (has a FVAVRa C-terminus) and has not been confirmed by mass spectrometry. Note that the spider mite *TK1* precursor has both a TK and two NTLs; also note that the NTL sequences are only predictions (Veenstra et al., 2012). The spider mite peptide X has the sequence ARPFAAMLamide distinct from both TKs and NTLs. Primary sequence data from Veenstra et al. (2012).

**FIGURE 5:**
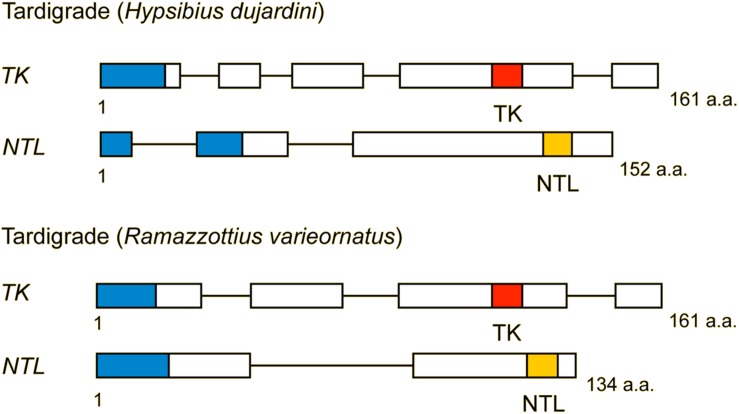
Structure of TK and natalisin (NTL) precursors from two tardigrades (*Hypsibius dujardini* and *Ramazottius varieornatus*). Red boxes represent tachykinins, and yellow boxes indicate natalisins (signal peptides are indicated by blue boxes). These precursors each contain one TK and one predicted NTL. Note that the NTL sequences are predictions and have not been confirmed by mass spectrometry. Compiled from sequence data in Koziol (2018).

**FIGURE 6:**
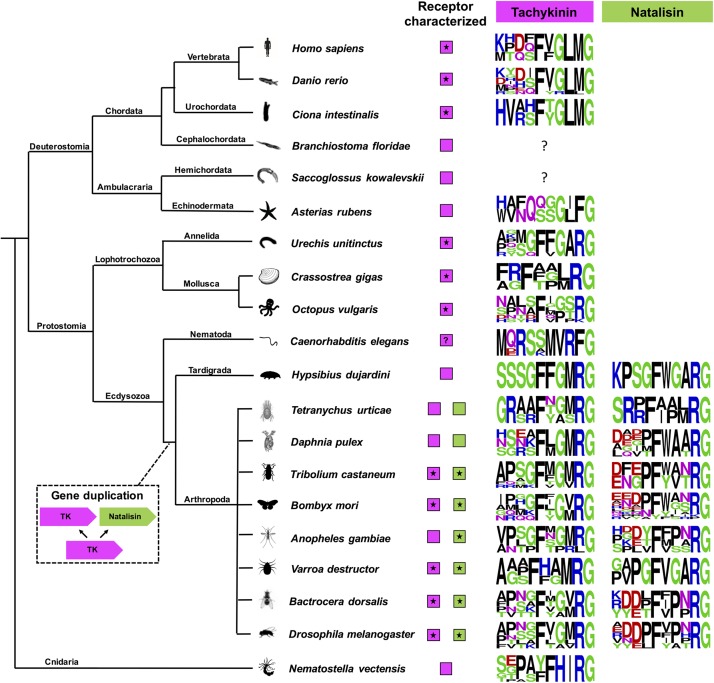
A cladogram showing the occurrence of tachykinin and natalisin signaling systems in Bilateria and Cnidaria. Sequence logos of the peptides have also been provided. These were constructed using the final 10 amino acids at the C-terminus. Note that C-terminal amidation is not shown; it is represented by the amidation signal G. Species in which the receptors have been functionally characterized are indicated by asterisk. Note that the natalisin signaling system appears to have arisen in the lineage leading up to tardigrades and arthropods. The tachykinin-like peptide sequences in *Asterias rubens, Caenorhabditis elegans*, and *Nematostella vectensis* diverge from the canonical TK sequences in other phyla and should probably not be classified as TKs (see text). In *C. elegans* a TK receptor has not been functionally characterized, although an FLP-7/NPR-22 signaling system has been proposed (Palamiuc et al., 2017), but shown to represent a luqin/RYamide signaling system (Ohno et al., 2017). The peptide encoded by the TK2 precursor in *Hypsibius dujardini* looks more similar to arthropod natalisins. Precursors encoding TK-like peptides have not yet been identified in *Branchiostoma floridae* and *Saccoglossus kowalevskii*. However, TK-like receptors are present in all the animals presented here.

In mammals, including humans, there are three genes encoding precursors of tachykinins: preprotachykinin A (PPTA), preprotachykinin B (PPTB) and preprotachykinin C (PPTC), also known as Tac1, Tac3, and Tac4, respectively [see (Onaga, 2014; Steinhoff et al., 2014)]. These genes arose through two rounds of genome duplications in the vertebrate lineage followed by subsequent gene losses and diversification (Elphick et al., 2018). The Tac1 precursor gives rise to Substance P (SP), Neurokinin A (NKA), Neuropeptide K (NPK), and neuropeptide γ (NPγ), Tac3 to Neurokinin B (NKB), and Tac4 to Hemokinin (HK), Endokinin-A (EKA) and Endokinin-B (EKB). Thus, there are nine different TKs in mice, rats and humans. The sequences of the Tac1 and Tac3 derived TKs are conserved in humans, mouse and rat, whereas the ones encoded on Tac4 differ between species (Steinhoff et al., 2014). The Tac4 encodes another two endokinins (EKC and EKD) that are not TKs (Steinhoff et al., 2014). The organization of mammalian TK precursors is shown in Figure 3 and their sequences in Table 1. For comparison, TK precursors in other representative animals are shown in Figures 4, 5, and a cladogram with TK signaling components found in Figure 6. It is also worth noting that the Tac1 and Tac4 genes each give rise to 4 splice variants, α, β, γ, and δ (Onaga, 2014; Steinhoff et al., 2014). The TKs have differential affinity for three different TK receptors, NK1R - NK3R (or TAC1R - TAC3R) (Nakanishi, 1991; Onaga, 2014; Steinhoff et al., 2014) as shown in Table 1; the ligand selectivity is as follows: SP > NKA > NKB for NK1R, NKA > NKB > SP for NK2R, and NKB > NKA > SP for NK3R (Satake et al., 2013; Steinhoff et al., 2014). HK and EKs exhibit the highest affinity to NK1R (Satake et al., 2013; Steinhoff et al., 2014). These are G-protein-coupled receptors (GPCRs) of the rhodopsin family (also known as family A GPCRs). The NK2R (neuropeptide K receptor) is of historical interest since it was the first neuropeptide receptor to be cloned (Masu et al., 1987). Signaling through the NK receptors is diverse and complex. For example, the ligand-activated NK1R initiates G-protein mediated signaling that can lead to (1) activation of phospholipase C (PLC), which results in formation of inositol trisphosphate (IP3) and diacylglycerol (DAG), mobilization of intracellular stores of Ca^2+^, and activation of PKC; (2) activation of adenylyl cyclase (AC), resulting in formation of cAMP, and activation of PKA; or (3) activation of phospholipase A2 and production of arachidonic acid (Steinhoff et al., 2014).

**TABLE 1:** Human tachykinins.^1^

**Tachykinin**	**Gene**^2^	**Receptor**	**Amino acid sequence**
Substance P	Tac1^3^	NK1R	RPKPQQFFGLMa
Neurokinin A	Tac1	NK2R	HKTDSFVGLMa
Neuropeptide K	Tac1	NK2R	DADSSIEKQVALLKALYGH GQISHKRHKTDSFVGLMa
Neuropeptide γ	Tac1	NK2R	DAGHGQISHKR HKTDSFVGLMa
Neurokinin B	Tac3	NK3R	DMHDFFVGLMa
Hemokinin-1	Tac4^4^	NK1R	TGKASQFFGLMa
Endokinin-A	Tac4	NK1R	DGGEEQTLSTEAETWVIVALEEGAG PSIQLQLQEVKTGKASQFFGLMa
Endokinin-B	Tac4	NK1R	DGGEEQTLSTEAETWEGAQ LQLQEVKTGKASQFFGLMa

*^1^Sequence and receptor data from Onaga (2014); Steinhoff et al. (2014). The sequences of the Tac1 and Tac3 derived peptides are conserved in humans, mouse and rat. ^2^These are also known as Ppt-a, Ppt-b and Ppt-c. ^3^Tac1 gene gives rise to four splice forms: a, b, g, and d, see Figure 3. ^4^Tac4 gene also gives rise to four splice forms, a, b, g, and d (see Figure 3), and two additional peptides (Endokinin-C and D) that are not TKs.*

In mammals, TKs play roles as neuromodulators/cotransmitters in central brain circuits, as well as in pain, stress, anxiety, depressive disorder, aggression, memory formation, inflammation, cancer, immune function, gut function, hematopoiesis, sensory processing, reproduction and cytokine and hormone regulation [see (Otsuka and Yoshioka, 1993; Felipe et al., 1998; Hökfelt et al., 2001; Holsboer, 2009; Onaga, 2014; Steinhoff et al., 2014; Lénárd et al., 2018; Zieglgänsberger, 2019)].

Substance P was mapped to neurons in the rat nervous system early on (Hökfelt et al., 1975, 1977; Ljungdahl et al., 1978). Now, we know that the distribution of SP and other TKs, as well as their receptors, is widespread and plastic. Receptor expression is regulated by various transcription factors under different physiological states. For instance, the receptors can be upregulated during inflammation via the transcription factor NF-κB (Onaga, 2014; Steinhoff et al., 2014). SP and NKA and their receptors are not only widely distributed throughout the central and peripheral nervous system, but also in many other tissues including dermal tissue, gastrointestinal tract, as well as the respiratory, urogenital and immune systems (Hökfelt et al., 2001; Onaga, 2014; Steinhoff et al., 2014). Whereas SP and NKA are expressed throughout the brain in mammals, NKB is found mainly in the hypothalamus and spinal cord. Furthermore, SP is present in brain circuits that are involved in the processing of anxiety, such as the amygdala, septum, mid-brain, periaqueductal gray, hippocampus, and hypothalamus (Holsboer, 2009).

As another example, in zebrafish, *Tac1* transcript has been mapped to neurons in the olfactory bulb, telencephalon, preoptic region, hypothalamus, mesencephalon, and rhombencephalon, whereas *Tac3a* was observed in the preoptic region, habenula and hypothalamus and *Tac3b* predominantly expressed in the dorsal mesencephalon (Ogawa et al., 2012). Additional details of SP and NKA distribution are beyond the scope of this review.

A few further examples of TK signaling are given here that are of interest for the discussion of TK functions in invertebrates in later sections. Certain taste cells express NK1R, and SP appears to regulate responses not only to toxins, but also to tastants: spicy foods stimulate SP release to enhance umami taste reception (Onaga, 2014). In the dorsal horn of the spinal cord, SP modulates nociceptive signals relayed to the brain, but also in pain-processing areas of the brain cortex (Felipe et al., 1998; Zieglgänsberger, 2019). NKB regulates hormone (e.g., gonadotropin-releasing hormone, GnRH) release in the hypothalamus (Steinhoff et al., 2014). NKRs are densely distributed in the rat olfactory bulb and it was shown that SP acts to depress neuronal activity in glomerular neurons, by triggering release of GABA (Olpe et al., 1987), similar to TKs and GABA in the antennal lobe of *Drosophila* (Ignell et al., 2009; Ko et al., 2015). The intestine is supplied by processes from TK-expressing neurons in dorsal root ganglia, or from local neurons (Hökfelt et al., 2001). NKRs are widely expressed in the intestine (in a cell-specific manner) by enteric neurons, intestinal muscle, epithelium, vasculature as well as immune system. TK signaling in the gut thus influences motility, electrolyte and fluid secretion, as well as vascular and immune functions (Hökfelt et al., 2001).

Recently, all NKRs were also shown to be expressed in genital organs and cells including the testis, sperm, ovary, granulosa cells, cumulus cells, and the uterus, and shown to be involved in sperm motility and reproduction (Pinto et al., 2010, 2015; García-Ortega et al., 2014; Candenas et al., 2018; Blasco et al., 2019).

Substance P is also known to activate three Mas-related GPCRs (Mrgprs), a promiscuous group of receptors underlying itch: human MRGPRX2, mouse MrgprA1, and mouse MrgprB2 (Bader et al., 2014). It is clear that the Mrgpr group is entirely separated from the TKR group (Bader et al., 2014). Interaction of SP with the Mrgprs induces elevation of intercellular Ca^2+^ (Azimi et al., 2016). The EC_50_ value of SP for human MRGPRX2 is approximately 150 nM, while EC_50_ values of SP for mouse MrgprA1 and MrgprB2 are about 5 μM and 50 μM, respectively (Azimi et al., 2016). Interestingly, analyses using *NK1R* knockout mice suggested that SP induces itch via Mrgprs rather than the NK1R (Azimi et al., 2017). Mrgprs are specific to mammals, suggesting that new SP-recognizing receptors arose during mammalian evolution.

### TKs and Receptors in Protochordates and Non-mammalian Vertebrates

Genomes of non-mammalian vertebrates possess receptors that are homologous to NK1R – NK3R (Biran et al., 2012; Satake et al., 2013). Likewise, several genes encoding TK homologs are present in non-mammalian vertebrate species. *Tac1* and *Tac3* have also been identified from zebrafish, and the *Tac3* prototype gene appears to have duplicated to give rise to *Tac3a* and *Tac3b* (Biran et al., 2012) as shown in Figure 4. In addition, two *Tac3* have also been characterized from an eel, *Anguilla anguilla*, that is a basal vertebrate (Campo et al., 2018). Due to teleost-specific whole genome duplication multiple *Tac3* genes were generated in teleost fish (Moriyama and Koshiba-Takeuchi, 2018). *Tac3a* of zebrafish encodes not only an NKB (NKBa), but also an NKF that is a piscine-specific TK (Biran et al., 2012). *Tac3b* of zebrafish encodes both an NKB (NKBb) and an NKF, although NKBb contains an FVGLLamide sequence at the C-terminus that differs from TK consensus sequence FXGLMamide (Biran et al., 2012). Gene duplication of zebrafish TK receptor genes has also occurred, resulting in two *NK1R* genes (*Tacr1a, Tacr1b*) and three *NK3R* genes (*Tacr3a, Tacr3b, and Tacr3c*) (Biran et al., 2012). Both TACR3a and TACR3b are efficiently activated by zebrafish NKBa and NKF, and their interaction induces both elevation of intercellular Ca^2+^ and production of cAMP (Biran et al., 2012). EC_50_ values of NKBb for TACR3a and TACR3b are 50–100 fold higher than that of NKBa for TACR3a and TACR3b (Biran et al., 2012). It is not clear whether homologs of HK and EKs are present in non-mammalian vertebrates, although it was proposed that *Tac4* is present in fish, including zebrafish, *Danio rerio* (Biran et al., 2012).

More than 20 TKs have been identified from skin secretion of frogs including, *Odorrana grahami*, *Rana chensinensis*, *Theloderma kwangsiensis, Kassina senegalensis*, and *Physalaemus fuscumaculatus* (Supplementary Table S3). These skin TKs possess the characteristic TK consensus sequence FXGLMamide (Bertaccini et al., 1965; Anastasi et al., 1977; Li et al., 2006; Wu et al., 2013; Zhang et al., 2013). The frog skin TKs are likely to act as exogenous factors, for instance as antimicrobial substances, rather than endogenous neuropeptides or hormones. An endogenous tachykinin has also been isolated from the brain of the frog, *Rana ridibunda* with a sequence homologous to that of NKB (O’Harte et al., 1991). Moreover, *Tac1* and *Tac3* of the frog, *Xenopus tropicalis*, have been predicted (registered in NCBI databases). The *X. tropicalis Tac1* encodes an SP-like and an NKA-like peptide, while *Tac3* gene encodes an NKB-like and an NKF-like peptide. Interestingly, the sequence of the *T. kwangsiensis* skin TK is identical to that of the *X. tropicalis* SP-like peptide, except for two amino acid residues. The sequence of the *K. senegalensis* skin TK is also similar to that of SP (Supplementary Table S3). The skin TKs are amphibian-specific, suggesting that TKs acquired new functions in the amphibian lineage.

As shown in Figure 4 and Supplementary Table S1, two TKs (CiTK-I, CiTK-II) were identified from the ascidian (protochordate), *Ciona intestinalis* (Satake et al., 2004). Like vertebrate TKs, CiTKs contain a C-terminal FXGLMamide (Satake et al., 2004), suggesting that the FXGLMamide sequence of TKs is highly conserved in Olfactores (vertebrates and ascidians). Since the ascidians are among the basal chordates, the *CiTk* gene is likely to correspond to a prototype of vertebrate *TK* genes. This single *CiTk* gene in *Ciona* encodes CiTK-I and CiTK-II (Satake et al., 2004; Figure 3B), suggesting that gene duplications of a prototype *TK* gene have occurred in the vertebrate lineage, resulting in *Tac1, Tac3, Tac4*, and amphibian skin *TK* genes. Furthermore, CiTK-I and -II are located in the same exon of the *CiTk* gene (Satake et al., 2004), indicating that splice variants of the *CiTk* gene are absent (Figure 4). Therefore, alternative splicing of the TK gene also emerged during vertebrate evolution.

**FIGURE 7:**
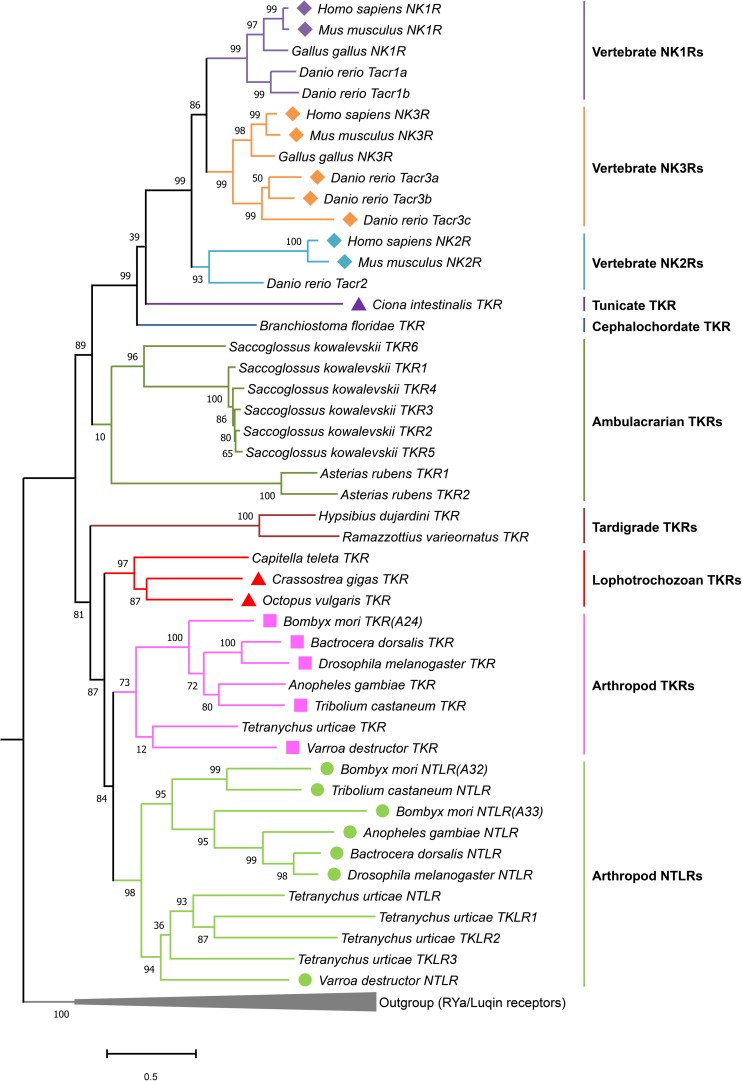
Phylogeny of tachykinin and natalisin receptors. RYamide and luqin receptors were used as outgroup to root the tree. Amino acid sequences of full-length receptors were used for the analysis. Sequences were aligned using the MAFFT (E-INS-i algorithm and BLOSUM30 scoring matrix) and the phylogenetic tree constructed using the FastTree plugin in Geneious Prime (2019). Receptors that have been functionally characterized are indicated by a symbol before the species name. Note that in another annelid, *Urechis unicinctus* (among Lophotrochozoans), the TK receptor has been functionally characterized (not shown here). The figure was constructed in MEGAX. Sequences used to generate the phylogeny are provided in Supplementary MaterialText File S1.

Homology search for mammalian NK1R - NK3R sequences using a *C. intestinalis* database^2^, showed that only one homologous receptor is present in the ascidian (Satake et al., 2004). This receptor, CiTKR, can be activated by CiTKs (Satake et al., 2004), indicating that it is an authentic TK receptor. Phylogenetic analysis reveals that the CiTKR is sister to the vertebrate TK receptor clade, which comprises NK1R - NK3R (Figure 7). These results suggest that NK1R – NK3R arose via duplication and diversification in the vertebrate lineage (Figure 7).

### TKs and Their Receptors in Insects and Other Protostome Invertebrates

Tachykinins have been identified in a wide range of invertebrates, including annelids, mollusks, arthropods, tardigrades, echinoderms and tunicates, and tentatively in nematodes (see Supplementary Table S1). Several of these were isolated biochemically, others cloned, but most were identified by bioinformatics and subsequently confirmed by mass spectrometry. However, many TKs listed in the Supplementary Table S1 have only been predicted from sequences identified from genomes and transcriptomes, based on similarity searches and await confirmation by mass spectrometry. There are some groups of invertebrates where TKs have not been identified or where sequences are only remotely similar to TKs. For instance in flatworms (Platyhelminthes) and cubomedusae (Cnidaria) no TKs have yet been discovered (McVeigh et al., 2009; Nielsen et al., 2019), and in sea anemones (Cnidaria) and nematodes (e. g. *C. elegans*) the “TK sequences” are not clearly TK-related (Ohno et al., 2017; Palamiuc et al., 2017; Hayakawa et al., 2019), see Supplementary Table S1. In fact, the proposed *C. elegans* TK-like receptor (NPR-22) is more closely related to RYamide/Luqin receptors than to TK-like receptors, and luqin-like peptides (from the LURY-1 precursor) are found in the worm and were shown to activate the receptor (Ohno et al., 2017; Yañez-Guerra et al., 2018). The previously proposed ligand (FMRFamide-like peptide 7, FLP-7) activates NPR-22 only at micromolar concentrations in a heterologous assay (Mertens et al., 2006; Palamiuc et al., 2017), suggesting that it is not a ligand [see (Ohno et al., 2017)]. However, it remains to be tested whether FLP-7 peptide is an NPR-22 ligand *in vivo*. Also, the *C. elegans* TK-like peptide derived from NPL-8 (SFDRMGGTEFGLM), does not activate the NPR-22 receptor (Mertens et al., 2006). Thus, the presence of a TK signaling system in *C. elegans* is still unresolved. However, TK-like receptors are found in Cnidaria (Anctil, 2009; Krishnan and Schiöth, 2015) (bioinformatics only), so the origin of TK signaling could possibly be traced to the common ancestor of Bilateria and Cnidaria. The presence of TK receptors has been demonstrated in the two major clades of Bilateria, the Nephrozoa (protostomes and deuterostomes) and its sister group the Xenacoelomorpha, that include Xenoturbella, Nemertodermatida, and Acoela (Thiel et al., 2018). In the Xenacoelomorpha, the presence is based on bioinformatics only.

With a few exceptions, each species has one gene encoding a TK precursor with multiple copies of TK peptides. As exceptions, two precursor genes were found in e.g., the limpet *Lottia gigantea* (Veenstra, 2010), the polychaete worm *Platynereis dumerilii* (Conzelmann et al., 2013), the crab *Carcinus maenas* (Christie, 2016), the tardigrade *Hypsibius dujardini* (Koziol, 2018) and the spider mite *Tetranychus urticae* (Veenstra et al., 2012; Supplementary Table S1). The organization of *Drosophila* and spider mite TK precursors is shown in Figure 4 and that of the mosquito *Aedes aegypti* in Figure 2B. The number of peptides that can be cleaved from invertebrate TK precursors range from 1 in tardigrades (Koziol, 2018; Figure 5) to 15 in the cockroaches *Leucophaea maderae* and *Periplaneta americana* (Predel et al., 2005). Commonly, these peptides all have different, but related sequences (designated paracopies). In a few cases, the precursor only has several identical TKs, like in the crayfish *Procambarus clarkii*, which has seven CabTRP1 (Yasuda-Kamatani and Yasuda, 2004). The TKs are generally between 9 and 11 amino acids long, but a few have only 6, and others up to 18 as in cockroaches (Muren and Nässel, 1996; Predel et al., 2005), or even 37 residues as predicted in some scorpions and spiders (Veenstra, 2016). Interestingly, the N-terminally extended TKs of cockroaches have internal dibasic cleavage sites and it appears as if in the brain these are more likely to be processed and, thus, generate the shortened TKs, whereas the extended TKs are normally found in the midgut (Muren and Nässel, 1996, 1997; Winther et al., 1999; Predel et al., 2005).

All TKs are amidated, but only very few have been detected that may be N-terminally blocked by pyroglutamate (pQ), for instance in hemipteran bugs and the bivalve mollusk *Anodonta cygnea* (Supplementary Table S1). In insects and many other arthropods, it is common to find TKs with an N-terminal proline (P) in the second position (e.g., *Drosophila* DTK-2, APLAFVGLRa). This is likely to render the peptides sensitive to proline-specific dipeptidyl peptidase (DPP-IV) cleavage and inactivation (Nässel et al., 2000; Isaac et al., 2009). Thus, TKs can be specifically inactivated by DPP-IV selectively located in regions of the CNS or in the periphery (Nässel et al., 2000). Other peptidases that have been shown to inactivate TKs are nephrilysins, angiotensin converting enzymes and deaminases [see (Isaac et al., 2002, 2009)].

Two putative TK receptors were cloned from *Drosophila* before the endogenous ligands were known (Li et al., 1991; Monnier et al., 1992). Both receptors displayed significant similarities to mammalian TK receptors. One of these, designated DTKR (CG7887) was confirmed as a receptor for endogenous *Drosophila* TKs (DTK-1-5) (Birse et al., 2006), the other NKD (CG6515) was first shown to respond to DTK-6, but not the other *Drosophila* TKs (Poels et al., 2009). DTK-6 has an FVAVRamide C-terminus instead of the common FX_1_GX_2_Ramide. Surprisingly, it turned out several years later that NKD is a receptor for a novel family of neuropeptides called natalisins (NTL) that in *Drosophila* have a consensus sequence of FX_1_X_2_X_3_Ramide (Jiang et al., 2013). One of these peptides, NTL4, has an FFATRamide, remotely similar to DTK-6, and indeed at high concentrations NTL4 activated the TK receptor DTKR (Jiang et al., 2013). The same authors also showed that DTK-6 at high concentrations activates the NTL receptor NTLR. In many insects, receptors of both DTKR- and NTLR-type have been identified (Jiang et al., 2013). In other invertebrates, such as for instance annelids (*Urechis*) and mollusks (*Octopus*) only receptors of DTKR type are known (Kawada et al., 2002; Kanda et al., 2007), suggesting that the NTL signaling system arose in the arthropod lineage (Jiang et al., 2013). However, TK-like precursors with NTL-like peptides are found in the spider mite (chelicerate) as well as tardigrades; the latter suggesting that the NTL signaling might also be present outside arthropods. Natalisin signaling will be discussed in a separate section.

Some invertebrate TKs act on exogenous TK receptors in prey animals. TKs with FQGMRa C-termini (Figure 2 and Supplementary Table S2) are produced by venom glands of the Jewel wasp *Ampulex compressa* and injected into the cockroach brain where action on the TK receptors leads to paralysis (Arvidson et al., 2019). Interestingly, these wasp venom TKs are injected as a precursor protein in the low pH venom, and not as cleaved peptides; only in the cockroach brain with neutral pH they will be slowly liberated to act on TK receptors (Arvidson et al., 2019). Other TKs with C-terminal FXGLMamide are produced in salivary glands of the mosquito *Aedes aegypti* (sialokinins I and II), and the cephalopods *Eledone moschata* (eledoisin) and *Octopus vulgaris* (OctTK-I and II) (Erspamer and Anastasi, 1962; Champagne and Ribeiro, 1994; Kanda et al., 2003). Presumably these TKs cause vasodilation in vertebrate prey animals [see (Erspamer and Erspamer, 1962; Beerntsen et al., 1999)]. The sequences of gland TKs are shown in Figure 2 and Supplementary Table S2.

### A Case of Novel Ligands for an Insect TK Receptor

Although most peptides and their cognate receptors co-evolve, there are a few interesting cases where receptors have adopted novel structurally unrelated ligands in addition to their original ligands. A well-known example is the *Drosophila* sex peptide, produced in male accessory glands and transferred to the female during copulation (Wolfner, 2002; Kubli, 2003). Sex peptide was adopted as an additional ligand for the myoinhibitory peptide (MIP) receptor (Kim et al., 2010). The *Drosophila* MIP receptor is the only known receptor for sex peptide. Another example, again in *Drosophila*, is the pigment-dispersing factor (PDF) receptor that has adopted DH31 as an additional ligand (Shafer et al., 2008; Goda et al., 2016). However, unlike sex peptide, DH31 also exerts its effects by binding to its own specific DH31 receptor (Johnson et al., 2005), suggesting that the PDF receptor is promiscuous. A similar phenomenon has been discovered for the silkmoth *Bombyx mori* TK receptor (BNGR-A24), which seems to have adopted ion-transport peptide (extended ITPL isoform in particular) as a novel additional ligand (Nagai-Okatani et al., 2016). *Bombyx* ITPL is a large protein (79 amino acids), comprising 6 cysteine residues, which form three disulfide bridges, and is thus structurally very dissimilar to tachykinins (Roller et al., 2008; Nagai-Okatani et al., 2016). Nonetheless, *Bombyx* ITPL and TKs appear to be orthosteric ligands of the *Bombyx* TK receptor (BNGR-A24) based on heterologous and homologous cell culture experiments (He et al., 2014; Nagai-Okatani et al., 2016). Moreover, activation of BNGR-A24 by ITPL is coupled to the cGMP pathway, whereas BNGR-A24 activation by TKs can activate different second messenger pathways in a cell-type specific manner. More specifically, TK-mediated activation of BNGR-A24 in BmN cells has no effect on cAMP and cGMP levels, but if the receptor is expressed in HEK293 and Sf21 cells causes an increase in cAMP and Ca^2+^ levels (He et al., 2014; Nagai-Okatani et al., 2016). Thus, the two ligands of the *Bombyx* TK receptor may activate distinct second messenger pathways, at least *in vitro*. Since ITP or ITPL receptors have not been identified in any other species besides *Bombyx*, it remains to be determined if this phenomenon is widespread amongst insects, or whether it is just restricted to *Bombyx*.

### TKs and Their Receptors in Deuterostome Invertebrates

TK-like receptors have also been mined from the genomes and transcriptomes of invertebrate deuterostome phyla such as Cephalochordata (e.g., *Branchiostoma floridae)*, Hemichordata (e.g., *Saccoglossus kowalevskii*) and Echinodermata (e.g., *Asterias rubens*) (Jekely, 2013; Mirabeau and Joly, 2013; Yañez-Guerra et al., 2018). Phylogenetic analysis suggests that TK-like receptors and luqin/RYamide-type receptors arose by gene duplication in a common ancestor of the Bilateria (Yañez-Guerra et al., 2018). Precursors encoding TK-like peptides have also been predicted in the starfish, *Asterias rubens*, and brittle stars (Figure 1 and Supplementary Table S1) (Semmens et al., 2016; Zandawala et al., 2017). However, the predicted TK-like peptides from *A. rubens* with an XXGL/IFamide C-terminus diverge substantially from FXGXRamide peptides of invertebrates, as well as from TKs of the protochordate *Ciona intestinalis* and vertebrates (FXGLMamide) (Semmens et al., 2016). Interestingly, the C-terminus of *A. rubens* (XXGL/IFamide) is somewhat similar to the proposed *C. elegans* tachykinin-like peptides (XMVRFamide) (Palamiuc et al., 2017), with peptides of both species having an Famide C-terminus and lacking the conserved phenylalanine residue in 5th position from the C-terminus. However, as mentioned above, the *C. elegans* TK-like receptor is more similar to RYamide/Luqin-like receptors, so it remains to be determined whether the predicted TK-like peptides in echinoderms are *bona fide* endogenous ligands for the echinoderm TK-like receptors. Moreover, sequences encoding TK-like receptors have been identified in the genomes and/or transcriptomes of hemichordates and cephalochordates (Jekely, 2013; Mirabeau and Joly, 2013; Figure 6). However, TK-like peptides have not yet been identified in these taxa. Perhaps the difficulty in discovering these peptides can be attributed to substantial diversification in the canonical sequence, which would render the homology-based search protocols ineffective.

## Evolution of Tachykinin Signaling Components

We show a cladogram of TKs (Figure 6) and phylogenetic analysis their receptors (Figure 7) in the animal kingdom and the occurrence of natalisin signaling in some groups. TK signaling is evolutionary ancient (Figure 6) and is one of several peptide families that emerged before the split of deuterostomes and protostomes (Jekely, 2013; Mirabeau and Joly, 2013). A few recent studies suggest that it might be more ancient than previously thought. TK-like receptors were recently found in genomes of Xenacoelomorpha, which is a sister group of Nephrozoa (comprising deuterostomes and protostomes) (Thiel et al., 2018). However, no TK-like ligands were identified in these genomes. A TK-like GPCR has also been predicted in the genome of the sea anemone *Nematostella vectensis*, but this receptor is more closely related to other *Nematostella* neuropeptide GPCRs than it is to bilaterian TK receptors (Krishnan and Schiöth, 2015; Thiel et al., 2018). Most protostomes have a single TK receptor but protostomian TK precursors encode multiple TK peptides (Figure 7). Thus, it appears that protostomian TKRs can all be activated by the different TKs, as shown already in *Drosophila* and *Bombyx* (Birse et al., 2006; Jiang et al., 2013; Nagai-Okatani et al., 2016). Our phylogenetic analysis shows that a single TK-like receptor is also found in tardigrades (Figure 7; Koziol, 2018). Interestingly, tardigrades have 2 TK-like precursors, one of which encodes a peptide with sequence similarity to TKs and another one, which encodes a NTL-like peptide (Figure 5; Koziol, 2018). This suggests that NTL signaling may have arisen by the duplication of TK gene first and a subsequent duplication and diversification of its receptor. Interestingly, the spider mite genome encodes multiple NTL-like receptors and a single TK receptor. In addition, it possesses two TK-like precursors, one of which contains TK-like and NTL-like peptides and another precursor with only TK-like peptides (Figure 5). This perhaps indicates a more advanced point in the diversification of the NTL-signaling, as the receptors now seem to have duplicated. Additional genomes of basal arthropods, onychophores and tardigrades need to be examined to determine the nature of TK-like and NTL-like signaling present in these animals before we can establish the precise lineage in which the NTL-signaling arose.

In deuterostomes, at least ancient Olfactores (vertebrates and ascidians) acquired a TK receptor that recognized a TK harboring the C-terminal FXGLMamide motif. The three subtypes of TK receptors, namely NK1R, NK2R, and NK3R, appear to have arisen following the whole genome duplications in the vertebrate lineage. Furthermore, these subfamilies might have acquired ligand selectivity during their diversification along with the generation of TK subtypes. The current missing pieces are echinoderm, acorn worm, and amphioxus counterparts, because canonical TKs have not yet been identified in genomes and/or transcriptomes of these deuterostome invertebrates. However, multiple TKRs are present in echinoderms and hemichordates suggesting additional independent gene duplication events within these lineages. In Supplementary Figure S1 we show multiple sequence alignments of select TK and natalisin receptors.

## Tachykinins in Invertebrates, Distribution and Functions

### Overview of Functional Diversity From Early Studies

The first TKs isolated from insects were purified with the aid of a hindgut contraction assay that had also been utilized for first discovery of numerous other insect neuropeptides (Holman et al., 1990, 1991; Schoofs et al., 1990b). TKs from the annelid worm *Urechis unicinctus* were also purified with the aid of a muscle contraction assay (Ikeda et al., 1993). Thus, it was shown early that TKs are myostimulatory on a variety of muscles in the body wall, oviduct, foregut, hindgut, as well as heart [see (Ikeda et al., 1993; Schoofs et al., 1993; Nässel, 1999; Sliwowska et al., 2001)]. Examples of other functions established before employment of genetic tools are modulation of network activity in the stomatogastric ganglion of crustaceans [(Blitz et al., 1995), reviewed in Nusbaum and Blitz (2012), Nusbaum et al. (2017)], activation of dorsal unpaired median neurons in locust (Lundquist and Nässel, 1997), stimulation of release of adipokinetic hormone from locust corpora cardiaca (Nässel et al., 1995b), diuretic action on Malpighian tubules of locust *L. migratoria* and moth *Manduca sexta* (Skaer et al., 2002; Johard et al., 2003) and presynaptic inhibition of crayfish photoreceptors, likely as a co-transmitter of GABA (Glantz et al., 2000). From numerous *in vitro* studies, there is no evidence that the different paracopies of TKs in a species have any major differential activities or functions, except possibly DTK-6 in *Drosophila*, but it should be noted that the presence of this mature peptide has not been verified by mass spectrometry. Additional functions of TKs discovered using various approaches are discussed separately in the context of different species in sections “Distribution and Function of TKs in Invertebrates” and “Functional Roles of TKs in Drosophila, Genetic Advances”.

### Distribution and Function of TKs in Invertebrates

Early work used antisera to substance P and other vertebrate tachykinins to localize presumptive TK neurons in the CNS of several invertebrates summarized in Nässel (1999). It should be noted that the earliest of these studies were performed before neuronal/intestinal TKs had been isolated and sequenced in invertebrates. In retrospect, it appears that of the TK antibodies used during this era, one monoclonal antibody (Cuello et al., 1979) actually recognizes the invertebrate TKs [see (Blitz et al., 1995; Johansson et al., 1999; Nässel, 1999)], whereas the polyclonal ones, except anti-Kassinin (Lundquist et al., 1994), seem to label other epitopes, at least in insects. Thus, TK distribution in several crustaceans (Sandeman et al., 1990; Schmidt and Ache, 1994; Blitz et al., 1995; Johansson et al., 1999), and the horse shoe crab *Limulus polyphemus* (Chamberlain and Engbretson, 1982; Mancillas and Selverston, 1985) is likely to be correctly described in these earlier studies. The first antisera to invertebrate TKs were raised against locust LomTK-I (Nässel, 1993) and LomTK-II (Vitzthum and Homberg, 1998), blowfly CavTKII (Nässel et al., 1995a) and cockroach LemTRP1 (Winther and Nässel, 2001) and these were subsequently used in a large number of invertebrate species, some of which are outlined below.

#### Insects

The neuronal distribution of TK immunoreactivity in the CNS is in general fairly well conserved between insects studied, whereas in other arthropods only some features seem to be shared with insects. Characteristic of TK distribution in insects is presence in neuronal processes in antennal lobes, central complex, pars intercerebralis, dorsolateral protocerebrum, optic lobes and subesophageal zone. First, we will outline the neuronal localization of TK in *Drosophila* and a few other insects, then move on to crustaceans, and snails. For these organisms, except *Drosophila*, we also briefly describe TK functions. Functional roles of TK signaling in *Drosophila* are described in a separate section.

**FIGURE 8:**
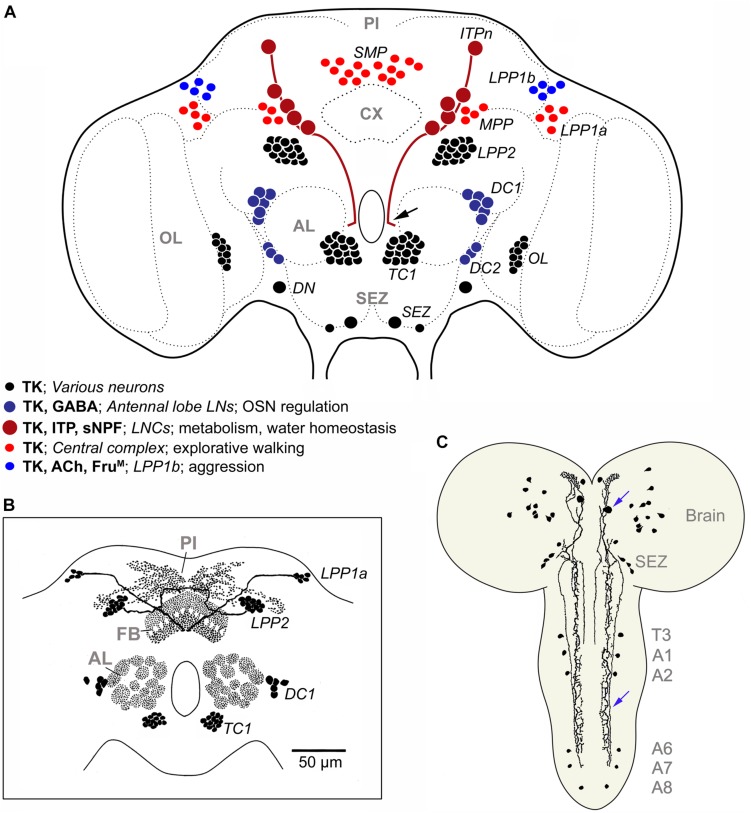
TK in the *Drosophila* brain. **(A)** Schematic of neuronal TK distribution in the adult *Drosophila* brain (frontal view). Neuronal cell bodies are shown in different colors (see legend in figure) to indicate those that have been studied functionally in some detail (blue and red shades), versus those that remain unexplored (black). The light red neurons (*SMP, MPP, LPP1a*) innervate different layers of the fan-shaped body of the central complex (Kahsai and Winther, 2011) and modulate explorative walking (Kahsai et al., 2010b). The dark blue ones (*DC1, DC2*) are local neurons of the antennal lobe, some of which coexpress GABA, and are part of circuitry that regulates odor sensitivity in olfactory sensory neurons (OSNs) (Ignell et al., 2009). In male flies the light blue neurons (*LPP1b*) express Fru^*M*^ and probably acetylcholine (Ach) and regulate levels of aggression (Asahina et al., 2014). The dark red ones (ITPn) are lateral neurosecretory cells (LNCs) with axon terminations in the corpora cardiaca-corpora allata, anterior aorta and intestine (Kahsai et al., 2010a). These cells (*ITPn*) co-express TK, ion transport peptide (ITP) and short neuropeptide F (sNPF) and regulate aspects of metabolic and water homeostasis (Kahsai et al., 2010a; Galikova et al., 2018). Arrow indicates axon destined for retrocerebral complex of the black neurons *LPP2* and *TC1* neurons send axons to the pars intercerebralis (PI) and dorsal protocerebrum (Lundquist et al., 1994; Winther et al., 2003), the DNs were assumed to be descending neurons (Winther et al., 2003), and resemble natalisin-producing ICLI neurons shown in Supplementary Figure S5 (Jiang et al., 2013). The branching of the neurons associated with the optic lobes (OL) and subesophageal zone (SEZ) has not been unraveled. The terminology (except *ITPn*) is from Winther et al. (2003) and specification of neurons compiled from papers cited above. **(B)** Schematic of TK distribution in some neuropil regions of the *Drosophila* brain. FB, fan-shaped body, other acronyms as in **(A)**. Modified from Nässel (2002). **(C)** TK immunoreactive neurons in brain and ventral nerve cord of third instar larva of *Drosophila*, slightly edited from Winther et al. (2003). Blue arrows indicate a descending neuron. T3, third thoracic neuromere; A1-A8 abdominal neuromeres.

In the adult *Drosophila* brain, *in situ* hybridization and immunolabeling revealed that there are more than 160 TK-expressing neurons that can be divided up into 11 bilateral groups and one unique pair (Figure 8A; Winther et al., 2003). Ten large lateral neurosecretory cells (*ITPn*) express TK, as well as two other peptides (short neuropeptide F, sNPF, and ion transport peptide, ITP) (Kahsai et al., 2010a). The other TK neurons are interneurons of different kinds innervating the fan-shaped body of the central complex, the antennal lobes, the optic lobes, pars intercerebralis, dorsal lateral protocerebrum and the subesophageal zone (Winther et al., 2003). Some of these TK neuron clusters have been functionally investigated by genetic manipulations (colored cells in Figure 8A), whereas the functions of other clusters (black cells in Figure 8A) remain obscure. Functional aspects will be discussed later in a separate section. Details of some of the TK neurons are shown in Figure 8B. In the third instar larva there are only about 44 neurons in the entire CNS that are consistently labeled by TK antisera; 32 of these are in the brain and SEZ (Figure 8C) (Siviter et al., 2000; Winther et al., 2003). In both larvae and adults, enteroendocrine cells of the midgut and anterior hindgut express TK (Siviter et al., 2000; Veenstra et al., 2008; Veenstra, 2009).

In some cells in *Drosophila*, TK is colocalized with other neuropeptides or GABA (Supplementary Table S4): in neurosecretory cells (ITPn) with sNPF and ITP, in local neurons of the antennal lobe with either GABA, allatostatin-A or myoinhibitory peptide (MIP; also known as allatostatin-B) and in midgut enteroendocrine cells with either neuropeptide F or diuretic hormone 31 (Veenstra et al., 2008; Ignell et al., 2009; Carlsson et al., 2010; Kahsai et al., 2010a). In addition, single cell transcriptome sequencing of brain neurons shows that TK is coexpressed with glycoprotein hormone beta 5 (GPB5) (Davie et al., 2018). A more systematic screen of colocalized substances in the insect CNS would probably make this list longer.

The blowfly *Calliphora vomitoria* displays a neuronal distribution of TK very similar to that in *Drosophila* (Lundquist et al., 1994). Studies of TK distribution in other insects reveal many similarities, except that the numbers of neurons in the different clusters vary between species, as shown next.

In the brain of the honeybee *Apis mellifera* TK distribution was mapped by *in situ* hybridization (Takeuchi et al., 2004). Neuronal cell bodies were revealed in association with the central complex, antennal lobes and optic lobes, as in other insects, but also associated with the mushroom body calyces. The intrinsic mushroom body neurons were identified as the small-type Kenyon cells (class I and II) (Takeuchi et al., 2004). Later, immunolabeling also confirmed presence of TK in Kenyon cells including their axons in the lobes (Heuer et al., 2012). The distribution of TK transcript is spatially similar irrespectively of sex, cast, or division of labor of workers: however, quantitatively transcript levels are higher in queens and foragers than in nurse and drone bees (Takeuchi et al., 2004). In bees, the TK in mushroom bodies may be involved in regulation of foraging and social behaviors (Takeuchi et al., 2004; Brockmann et al., 2009; Boerjan et al., 2010). Also in other hymenopterans (Oya et al., 2017) and in the beetle *Tribolium castaneum* (Binzer et al., 2014), TK was identified in major subpopulations of Kenyon cells, but in other studied insects there are so far no reports of such neurons producing TKs. In the honeybee, quantification of neuropeptides by mass spectrometry was performed after foraging nectar or pollen (Brockmann et al., 2009). TK was among the three peptides whose levels were most affected in association with foraging for nectar or pollen.

In the moth *Spodoptera litura*, at least 80 TK neurons were detected in the adult brain, and the innervation of the central complex, the antennal lobes, pars intercerebralis, dorsal lateral protocerebrum and the subesophageal zone is similar to that in *Drosophila* (Kim et al., 1998; see Supplementary Figure S2). Also a pair of large descending neurons was identified. A special feature of the moth is the presence of TK expression in 8 median neurosecretory cells with axon terminations in the retrocerebral complex and anterior aorta (Kim et al., 1998). Similar median neurosecretory cells (MNCs) were also seen in the moth *Heliothis virescens* (Zhao et al., 2017), and the beetles *Tenebrio molitor* and *Zophobas atratus* (Sliwowska et al., 2001). In another moth *Manduca sexta*, the TK distribution in the brain (except the MNCs) and intestine was found similar to *S. litura* and it was shown that TK stimulates secretion in the Malpighian tubules *in vitro* (Skaer et al., 2002).

The brains of the cockroach *Leucophaea maderae* and the locust *Locusta migratoria* contain far larger numbers of TK neurons, but the innervation pattern of brain regions is similar to *Drosophila* and moth (Nässel, 1993; Muren et al., 1995; Vitzthum and Homberg, 1998). In the *L. maderae* brain (without optic lobes), about 360 TK neurons were found (Muren et al., 1995; Supplementary Figure S3), and about 800 in the entire brain of *L. migratoria* (Nässel, 1993). In contrast to *Drosophila*, there are efferent TK neurons in the cockroach abdominal ganglia that innervate the hindgut and TK neurons in the stomatogastric ganglia that supply extensive axon terminations over the foregut and midgut (Muren et al., 1995; Nässel et al., 1998). In locusts, TK neurons in the lateral neurosecretory cell group send axons to the corpora cardiaca where they contact cells producing adipokinetic hormone (AKH) and it was shown that TKs induce AKH release *in vitro* (Nässel et al., 1995b). These neurosecretory cells may be analogous to the ITPn neurons in *Drosophila* (Kahsai et al., 2010a), although a role in hormone release was not yet analyzed in the fly. In both locust and cockroach midgut, endocrine cells express TK, and in the locust there are also TK producing endocrine cells in the six midgut ampullae at the base of the Malpighian tubules (Muren et al., 1995; Winther and Nässel, 2001). TKs stimulate secretion in locust Malpighian tubules (Johard et al., 2003). Calcium-dependent release of TK from the cockroach and locust intestine could be induced by potassium application, and TK was demonstrated in hemolymph, suggesting that hormonal release of intestinal TK regulates tubules secretion (Winther and Nässel, 2001). In locusts, several cases of colocalization of TK and other peptides have been demonstrated. The endocrines of the ampullae (but not in the rest of the midgut) coexpress TK, diuretic hormone 44 (DH44) and FMRFamide-like peptide, and TK was shown to stimulate secretion in locust Malpighian tubules together with DH44 (Johard et al., 2003). In certain central complex neurons there is co-expression of TK and leucokinin and in others TK and octopamine or GABA (Vitzthum and Homberg, 1998). Finally, sensory neurons of the metathoracic legs co-express TK, allatotropin, FMRFamide-like peptide and probably acetylcholine (Persson and Nässel, 1999; Supplementary Table S4).

Another insect studied in some detail is the hemipteran blood-sucking bug *Rhodnius prolixus* where a total of about 250 TK immunoreactive neurons were found in the brain (Kwok et al., 2005). These are distributed in the optic lobes and in several other clusters in the midbrain. Interestingly, no TK containing enteroendocrine cells were detected in this species, in contrast to many other studied insects, but the hindgut is innervated by TK axons (Kwok et al., 2005). These TK axons also express leucokinin, another myostimulatory peptide (Haddad et al., 2018). *Rhodnius* TKs were shown to increase the basal tonus of the hindgut, but also to increase the frequency and amplitude of peristaltic contractions of the salivary gland, a tissue that displays high levels of TK transcript, but no immunoreactive TK (Haddad et al., 2018).

Some further functions of TKs in insects other than *Drosophila* have been explored that might shed light on TK signaling in general. In female burying beetles, *Nicrophorus vespilloides*, neuropeptides were quantified in solitary virgins, individuals actively parenting or post-parenting solitary adults to identify neuropeptides associated with parenting (Cunningham et al., 2017). TK was found as one of the few peptides associated with active parenting. In several insects, including *Drosophila*, oriental fruitfly, cockroaches and moths, TK seems to play a role in modulation of olfactory sensory processing (Ignell et al., 2009; Jung et al., 2013; Fusca et al., 2015; Ko et al., 2015; Gui et al., 2017b; Lizbinski and Dacks, 2017). Other functions have not been investigated in multiple species; however, immunocytochemistry suggests some conservation of the distribution of TK in neurons of specific brain centers, and intestine in insects and crustaceans that might also reflect functional conservation.

#### Crustaceans

The TK distribution in the brain of a few crayfish, lobster and crab species has been studied, mostly with a monoclonal antibody to substance P (Goldberg et al., 1988; Sandeman et al., 1990; Schmidt and Ache, 1994; Schmidt, 1997a, b), but one study also employed antiserum to a cockroach TK that is nearly identical to crab TK (Johansson et al., 1999). These studies have mostly focused on TK neurons in the olfactory centers of the brain, but also the stomatogastric nervous system.

As seen in Supplementary Figure S4, brain of crayfishes possesses a pair of TK interneurons with large cell bodies and extensive processes in the anterior deutocerebrum and varicose branches among the cell bodies of a group of olfactory interneurons in the lateral deutocerebrum (Sandeman et al., 1990; Johansson et al., 1999). There is another pair of TK neurons with deutocerebral cell bodies and processes in the neuropil of the olfactory lobe, as well as larger numbers of small TK neurons with processes in the olfactory and accessory lobes (Sandeman et al., 1990; Johansson et al., 1999). TK neurons were shown in all the neuropils of the optic lobes of the crayfish and specifically a set of TK and GABA expressing amacrine cells were identified in the lamina ganglionaris (Glantz et al., 2000). This study shows that application of GABA and TK to photoreceptor terminals in the lamina induces a short-latency, dose-dependent hyperpolarization with a decay time of a few seconds. TK also acts over several minutes to reduce the photoreceptor potential to potentiate the action of GABA (Glantz et al., 2000). In the American lobster, the distribution of TK processes is in general similar to that seen in insects with TK immunolabeling in the protocerebral bridge, central body, olfactory (antennal) lobes, and anterior median protocerebral neuropil (Langworthy et al., 1997). Like in insects, midgut enteroendocrine cells in crabs also express TK (Christie et al., 2007).

In decapod crustaceans the stomatogastric nervous system (STN) consists of 25–30 neurons (depending on species) and controls handling of ingested food. Most of these neurons contribute to the activity in one or both of the neural networks in the STN, which regulate (1) gastric mill (chewing) or (2) pyloric circuit (pumping and filtering of food that has been chewed) [see (Nusbaum et al., 2017)]. A pair of neurons (MCN1) that innervate the stomatogastric ganglion produces the TK CabTRP-Ia (Blitz et al., 1995; Christie et al., 1997). The MCN1s also produce GABA and the peptide proctolin (Nusbaum et al., 2017). It was shown that CabTRP-Ia and GABA released from MCN1 are critical for activation of the gastric mill rhythm, whereas MCN1 release of CabTRP-Ia and proctolin predominantly excites the pyloric rhythm (Nusbaum et al., 2017).

#### Mollusks

A few mollusks have been investigated with respect to TK distribution (using antiserum to locust TK). These include the pond snail *Lymnaea stagnalis*, the pulmonate terrestrial snail *Helix pomatia* and the freshwater bivalve, *Anodonta cygnea* (Elekes and Nässel, 1994; Elekes et al., 1995). In *L. stagnalis*, about 180 TK neurons were found, distributed in cerebral and pedal ganglia and TK axons were detected in the intestine (Elekes et al., 1995). About 900 TK neurons were seen in *H. pomatia* with about 80% of these in cerebral ganglia, whereas in *A. cygnea* only a smaller number of TK neurons was detected in cerebral, pedal and visceral ganglia (Elekes and Nässel, 1994; Elekes et al., 1995). In the snails, a large number of TK neurons are located in procerebrum of the cerebral ganglia (Supplementary Figure S5). The procerebrum is an association center for olfactory information similar to the mushroom bodies of insects and thus TKs seem to be involved in olfactory processing also in mollusks (Elekes and Nässel, 1994). Recently, a TK receptor related to the *Drosophila* DTKR and responding to endogenous TKs was identified in the bivalve mollusk *Crassostrea gigas* (Dubos et al., 2018). In the snail *Helix*, the neuronal membrane effects of locust LomTK-I, and anodontatachykinin, were either depolarizing or hyperpolarizing depending on neuron-type, and voltage-clamp experiments revealed a role of Ca- or K-currents in these peptide effects (Elekes et al., 1995).

#### Nematode Worms

In *Caenorhabditis elegans*, the gene FLP-7 was considered to encode a TK precursor ortholog (Palamiuc et al., 2017). However, as mentioned in section “TKs and Their Receptors in Insects and Other Protostome Invertebrates,” this gene encodes FMRFamide-like peptides (Mertens et al., 2006) and the proposed receptor gene (NPR-22) is only remotely related to TK receptors, and more closely related to the RYamide/Luqin receptor (Ohno et al., 2017; Yañez-Guerra et al., 2018; see Figure 6). Nevertheless, since the signaling system was referred to as a TK system (Palamiuc et al., 2017) we summarize the findings here. It was shown that FLP-7 is expressed in several tissues, including the head, the nervous system, and the sensillum (wormbase.org). At the cellular level, a fluorescent transgenic reporter line revealed that FLP-7 is expressed in the ALA motor- and the AVG interneurons and in the ASI sensory neurons, and that the reporter is secreted into the “circulation” (Palamiuc et al., 2017). The ALA motor neuron has been shown to regulate locomotion, the AVG neuron influences ventral cord development, and the ASI sensory neuron pair regulates whole body physiology during development, controls lifespan via neurohormones, and regulates 5-HT-induced fat loss (Palamiuc et al., 2017). FLP-7 was shown to act in the intestine to induce lipase activity and fat loss (Palamiuc et al., 2017). In another study, the ligands of NPR-22 were found to be the luqin-like peptides LURY-1 and 2 (AVLPRYa and PALLSRYa) encoded on the gene Y75B8A.11 (Ohno et al., 2017). The LURY peptides are secreted from pharyngeal neurons and regulate feeding, lifespan, egg-laying and locomotor activity (Ohno et al., 2017). In summary, no clear-cut TK signaling system has been discovered in *C. elegans* so far.

#### Ambulacraria

Tachykinin expression has not been mapped yet in echinoderms or hemichordates.

### Functional Roles of TKs in Drosophila, Genetic Advances

With the introduction of the binary Gal4-UAS system (Brand and Perrimon, 1993) it became possible to genetically target components of the TK signaling system spatially and temporarily, and thus knock down or increase activity in a neuron-specific fashion. Table 2 summarizes the known functions of TKs in *Drosophila*. A first study, utilized *Tk*-RNAi to broadly knock down TK production in neurons by means of ubiquitously expressed drivers (*Elav-* and *tubulin-Gal4*) and monitor effects on olfaction and locomotion (Winther et al., 2006). The flies with globally reduced TK signaling displayed decreased responses to certain odors and were hyperactive in locomotor assays. Subsequent studies describe more targeted manipulations where TK functions in smaller populations of neurons could be revealed.

**TABLE 2:** Functions of TKs in *Drosophila*.

**Neurons targeted^1^**	**Functional TK role indicated**	**References**
Global Tk-RNAi	Modulation of odor sensitivity	Winther et al., 2006
Global Tk-RNAi	Modulation locomotor activity	Winther et al., 2006
OSNs in AL (Dtkr)	Presynaptic inhibitory feedback to OSNs	Ignell et al., 2009
OSNs in AL (Dtkr)	Starvation-induced increase in odor sensitivity	Ko et al., 2015
Neurons in SEZ (Tk)	Modulation of pheromone response (via Gr68a)	Shankar et al., 2015
Central complex (Tk)	Modulation of explorative walking	Kahsai et al., 2010a
Brain neurons (Tk)	Modulation of aggression level (fruitless neurons)	Asahina et al., 2014
IPCs (Dtkr)	Regulation of insulin production	Birse et al., 2011
Nociceptive cells (Dtkr)	Modulation of nociception in sensory cells	Im et al., 2015
ITPn (brain NSCs; Tk)	Regulation of metabolic stress responses	Kahsai et al., 2010a
ICNs (brain neurons)^2^	Inhibit larval IPCs, affect growth via IIS and EGF	Meschi et al., 2019
Endocrines in gut (Tk)	Regulation of lipid metabolism in intestine	Song et al., 2014
TK endocrines in gut^3^	IMD-mediated DILP upregulation; organismal growth	Kamareddine et al., 2018
*In vitro* TK application	Induces midgut contraction	Siviter et al., 2000
*In vitro* TK application	Modulation of heart contraction rate	Schiemann et al., 2018

*^1^In brackets: genetic interference with peptide (Tk) or receptor (Dtkr). ^2^Ablation of TK-expressing ICN neurons, activation or inactivation of neurons. ^3^Interference with signaling in TK expressing endocrines. OSN, olfactory sensory neurons; AL, antennal lobe; SEZ, subesophageal zone; IPC, insulin-producing cells; ITPn, ITP neurons; NSC, neurosecretory cell; ICN, IPC contacting neurons; EGF, epidermal growth factor; IMD, immune-deficiency pathway; DILP, *Drosophila* insulin-like peptide.*

**FIGURE 9:**
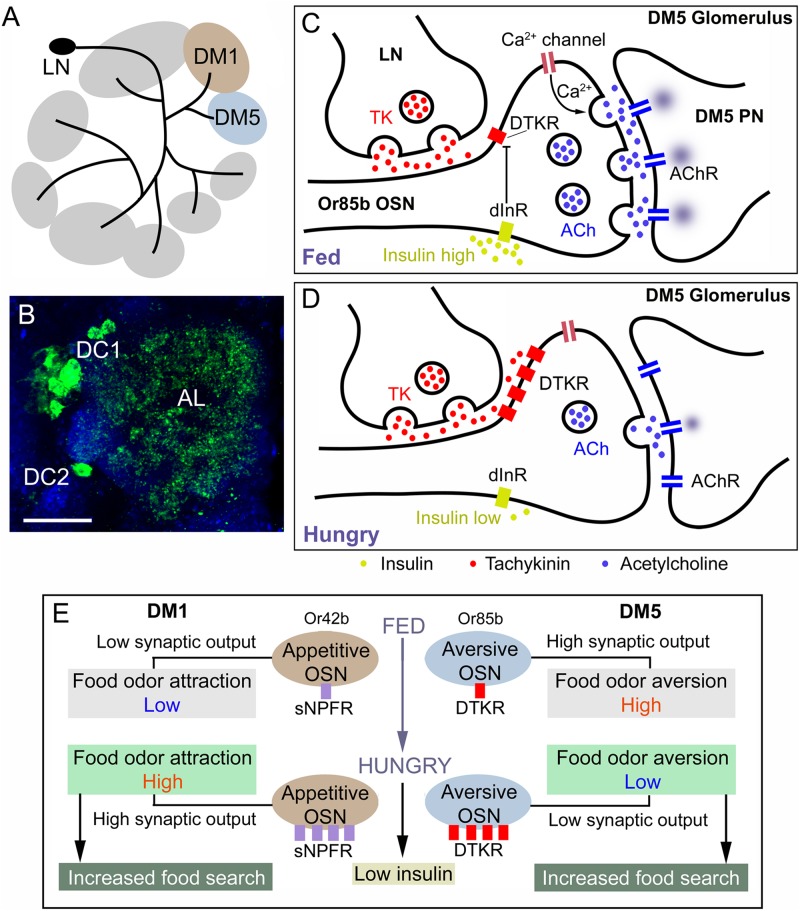
Role of TK signaling in food odor sensing in the antennal lobe of *Drosophila*. **(A)** TK peptides are expressed in local neurons (LN) of the antennal lobe and innervate most glomeruli. Two glomeruli are shown here (DM1 and DM5). Of these DM1 is mediating food odor attraction (Or42b) and DM5 food odor aversion (Or85b). **(B)** Image of TK immunoreactive LNs (green) in the clusters DC1 and DC2 innervation antennal lobe (AL). **(C)** Role of TK signaling in the DM5 glomerulus, which relays aversive odor signals from olfactory sensory neurons (OSN) that express odorant receptors Or85b to DM5 projection neurons (PN) which in turn signal to higher order neurons that control food search. In the fed fly the circulating level of insulin-like peptide (DILP) is high, which suppresses expression (transcription) of the TK receptor DTKR. When DTKR signaling is low there is no suppression of Ca^2+^ channel activity and therefore release of acetylcholine (ACh) is strong when the OSN is activated and as a consequence the DM5 PN relays strong aversive signals and food search is reduced. **(D)** In the hungry fly the DILP level is low, DTKR expression is high and therefore TK signaling activates DTKR and the OSN releases less ACh resulting in suppressed activation of the aversive DM5 PN and therefore increase food search. **(E)** A scheme showing the combined signaling from the DM1 and DM2 signaling pathways that increase food search in hungry flies with low circulating insulin. In hungry flies the aversive DM5 odor pathway is inactivated resulting in increased food search (as detailed in Figure 7C). In the DM1 pathway (food odor attraction) signaling with short neuropeptide F (sNPF) and its receptor sNPFR is increased in hungry flies due to low insulin and increased expression of the sNPFR. This leads to presynaptic potentiation of the ACh signaling and increased activation of DM1 PNs, resulting in increased food search. The panels **(A,C–E)** were redrawn from figures in Ko et al. (2015) and Jékely et al. (2018).

It was found that the TK receptor DTKR is expressed by olfactory sensory neurons (OSNs) of the *Drosophila* antennae and TK in subpopulations of the local neurons (LNs) of the antennal lobe (Ignell et al., 2009). TK signaling from LNs to OSNs provides presynaptic inhibitory feedback by suppressing calcium and synaptic activity (Ignell et al., 2009). An ensuing study revealed further details on the role of TK signaling in olfaction and food search (Ko et al., 2015). It was shown that in hungry flies where circulating levels of insulin-like peptide (ILP) are low there is an upregulation of the DTKR in OSNs carrying specific odorant receptors (Or42b and Or85a) (Figure 9). In the antennal glomerulus DM5, which conveys food odor aversion (negative valence), upregulation of the inhibitory DTKR in a hungry fly leads to increased TK signaling and thus suppressed depolarization and as a consequence decreased synaptic activation of antennal lobe projection neurons (PNs) leading to increased food attraction (Ko et al., 2015). When the fly has fed, and circulating insulin is high, the DTKR expression decreases due to activation of the insulin receptor in OSNs, synaptic signaling increase and food aversion is augmented (Figure 9). In glomerulus DM1 (positive valence; wired for food odor attraction) innervated by Or42b expressing OSNs, enhanced signaling with sNPF increases food attraction in hungry flies with low circulating insulin (Ko et al., 2015). This enhanced signaling is caused by up-regulation of sNPF receptor expression on OSNs and strengthened synaptic activation of PNs (Figure 9). Together, peptidergic neuromodulation of the two odor channels (DM1 and DM5) ensures that hungry flies increase food search. Whereas it has been shown that sNPF facilitates cholinergic transmission in OSNs to PNs (Ko et al., 2015), it is not clear whether TK acts to modulate inhibitory GABA transmission in LNs (Ignell et al., 2009). Also in the cockroach *Periplaneta americana* (Jung et al., 2013) and the oriental fruitfly *Bactrocera dorsalis* (Gui et al., 2017b), TK signaling modulates olfactory sensitivity, and the presence of TK in antennal lobe neurons in all studied insects may suggest a conserved role in olfaction.

In the central complex of *Drosophila*, TK is found in a few sets of neurons (light red neurons in Figure 8A) and in assays of explorative walking TK knockdown in some of these neurons resulted in flies with increased center zone avoidance, whereas knockdown in other neurons resulted in flies with increased activity-rest bouts (Kahsai et al., 2010b). Thus, TK in the central complex seems to be important for modulation of spatial orientation, activity levels, and temporal organization of spontaneous walking.

A small set of protocerebral TK neurons (light blue in Figure 8A) have been shown to regulate levels of aggression in male *Drosophila* (Asahina et al., 2014). These TK neurons are a small subpopulation of the numerous neurons that express the male splice form of fruitless (FruM^+^), a transcription factor that specifies male-specific behavior, including male aggression. Thus, a set of 4 pairs of neurons in the brain designated Tk-GAL4^*FruM*^ neurons control the level of male-male aggression, but have no influence on male-female courtship behavior (Asahina et al., 2014). The same authors found that the Tk-GAL4^*FruM*^ neurons also may be cholinergic (express marker for acetylcholine signaling) and that this neurotransmitter may thus play an additional role in the circuit.

Another male-specific TK circuit in *Drosophila* is involved in gustatory detection of an anti-aphrodisiac pheromone (CH503). Gustatory cells (Gr68a) in the forelegs respond to this pheromone and mediate signals to central brain circuits via 8 to 10 TK neurons located in the subesophageal zone and thereby suppress courtship (Shankar et al., 2015). It is not clear from this study to which specific neurons TK in Figure 8 they correspond.

The insulin-producing cells (IPCs) of the *Drosophila* brain are modulated by several factors, including TK (Birse et al., 2011; Nässel and Vanden Broeck, 2016). The IPCs produce four insulin-like peptides (DILP1, 2, 3, and 5) and are known to regulate many aspects of development and adult physiology, such as growth, metabolism, stress responses, reproduction and lifespan reviewed in Owusu-Ansah and Perrimon (2014), Nässel and Vanden Broeck (2016). Knockdown of the receptor DTKR in IPCs affected levels of *dilp2* and *dilp3* transcripts in these cells, increased the fly lifespan and diminished carbohydrate levels during starvation (Birse et al., 2011). Knockdown of the natalisin receptor (NTLR; CG6115; earlier known as NKD) had no effect on IPC activity and the TK cells acting on the IPCs were not identified (Birse et al., 2011). In a more recent paper, a pair of TK neurons was demonstrated in the *Drosophila* larva, which connect functionally to the IPCs (Meschi et al., 2019). These TK neurons (ICNs) are inhibitory on IPCs. Under protein-rich diet conditions the ICNs respond to growth-blocking peptides secreted from the larval fat body and this alleviates the inhibitory action on IPCs, and DILPs can be released to stimulate growth (Meschi et al., 2019). It is not completely clear from the images of this paper, but it appears as if the ICNs are the same as the descending neurons shown in Figure 8C (blue arrows), which also exist in the adults (DN in Figure 8A).

In larvae, a nociceptive pathway mediating thermal tissue damage signals was identified and shown to include TK and the receptor DTKR (Im et al., 2015). The DTKR receptor is expressed in the nociceptive sensory neurons and required for mediation of thermal hypersensitivity after tissue damage. A set of TK expressing interneurons in the ventral nerve cord mediates this presynaptic modulation of nociceptive sensory neurons (Im et al., 2015). Substance P is known for its role in modulation of nociceptive sensory signals in the dorsal horn of the spinal cord [see (Hökfelt et al., 2001; Steinhoff et al., 2014)], suggesting a conserved role of tachykinin signaling, although the pathway and mechanisms differ.

A set of five pairs of large neurosecretory cells (ITPn in Figure 8A) produces TK, as well as ITP and sNPF. Targeted knockdown of TK (or sNPF) in these cells result in flies that display decreased survival time when exposed to desiccation or starvation, and also suffer increased water loss at desiccation (Kahsai et al., 2010a). ITP is acting as an antidiuretic hormone (Galikova et al., 2018), but it is not likely that TK or sNPF are released as circulating hormones from the ITPn cells (Kahsai et al., 2010a). Instead, these peptides might act locally, either presynaptically on ITPn axon terminations, or on other brain neurons/neurosecretory cells to modulate antidiuretic signals or metabolic stress responses. A similar local action of sNPF released from lateral neurosecretory cells in the brain has been demonstrated; it was found that the IPCs in the brain and the AKH-producing cells in the CC were directly regulated by locally released sNPF (Kapan et al., 2012; Oh et al., 2019).

In gut endocrine cells (EECs) of *Drosophila*, TK was shown to influence lipid homeostasis by controlling lipid production in enterocytes of the midgut (Song et al., 2014). These TK- (and DH31-) producing EECs are nutrient-sensing and can be activated by the presence of circulating dietary proteins and amino acids (Park et al., 2016). The EECs have also been shown to play a role in the innate immune system and development of *Drosophila* (Kamareddine et al., 2018). Activating the immune deficiency (IMD) pathway in EECs triggers TK signaling leading to DILP3 upregulation in the gut and mobilization of lipids increased insulin signaling and effects on organismal development and growth. Thus, the gut microbiota can influence growth via the immune system and TK and insulin signaling (Kamareddine et al., 2018). TK was also shown to activate peristalsis in the midgut (Siviter et al., 2000), maybe by local paracrine signaling.

### Functional Roles of TKs in Protochordates and Non-mammalian Vertebrates

Also in several non-insect invertebrates and non-mammalian vertebrates TKs were found to exhibit contractile activity on muscles in the digestive tract (Satake and Kawada, 2006; Satake et al., 2013; Steinhoff et al., 2014). In the following we will discuss additional roles of TKs in sea squirts (Ascidians) and fish, exemplified by Zebrafish.

#### Ascidians

Aoyama et al. (2012) demonstrated that CiTK induces growth of follicles in *Ciona* during late stage-II (vitellogenic stage) to stage-III (post-vitellogenic stage) via up-regulation of gene expression and the enzymatic activities of follicle-processing proteases: cathepsin D, chymotrypsin, and carboxypeptidase B1. This is consistent with the finding that CiTKR is expressed exclusively in test cells (functional counterparts of vertebrate granulosa cells) residing in late stage-II follicles (Aoyama et al., 2012). Moreover, Ci Cathepsin D, co-localized with CiTKR in test cells, is initially activated, and Ci Carboxypeptidase B1 and Ci Chymotrypsin, localized in follicular cells, are activated 1 h later (Aoyama et al., 2012). These findings provide evidence for a novel tachykininergic follicle growth pathway. In addition, the CiTK-induced follicle growth is suppressed by a *Ciona*-specific neuropeptide, CiNTLP6, via downregulation of the three aforementioned proteases (Kawada et al., 2011). It would be interesting to reveal whether the tachykininergic regulation of follicle growth is conserved, at least in part, in vertebrates or other invertebrates.

#### Teleost Fish

The roles of NKB in reproductive functions are of interest in both teleosts and mammals. NKB is expressed in the hypothalamus of mammals (Satake and Kawada, 2006; Steinhoff et al., 2014). Moreover, NKB is colocalized with kisspeptin and dynorphin A in KNDy neurons in the arcuate nuclei. KNDy neurons and NKB are responsible for the generation of GnRH pulsatility in the hypothalamus, which plays a central role in reproductive functions via induction of secretion of gonadotropins (LH and FSH) from the pituitary to the gonads (Lehman et al., 2010; Navarro, 2012). Interestingly, some mutations were detected in the genomic sequences of human *Tac3* and *Tacr3* in a portion of patients with hypogonadotrophic hypogonadism (Topaloglu et al., 2009; Chen et al., 2018). NKB is likely to downregulate production of LH and FSH in zebrafish, tilapia and goldfish (Biran et al., 2012; Qi et al., 2015; Hu et al., 2017; Chen et al., 2018; Liu et al., 2019). Several of these studies also indicated that NKB and/or NKF (identical to NKB-related peptide) downregulate the expression of kiss2 that is a homolog of mammalian kisspeptin. The role of mammalian kisspeptin in induction of GnRH synthesis and release may suggest that kisspeptin 2 also upregulates GnRH synthesis and release in teleost. However, conservation of such kisspeptin 2-directed GnRH regulation in teleost is not likely, since kisspeptin 2 seems not to be involved in reproduction in teleosts (Nakajo et al., 2017). Collectively, these findings suggest that biological roles of NKB and NKF in reproduction in teleost are distinct from those in mammals. In addition, SP and NKA were found to upregulate gene expression and release of LH, prolactin, and somatolactin α in carp pituitary cells (Hu et al., 2017). Interestingly, short-term SP treatment (3 h) induces LH release, but long-term SP treatment attenuated gene LH expression (Hu et al., 2017). Thus, in teleosts SP and NKA are important in reproductive functions.

## Conserved Roles of Tachykinin Signaling in the Animal Kingdom

Some of the functional roles of TKs that have been described in some detail in earlier sections are evolutionarily conserved, at least in general terms. By general terms we mean that for instance a role in nociception has been found for TKs both in *Drosophila* (Im et al., 2015) and in mammals [see (Onaga, 2014; Steinhoff et al., 2014; Zieglgänsberger, 2019)], but the neuronal pathways and mechanisms are quite different. In a similar fashion, TKs are acting as cotransmitters in many neuronal circuits and thus play roles in for instance: modulation of olfactory sensory signaling together with GABA in *Drosophila* (Ignell et al., 2009; Ko et al., 2015) and mammals (Olpe et al., 1987), modulation of rhythm generating motor networks in crustaceans (Nusbaum et al., 2017) and lampreys (Parker and Grillner, 1998), aggression in *Drosophila* (Asahina et al., 2014) and mammals (Felipe et al., 1998; Katsouni et al., 2009), as well as roles in learning and memory circuits in honey bees (Takeuchi et al., 2004; Brockmann et al., 2009; Boerjan et al., 2010) and mammals (Lénárd et al., 2018). Furthermore, TKs are involved in regulation of several aspects of intestinal function, including electrolyte and fluid secretion in insects (Johard et al., 2003; Veenstra et al., 2008; Lemaitre and Miguel-Aliaga, 2013; Song et al., 2014) and mammals (Hökfelt et al., 2001; Steinhoff et al., 2014), in regulation of gustatory receptors in *Drosophila* (Shankar et al., 2015) and mammals (Onaga, 2014) and in control of hormone release in insects (Nässel et al., 1995b; Birse et al., 2011; Meschi et al., 2019) and vertebrates (Hu et al., 2014; Steinhoff et al., 2014; Zhang et al., 2019).

Roles of TKs in reproductive functions have been demonstrated in vertebrates (Satake et al., 2013; Steinhoff et al., 2014), but not yet in insects or other invertebrates. However, in insects and other arthropods natalisins seem to be important in reproductive behavior, as outlined in the next section (Jiang et al., 2013; Gui et al., 2018).

## Natalisins, a Sister Group of Tachykinins in Arthropods

A novel peptide precursor gene that encodes multiple copies of peptides that were designated natalisins (NTLs) was discovered in *Drosophila*, *Tribolium castaneum*, and *Bombyx mori*; these have a consensus sequence FXXXRamide (Jiang et al., 2013). The name NTL is derived from the functional role of the peptide in reproduction (Latin word *natalis* for birth) (Jiang et al., 2013). The NTLs have so far only been identified in arthropods and tardigrades, and the peptides display minor similarities to TKs. However, the NTL receptor (NTLR) was previously identified as a TK receptor (CG6115; TakR86C; NKD) (Monnier et al., 1992; Poels et al., 2009), suggesting that NTLs are ancestrally related to arthropod TKs (Jiang et al., 2013). In fact, phylogenetic analysis suggests that NTL signaling arose through duplication of the TK signaling early in the arthropod lineage (Jiang et al., 2013; see Figure 6). However, TK-like precursors with NTL-like peptides are found in the spider mite (chelicerate) as well as tardigrades as shown in Figures 4, 5, suggesting that the NTL signaling might also be present outside arthropods (Veenstra et al., 2012; Koziol, 2018). It was noted that in the centipede *Strigamia maritima* (Myriapoda) there is no NTL gene (Veenstra, 2016) and in the spider mite *Tetranychus urticae*, there is no separate NTL gene in the genome (Veenstra et al., 2012). However, two TK genes were annotated in *T. urticae* and on these precursors two of the three putative mature peptides are similar to NTL, and one is a TK; the second precursor encodes two TKs and an unrelated peptide, but no NTL (Figure 4). Thus, with this mix of TKs and NTLs on the spider mite genes, it was suggested that TK and NTL divergence started by internal events on duplicated TK genes and resulted in the evolution of a separate NTL signaling system (Jiang et al., 2013). Additional genomes/transcriptomes of basal arthropods need to be examined to substantiate this claim. As seen in Supplementary Table S5, there are five paracopies of natalisins in *Drosophila*, 7 in *Anopheles aegypti*, 11 in *Bombyx mori* and 15 in *Manduca sexta*, 2 in *Tibolium castaneum* and only one each in the tardigrades *Hypsibius dujardini* and *Ramazzottius varieornatus* (Jiang et al., 2013; Koziol, 2018). The *Drosophila* peptides (DmNTL1-5) range from 15 to 24 residues and have a consensus C-terminus FXPXRamide (except DmNTL4).

In the brains of *Drosophila, B. mori, T. castaneum* and *Varroa destructor*, there are two pairs of identifiable NTL neurons with very similar locations and arborizations (designated ADLI and ICLI in each species) (Jiang et al., 2013, 2016). The *Drosophila* neurons are shown in Supplementary Figure S6. In the *B. mori* brain, there are two additional pairs of neurons in the subesophageal zone. Another study shows that in the oriental fruit fly *Bactrocera dorsalis* there are three pairs of NTL neurons (Gui et al., 2017a). Thus, in insects studied so far, the NTL system appears relatively simple and the neuronal branches do not seem to innervate any of the well-defined centers, such as antennal lobes, mushroom bodies, central complex or optic lobes (Jiang et al., 2013). There are a few additional segmental neurons in the ventral nerve cord. The brain ICLI neurons coexpress NTL, Ast-A and MIP (Diesner et al., 2018).

In *Drosophila*, genetic experiments revealed that NTL and the four NTL neurons are important for male mating success (Jiang et al., 2013). NTL-RNAi in NTL-Gal4 neurons reduces male copulation success rate. The courtship behavior was only affected in the latency of courtship initiation in males. NTL-RNAi females also displayed reduced mating frequency, but did not actively reject males (Jiang et al., 2013). Silencing of the NTL-Gal4 neurons resulted in complete repression of mating in males, but had no effect in females. Manipulations of NTL neurons had no effect on egg laying, however, in *T. castaneum* systemic NTL-RNAi in either sex resulted in reduced egg numbers after mating (Jiang et al., 2013). Also in the oriental fruit fly *Bactrocera dorsalis* NTL and its receptor (NTLR) play important roles in mating (Gui et al., 2017a, 2018). In this species the NTL signaling is required for regulation of mating frequency in both males and females.

## Conclusion and Perspectives

We have shown that TKs are neuropeptides that emerged early in bilaterian lineages, but it is not clear what their ancestral form is since cnidarians and other non-bilaterian do not possess typical TKs, although bioinformatics has indicated presence of TK receptors [see (Krishnan and Schiöth, 2015; Hayakawa et al., 2019)]. It is also puzzling that no typical TKs have been identified in echinoderms, acorn worms, or amphioxus. Possibly this is due to species-specific diversification of TK sequences. Thus, important questions regarding the evolution and diversification of this signaling system remain unanswered. Nevertheless, it is clear that TK signaling is widespread and diverse among bilaterians and contributes to many vital functions. Some of these functions appear conserved over evolution, at least in general terms. It is important to stress that TKs seem to have multiple distributed (localized) functions in different neuronal circuits and commonly act as co-transmitters, and thus TK signaling is not likely to orchestrate global functions. Elucidation of TK functions in neglected phyla (such as echinoderms, xenacoelomorphs and cnidarians) can also provide clues on whether the mode of action of TK as a co-transmitter is an ancient or a more derived trait. This is important since besides TK, there are only a few other neuropeptides in protostomes (at least in arthropods) that seem to function mainly as co-transmitters, such as sNPF and proctolin (Nässel, 2018; Nässel and Zandawala, 2019). Did TKs evolve as primary paracrine peptide signals in basal phyla with simple nervous systems, and then diversified functionally to also confer plasticity to more complex neural circuits by providing neuromodulatory actions as co-transmitters? In organisms without nervous systems, such a *Trichoplax adherens*, peptides seem to act as primary messengers that induce simple behaviors (Nikitin, 2015; Senatore et al., 2017; Varoqueaux et al., 2018) and even in more evolved organisms many neuropeptides/peptide hormones seem to relay single global orchestrating actions [see (Nässel and Zandawala, 2019; Nässel et al., 2019)].

In mammals, TK signaling has received extensive attention due to its clinical importance with roles for instance in pain, inflammation, cancer, depressive disorder and immune system. Thus, the literature list is huge: searching PubMed for the term “substance P” renders more than 24,000 hits. This means that our coverage of mammalian TKs in this review is very superficial and incomplete. On the other hand, a search for e.g., “tachykinin in insects” yields about 200 hits and therefore our discussion of invertebrate TKs is somewhat more detailed, but certainly still providing a sketchy picture of TK signaling since functional studies are not yet that numerous.

The development of powerful genetic tools, not only in *Drosophila* and *C. elegans*, but also other organisms has improved the possibilities to analyze neuropeptide signaling down to single identified neurons or sets of neurons. Furthermore, with optogenetics and other strategies for temporal control of manipulations and elegant techniques for imaging neuronal connections or activity, we already see an increase in studies of invertebrate neuropeptides. For TKs, it is of importance to note that in the CNS these peptides seem to operate as local neuromodulators and/or co-transmitters. Many (if not most) TK expressing neurons may additionally signal with small molecule transmitters and, therefore, manipulations of TK signaling only remove one layer of the signal transfer.

Whereas many neuropeptides also have functions as circulating hormones [see (Nässel and Zandawala, 2019)], it seems like TKs do not in most organisms studied. In studies of cockroach, locust and *Drosophila* it was proposed that TKs are released into the circulation from gut endocrine cells to stimulate secretion in nearby Malpighian tubules (Winther and Nässel, 2001; Johard et al., 2003; Söderberg et al., 2011). A recent *Drosophila* study showed that gut TKs act locally and do not affect behavior, indicating that there is no signaling to the brain via the circulation (Song et al., 2014). If *bona fide* hormonal roles of TKs can be excluded, we can focus on their local actions, but we still face some difficulties due to the diversity of TK expressing neuronal systems and the co-expression of small molecule transmitters. Hopefully this review will trigger interest in TK signaling in invertebrates in spite of these challenges. It is obvious from the literature that research on TK signaling in mammals is already very extensive, but certainly further basic research and clinical studies are urgently needed to unravel this important and interesting signaling system.

## Author Contributions

DN contributed to the conceptualization, prepared the first draft of the manuscript, wrote parts of the manuscript, prepared figures and tables, and coordinated the assembly of the manuscript. MZ contributed to the conceptualization, wrote parts of the manuscript, and prepared figures and tables. TK and HS wrote parts of the manuscript, and prepared figures and tables. All authors edited and finally approved the manuscript.

## Conflict of Interest

The authors declare that the research was conducted in the absence of any commercial or financial relationships that could be construed as a potential conflict of interest.

## Footnotes

1 http://www.neurostresspep.eu/diner/insectneuropeptides

2 http://ghost.zool.kyoto-u.ac.jp/SearchGenomekh.html
