# Supplementary material for: Tachykinins: Neuropeptides That Are Ancient, Diverse, Widespread and Functionally Pleiotropic
Source: Front Neurosci. 2019 Nov 20;13:1262. doi: 10.3389/fnins.2019.01262 (PMC6880623; doi:10.3389/fnins.2019.01262)
Supplement: Supplementary file 1 [file Data_Sheet_1.PDF]

Supplemantary Text Fil 1

Sequences of TK receptors used in phylogenetic tree in Fig. 5

>Anoga_NTLR
MSESVELELLINCTQATILRFPWSTDFNTTVIGELNRTEVLDVLGEMITRSRG
YVPKEFITECLFPTTKSPYELPWQLKTAWGVVFGAMLLVAITGNCIVLWIVLA
HRRMRTVTNYFLLNLSVADLLMSSLNCSFNFIFMLNSDWPFGSVYCTINNFM
ANMSVASSVFTLVAISFDRYIAIVHPLRHRTSRKKARIFLLIIWALSCVLAAPCL
MYSTVMTKRYNNGKTRTVCYMLWPDGRYPTSMADYIYNLVFLILTYGIPMLI
MIVCYSLMGRELWGSRSIGEHTERQLESMKSKKKVSVVRMFIIVVTIFAICWL
PYHLFFVYAYHNNQMTSSSYVQQLYLGFYWLAMSNAMVNPIIYYWMNGRF
RVYFQEIICFCCLQFINSRMRDGVPQGVLLNKNSNSELGRYRSYRPRSVSV
RWRPPSTEEYRMRTGLPRPAHLGVNVLESAANGGSDKQPVVDIAELQTINF
LHLNHNSNELQHSSSNSHEFS
>Anoga_TKR
MDLEELAVTLLTGGNKSTAGTSSCCPAGTGLNGSGTEPGWSATSAELEIAW
RESSPPTLVAGPYPEPNGTAESMYQTMDFFSILPLWRLIVWNVLFAGIVITAT
VGNLIVVWIVLSHKRMRTVTNYFLGADAMVSTLNVTFNYTYMLYLDWPFGT
MYCKISQFVAILSICASVFTLMAIAIDMNPLKPRMGKKATLCVAASIWIVGTIIS
CPSLLFFTTYPMKDHILCYAEWPDGPSNHSRQEYYNIVFMLLTYFLPIGSMT
YTYARVGLELWGSKSIGECTQRQLDNIKSKRRVVKMMMIVVIIFAVCWLPFQI
YFILTSYYPELTKKPYIQEVYLAIYWLAMSNSMYNPIIYCWMNLRFRRGFQQF
FRCCPFVRVTPDSASSHRRTGTERSFLYNGSQSPTGQRKWQTGPMRRVN
TMLTSQMLNQTTSLCCASPADSNPPGGPGRPKD
>Astru_LuqR1
MSGLTISNFSETINSSDSSDYDYPTYEYYEEPVWIQAIFIFLYGTVSVIGIFGN
GIVCYIVLGHPRMRTVTNYFIVNLAIGDLLMAAMCVNFTVYATLYNQWPFGE
LMCKLVSFSQTISVSVSIYTLVAIGVDRYFAIIHPLRPRMGSKETLFVIGVIWVI
SIALALPAALFTTLESQGGSTFCTDGAWEDSLVYSLICMVLQYFLPLAVLMGA
YIRIGKRIWGRRTPGEVEAERDKKMSESKARLVKMFATIVLLFALCYMPIQIY
TIMQDSNRSILSFYYIKIVYLCCLLSAMSNCVYNPFIYCWMNIKFRNGFRSVF
RFLPCVNYQGDWNGFTGLRRANTAQTQTETMSMGSRNDRGRWWTQDQA
KIAKNGRASNSDGETRTSFM
>Astru_LuqR2
MNISYSNDSSGGYSDVCVEYYREPVWLQVLFGIIFGLITVFGIGGNAIVCYIVL
GHRRMRTVTNYFVVNLAVSDQLMAVMCVNFTFYSTLYMTWPFGPVMCKAV
SFFQSVSVSVSIFTLVAISMERYMAIIHPLRPRLGSTGTLLVIGFIWISSGALGL
PTAIYTSVYVENGITYCSEEWDQRGNYSFATMVLQYFLPLAVLAMAYSRIGIR
IWARKTPGEMEANRDRRMTESKIRLVKMFAVVVFLFAICYLPIHTFNIVQDVY
QYVLCYQYIRIIYVTVVAVAMSNCMYNPFIYCWMNSKFRDGFKNVVRCLPGV
RRYRDRQQANQSGSFRGLKRTSTLNTHMQS
>Astru_TKR1
MVDYKDINSLNFTFVHNLDQESRILWSVFCSIIMAVAAGGNLIVIWIVATNPR
MRTVTNYFLLNLAVADALIATLSMPFMFSYIVTQNWALGIGMCKVVRFFGLV
STSASVLSLVAISIDRYRAIVHPLLPRLSKSYIVCMIIFIWGGSSIFASPLLVYST
VLTFMYNDGVIKNQCFIKWPDGIYRRIDFTYNVASFAVLYCLPLTVLAGCYTVI
GVKLWRGDVLGEYIPNRARQLKAKRKVVKMIIVVIAVFAICWFPLHVYQFLGY
LHEEVYTQSYAVHIYSTIWTLAMSSSMYNPFIYCWLNDRFRAGFKRVFHCLI
PGSKKQRDNKGIDRRTPLTSTTSNANSLMTASTTGMHRQVPNGKPAILCRT
SLIGISVVDSSEDIF

>Astru_TKR2
MGNKTSDFMEYEDFYTINNTFPHVMDQQSRIIWSVFVSAIILTAAGGNLIVIWI
VSTDPRMRSITNYFLLNLAVSDTLIATLSMPSLFSYIVTQNWVMGEFMCRLA
RFMGTVSTVASVLSLVAISIDRYRAIVHPLLPRLSKTCIVCMIIFIWIGAISFASP
QFFYSQLITFGYSDGDITQCYIIWPDGIQREYEFWYNVTSFSVVYCVPLSVLA
MCYTVIGVKLWRSGVLGEYIPNRAKHIKAKRKVVKMIIVVIAVFATCWLPLHIY
QFLSFMSDSAYQKSYSLHIYLSVWTLAMSSSMYNPFIYCWLNDRFRAGFKR
VFRCSQGTSDTRHSQRQSGTPMSPTSRMTSSTGYQRTTNGKQELGCKNS
LLSASVVDSMEDTC
>Bacdo_NTLR
MSDIVDTELLVNCTILAVRRFGLNTIVNSSLLSSLNRSEVINLLSGIIENKDNLD
NINDAKDFLTECLFPSPTRPYELPWEQKTIWAIIFGMMLFVAIAGNCIVLWIVA
GHRSMRTVTNYFLLNLSIADLLMSSLNCVFNFIFMLNSDWPFGSIYCTINNFM
ANVTVSTSVFTLVAISLDRYFAIVHPLKRRTSRRKVRIILLLIWVISCLLAAPCLL
YSSTMTKRYYNGKSRTVCFMLWPDGRYPTSISDYAYNIIILILTYGIPMVVMLI
CYTLMGRVLWGSRSIGENIDRQMESMKSKRKVVRMFIAIVLIFAICWLPYHM
FFIYAYHNNDITSAKYVQHMYLGFYWLAMSNAMVNPIIYYWMNKRFRLYFQ
RIICCCCYGAVNHKTDSPNLIANKNSFQKSIKVEGKNIWKRSTMETQIQVAQT
SLRERITMDKTPNKVIVDCIQEKAKNDSSPVYISTKSGTDQQRIIIKCISCDED
NKYGKDIQGKNGKDGKDTL
>Bacdo_TKR
MIDPHMSPTTAVSNFGSIEPYKVDTFNEFESSGGGGGRGELDVIIASETSSA
AFGGLAENLQDNYSQLISTTSAIDWCNQSLLLYGINNCSINIYRGINDTMDDE
DISFILPWWRQVLWSILFGGMVLVATGGNLIVVWVVMTTKRMRTVTNYFIVN
LSIADAMVSSLNVTFNYIYMLDNDWPFGELYCKISQFIATLSISASVFTLMAISI
DRYVAIMKPLKPRMSKRCNLGIAAFIWIASIAISCPMLFFFTTGEVELKDGTRI
VCYAEWPDGPTNHSQQENVYNIVFMILTYILPIISMTVTYSRVGIELWGSKTIG
EYTPRQVENVRSKRRVVKMMMVVVLIFAVCWLPFHIYFIVTSCYPALTQTPFI
QEVYLGIYWLAMSNSMYNPIIYCWMNSRFRYGFKRFFRWCPFVKVDESLNR
RDNMTSRYSCSGSPDHNRIKRNDTQRSFLYTCPSSPKNRRVASHYGMLRN
STIVSHRDPMRHSAPIGTNNTGAYHQRFTISHCGNSHELTSLTSLSGAPGAG
QYGIVMKAEPIKVALRNGSAQQVSLPLWPATVETTDTESSTTTPPLLPPPST
LGVGEVVGVEGCRVGDADADGGVCRVDETNVRAAS
>Bommo_NTLR(A32)
MPPWAHNSWICVFSIMLIIAVGGNAIVIWIVIAHKRMRTVTNYFLVNLSLADLM
MSALNCLFNFIYMLHSDWVFGLQYCKISNFIANVTVAASVFTLTGISFDRFQAI
VRPMRPRMSKTCSLIAIGGIWLGGMVLATPYLLYSTTKEYKSRVGVKTACLL
VWPDGMPDVSKMDFVYQIAFFIVTYAVPMVGMSFFYTAMGRELWGSRTIG
ELTQRQLDSIKSKRKVVKMFILVIVIFGICWFPYHGYFIYTHLDSSILYSRYVQH
VYLGFYWLAMSNAMVNPIIYYWMNAKFRSYFRMAIMCRWLEVMWRRRHPL
DSPPECPSQSNTRSRSGFYSLTYRGMQRIKRKYSSRMGAPHASDPQRPAL
AETVFAC
>Bommo_NTLR(A33)
MDSDLDIQSFINCTQQIFGHEQKWDDLNVSEILDLLPKQILEDINLKITLGNCM
GLGERPYSPPWWGQLAWFIVFAVMLLLAVIGNTMVIWIVLAHRRMRTVTNC
FLVNLAVADLLMATLNGAPNFVFLVTANWPFGAVTCTASNFTASLTVSAGVF
TLVAITVDRYVAIVKPLQHRLSRRVVRAALFTVWIASAMLALPSLLYSDTYKK
QYVNGEREICFIKWPDGSYPTSLSDYCYNLVFLSVTYVLPMAVMVWAYAQM
SAALTGRAIGECTLHQMQVVRAKRKVVRMFVLVVMVFALCWLPYHAYFVLV

YHHQSLATAPFAQHIYLGFYWLAMANSMFNPLIYYWMSNKFRLYFRLVLCW
CWKSESATPNDLKKLEVKSYSVSQRHFRDVSSFSRA
>Bommo_TKR(A24)
MMLDELGPTVASNQSTSLADLDSFYVTFYDVENERYVNDSQNATEPFQSFI
LPWWRQILWTVLFAGMVVVATVGNLVVIWIVLTNKRMRSVTNYFLVNLSVA
DAMVSTLNVTFNFTYMLNSNWPFGHFYCKFCQFIAVLSISASVFTLLAISVDR
YVAIMSPLQPRLGKRATLGITAAIWAWSSFISSPNLIYFTTENVSLPDGTIRCV
CYSHWPDGMTTRSRLEYAYNVLFMVLTYFMPIIAMTYAYSRVGVELWGSQS
IGECTQRQLDNVKSKRRVVKMMIVVVVIFAVCWLPFHVYFVVTSYYPDVVSY
PHIQEIYLGIYWLAMSNSMYNPIIYCWMNSKFRRGFKQFFWCCGAFGGGGL
ARHRALGPDRTDRSMRSLSPSRKNGTSM
>Braflo_TKR
MDNFINESTHVVENSTGEPFSATNQFKLPEWQVALWSFPYTAIVLAAIVGNG
IVIWVVLAHANMRTVTNYFLVNLAFADVSIASFNTIFTFTFSVTGDWYYGKFY
CYFMNFVPTTAVSASIYTLTAICLERYIAIVHPLKPRMSRVCAKSVIIAIWLCAA
TVNVPLCIYSHFYTFKYREEEWSVCRFQWPDDNVKFWYDVGLMTINYFMPL
LVMAVAYSIVGFTLWAGKIPGEATQRHLEQMKAKRKVVKMMVIVVVTFCVC
WLPYHVFFLLPPGYKEWKHIQQVYLAVFWLAMSNSMYNPFIYCWLNKRFRY
GFQTALRWCPGVPAPKDPDSSLKMNRFTKSTSTQISTVRLHTIHKSVSAGS
RIARRVSETSVV
>Capte_LuqR
MPAYAQAIIIVMYSTISVLAVGGNLIVVYIVVAYQRMRTVTNYFIVNLACSDILM
AVMCIPFTFIANLLMHYWPFGAIMCPIVSYLQAVVVFLSAFTLVAISLDRYRAI
MFPLKPRMTTNQVAMVIAVIWMLSLAVPLPIAIMSRIEEQTDRFNASVVRYIC
KEKWPQLQHRYIYSLCLMILQYFLPLFVLMFTYSSIAVVIWVKKMPGEAENN
RDQRMAASKRKMVKMMITVVIIYALCWLPLHTVTIVGDVHESVWHFRHIQVL
WSGAHWLAMSNCCYNPIVYCWMNSKFRQGFKYSLRCQKLLSRRKMTH
>Capte_TKR
MNEHGSNPYIMHWYITLLYIIIFVVMVLVASGGNIIVIWIVLAHKRMRTVTNYFL
VNLAVADLMITILNTLFNFVYMLYSAWPFGEIYCKFTNFISICTISASVFTFMAI
AIDRYMAIIHPLKPRIGAATILGLIVIIWVTSIAVAMPTLLVAKTSTITYTATGDRT
VCYLEWPDGVGETSDLCYNIFLMLVTYFIPLLILGVTYLKVGVSLWGSQAIGE
RIARQEETVRSKRRVVKMMIVVVLIFGVCWLPQNVLILLSLTIKGLVDITYLQHI
YMIAYWIAMSNSMYNPIIYCWMNQRFRKGFKYVFQWLPFVHWRPSDAKGL
GRGGVSTSVRMSVSENTKYTEANDGTSCSVMQTMIERLEESRCCSPQGGR
NDAHKLYPHGKSGTEDEAL
>Cioin_TKR
MNISSTHDPSGRMVTDVTATTEDHHEDEENPFAQSPYAIFGWSVVYGLLVV
VALVGNLGICWIVIRNKRMQTVTNFFLASTAFADSNVIGFNTVFNFTYALNND
WYFGKAFCHFINFVPIGAVLASILSITVISLDRFVVIMYPLRRRTSRKTAKMTIA
GIWLFSLGVAFPQCFFATITTEESGTRTTCSIQWPDGVSGRMRLGYQLSFM
VISYFLPLIILAVSYVAMALRLCGSNNQVGHQNETQLRRIANNKKAVRMMML
VVVVFAICWCPYHLFFLADYIVSDSYHWEKIQQVYLAVFWVAMSSSMYNPFI
YCWNNSRFKESFRELFHCGGARGHRSFAFRLRQRSRGA
>Cragi_TKR
MEGNNSTKAGPSEMFIPMPVWQQILFITMYVVMIVVAAGGNLAVIWIVMAHK
RMRTFTNYFLVNLAVADTLISLFNTAFVSTFLIYQDWWYGEIYCKFSNFINVS
TLAASVLTFMSIAIDRYLAIIHPLRPRLTVRVVLAIVVIWQVSIVLAIPNLIYGKTE
KYHDRVICLLDWSQELDFGYNLLILIVTYVLPLVTLTVTYARVGFELWGSRAI
GENTPVQYERIRSKRRVVKMMIVVVVTFAVCWLPYHLYFVLAATKPEINNWK

HMQNVYNVIYWMAMSNSMYNPIIYCWMNSRFRHGFVKLFCCCPCRLCAKF
KDRSKPGRLPVTLFRNSSNTYALTDKHHGNGCSQYTTTESVDDASSSPVNH
KKGRRTSAEYL
>Danre_Tacr1a
MDSFITSTDFPSNWTVNSTAQNETEIYWNQFVQPVWRIVLWAVAYSTIVVVS
VVGNITVIWIILAHKRMRTVTNYFLVNLAFAEASMSAFNTVINFVYSVHNEWY
FGLYYCRFHNFFPIAAVFASIYSMTAIALDRYMAIIHPLQQRMSATQTKVVIGV
IWILALLLAFPQYYYSDTDQLPGRVVCYIDWPEYTLLDFKTMYFVCVAVLIYFL
PLLVMGCAYLVVGLTLWASEIPGDSSDRYQEQLTAKRKVVKMMIVVVCTFA
VCWLPYHVYFLIHQFYPHLFEHTFIQQVYLTIMWLAMSSTMYNPIIYCCLNDR
FRAGFKQAFRCCPCVPEGSYEGLELKSTRYLQTQTSLYRASRMETSVSCV
MQPSEDGHKVTFGSVRGAAVKPAARSPSREFTSNGSSSRSISKTVSETSSF
YSSNNLQE
>Danre_Tacr1b
MDPLYITSPNSSADNGSAGSPVNDTEVFGNQFVQPVWQIVLWAIAYCTIVLV
SVVGNITVIWIILAHKRMRTVTNYFLVNLAFAEASMSAFNTVINFIYAVHNEWY
FGLVYCRFHNFFPIAAVFASIYSMTAIALDRYMAIIHPLQQRLSSAETKLVVCVI
WALALMLAFPQYYFSSTAQLPGRVVCYINWPEYSMWDFQKTYYVCVVVLIY
FLPLLVMGCAYLVVGCFLWASEIPGDSSDRYREQLIAKRKVVKMMIVVVCTF
ATCWLPYHVYFIVHQFLPHLFEERYIQQVYLGIMWLAMSSTMYNPIIYCLLND
RFRAGFQQAFSWCPCVPQGSYEGLELKSTRYLQTQASIFRASRMETTVSTV
MPTGDGELPENPNRSSVDLTSNGSSRSASKTVSESSFYSSNNVA
>Danre_Tacr2
MDTTLDPLSSSLLYYDEDGNETSINLFEQPDWQVALWAIAYTLIVIISFIGNVT
VIWIILAHKRMRTVTNYFIVNLAFSDASMATFNTVFNFVYALHNDWYFGLGYC
KFQNFFPITAMFSSIYSMAAIAVDRYMAIIHPLKPRLSSTTTKLLIGVIWTVAFS
LAFPQCYYASTKFYFPRTVCMVEWPDDYGGKHQLSYQIAVIILIYLLPLLVML
VTYSLVGQRLWGSEIPGEASDHYQNQMQAKRKVVKMMIVVVTTFAICWLPY
HIYFILGSFNRDIYKQHYIQQVYLAIFWLAMSSTMYNPIIYCCLNQRFRSGFRK
AFQWCPFVKISEEDNMELQHMRTFHMRRSYRTETTSVVVRNHANEPEDTT
SKLIKA
>Danre_Tacr3a
MAQSQNGSNLTGNFTNQFVQPPWRVALWSVAYSSILAIAVFGNLIVMWIILA
HKRMRTVTNYFLLNLAFSDASMAAFNTLINFVYATHGDWYFGEAYCKFHNF
FPVTSVFASIYSMSAIAVDRYMAIIHPLKPRLSATATKVIIVCIWVLAVVLAFPL
CFFSTIKKLPKRTLCYVAWPRPSEDPFMYHIIVAMLVYVLPLVVMGINYTIVGL
TLWGGEIPGDSSDNYQGQLRAKRKVVKMMIIVVVTFAFCWLPYHVYFLVTG
LNKQLARWKFIQQIYLSIMWLAMSSTMYNPIIYCCLNSRFRAGFKRVFRWCP
FVQVSDYDELELRAMRHKVARQSSMYTMSRMETTVVTVCDPSEPNTQPGR
KSLLNHHHHHNGCSNPAKSKEITYMQSDPKEEFS
>Danre_Tacr3b
MAGPQSGSNVTRNFTNQFVQPPWRVAVWSVAYSSVLAVAVFGNLIVIWIILA
HKRMRTVTNYFLLNLAFSDASMAAFNTLINFIYATHGEWYFGEVYCKFHNFF
PVTAVFASIYSMTAIAVDRYMAIIHPLKPRLSATATKVVIVCIWALAVILAFPLC
FYSTTRTMPRRTICYVAWPRPAEDSFMYHIIVTVLVYMLPLVVMGITYTIVGV
TLWGGEIPGDSSDNYVGQLRAKRKVVKMMIVVVVTFALCWLPYHIYFIVTGL
NKRLNKWKSIQQVYLSVLWLAMSSTMYNPIIYCCLNGRFRAGFKRAFRWCP
FIQVSSYDELELRPTRLHPRNQSSMCTLSRVDTSLHGEDPRRSQRKSTKSQ
CLVEVRDENTPATKLCLNRDQAFATEQLS
>Danre_Tacr3c

MSSPRNSSNFTHINRFVQPPWRVALWSVAFALVLLVAVTGNLIVIWIIVAHKR
MRTVTNYFLLNLAVSDVCVAALNALVNFVYGAHGHWYFSSAYCRFQNFYPV
AAVFASIYSMSAIALDRYMAIIHPMKPRLSAKVTKAVIVCVWILAAFLSFPLCF
YSVTEVLPHRTVCYVAWPRKDDDAFIYHVVVALLVYLLPLALMAVTYSRVGL
TLWGGDFPGHSSENLLDHLQAKRKVVKMMVIVVVTFAICWLPYHVYFIVTSF
NRALRRFKWIQQVYLSVLWLSMSSSMYNPIIYCCLNSRFRAGFKRVFRWCP
FIQMSNCDELELQTARFQQQRQSSVFTVTRMESDGSAAVSRRKSSSTSRC
SVRSQSRPSARPPHNGAPHGNISCVPETLS
>Drome_NTLR
MSEIVDTELLVNCTILAVRRFELNSIVNTTLLGSLNRTEVVSLLSSIIDNRDNLE
SINEAKDFLTECLFPSPTRPYELPWEQKTIWAIIFGLMMFVAIAGNGIVLWIVT
GHRSMRTVTNYFLLNLSIADLLMSSLNCVFNFIFMLNSDWPFGSIYCTINNFV
ANVTVSTSVFTLVAISFDRYIAIVDPLKRRTSRRKVRIILVLIWALSCVLSAPCL
LYSSIMTKQYYNGKSRTVCFMMWPDGRYPTSMADYAYNLIILVLTTGIPMIV
MLICYSLMGRVPGGSRSIGENTDRQMESMKSKRKVVRMFIAIVSIFAICWLP
YHLFFIYAYHNNQVASTKYVQHMYLGFYWLAMSNAMVNPLIYYWMNKRFR
MYFQRIICCCCVGLTRHRFDSPKSRLTNKNSSNRHTRAETKSQWKRSTMET
QIQQAPVTSSCREQRSAQQQQPPGSGTNRAAVECIMERPADGSSSPLCLSI
NNSIGERQRVKIKYISCDEDNNPVELSPKQM
>Drome_RYaR
MEHHNSHLLPGGSEKMYYIAHQQPMLRNEDDNYQEGYFIRPDPASLIYNTT
ALPADDEGSNYGYGSTTTLSGLQFETYNITVMMNFSCDDYDLLSEDMWSSA
YFKIIVYMLYIPIFIFALIGNGTVCYIVYSTPRMRTVTNYFIASLAIGDILMSFFCV
PSSFISLFILNYWPFGLALCHFVNYSQAVSVLVSAYTLVAISIDRYIAIMWPLKP
RITKRYATFIIAGVWFIALATALPIPIVSGLDIPMSPWHTKCEKYICREMWPSR
TQEYYYTLSLFALQFVVPLGVLIFTYARITIRVWAKRPPGEAETNRDQRMAR
SKRKMVKMMLTVVIVFTCCWLPFNILQLLLNDEEFAHWDPLPYVWFAFHWL
AMSHCCYNPIIYCYMNARFRSGFVQLMHRMPGLRRWCCLRSVGDRMNAT
SGTGPALPLNRMNTSTTYISARRKPRATSLRANPLSCGETSPLR
>Drome_TKR
MENRSDFEADDYGDISWSNWSNWSTPAGVLFSAMSSVLSASNHTPCRTLA
RSSPYPPVSFNHSQTLSTDQPAVGDVEDAAEDAAASMETGSFAFVVPWWR
QVLWSILFGGMVIVATGGNLIVVWIVMTTKRMRTVTNYFIVNLSIADAMVSSL
NVTFNYYYMLDSDWPFGEFYCKLSQFIAMLSICASVFTLMAISIDRYVAIIRPL
QPRMSKRCNLAIAAVIWLASTLISCPMMIIYRTEEVPVRGLSNRTVCYPEWP
DGPTNHSTMESLYNILIIILTYFLPIVSMTVTYSRVGIELWGSKTIGECTPRQVE
NVRSKRRVVKMMIVVVLIFAICWLPFHSYFIITSCYPAITEAPFIQELYLAIYWL
AMSNSMYNPIIYCWMNSRFRYGFKMVFRWCLFVRVGTEPFSRRENLTSRY
SCSGSPDHNRIKRNDTQKSILYTCPSSPKSHRISHSGTGRSATLRNSLPAES
LSSGGSGGGGHRKRLSYQQEMQQRWSGPNSATAVTNSSSTANTTQLLS
>Galga_NK1R
MDDPPPLEAELEHRWLLNASLNESSANQFVQPPWQVALWAVAYTLIVVVSV
VGNVVVMWIILAHKRMRTVTNYFLVNLAFAEASMSAFNTVVNFTYAIHNEWY
YGLLYCKFHNFFPIAAVFASIYSMTAIALDRYMAIIHPLQPRLSATATKVVICVI
WLLAFLLAFPQGYYSVTEELPGRLVCLVAWPEHSTDVYGKTYHFCMTVLIYF
LPLLVIGCAYTVVSITLWASEIPGDSSDRYHEQVSAKRKVVKMMIIVVCTFAL
CWLPYHIYFTLQYFNPEWYLQKFIQQVYLAVMWLAMSSTMYNPIIYCCLNDR
FRVGFKHAFRWCPFVSAAEYEGLEMKSARYLQTQSSMYKVSRIETTVSLAV
GAAEEELEESKKGKRLSVDMTSNGSSRSDSKTVSESFSFYSNTLT
>Galga_NK3R

MNSVSAANWNVSMSSRWNMSLAAAGDPWGQALGVPAGNASTPVAANFS
NQFVQPSWRIALWSLAYGGVVAVAIFGNLIVIWIILAHKRMRTVTNYFLVNLA
FSDASMAAFNTLINFIYALHSEWYFGEAYCRFHNFFPITAVFASIYSMTAIAVD
RYMAIIDPLKPRLSATATKVVIGSIWILAFLLAFPQCLYSITKVMPGRTLCYVA
WPGGPKQHFTYHVIVIVLVYCFPLLVMGITYSVVGITLWGGEIPGDTSDKYHE
QLKAKRKVVKMMIVVVMTFAICWLPYHTYFIVTGIYQQLNRWKYIQQIYLASF
WLAMSSTMYNPIIYCCLNRRFRAGFKRAFRWCPFIELSSHDELELKAARFHP
TRQSSLYAVSRMESSTTVVLDTNDGDNSHPSRKKKAAPRNASFNGCSQRN
SKAVSTASSFVSSLNTAGDDYS
>Homsa_NK1R
MDNVLPVDSDLSPNISTNTSEPNQFVQPAWQIVLWAAAYTVIVVTSVVGNVV
VMWIILAHKRMRTVTNYFLVNLAFAEASMAAFNTVVNFTYAVHNEWYYGLF
YCKFHNFFPIAAVFASIYSMTAVAFDRYMAIIHPLQPRLSATATKVVICVIWVL
ALLLAFPQGYYSTTETMPSRVVCMIEWPEHPNKIYEKVYHICVTVLIYFLPLLV
IGYAYTVVGITLWASEIPGDSSDRYHEQVSAKRKVVKMMIVVVCTFAICWLP
FHIFFLLPYINPDLYLKKFIQQVYLAIMWLAMSSTMYNPIIYCCLNDRFRLGFK
HAFRCCPFISAGDYEGLEMKSTRYLQTQGSVYKVSRLETTISTVVGAHEEEP
EDGPKATPSSLDLTSNCSSRSDSKTMTESFSFSSNVLS
>Homsa_NK2R
MGTCDIVTEANISSGPESNTTGITAFSMPSWQLALWATAYLALVLVAVTGNAI
VIWIILAHRRMRTVTNYFIVNLALADLCMAAFNAAFNFVYASHNIWYFGRAFC
YFQNLFPITAMFVSIYSMTAIAADRYMAIVHPFQPRLSAPSTKAVIAGIWLVAL
ALASPQCFYSTVTMDQGATKCVVAWPEDSGGKTLLLYHLVVIALIYFLPLAV
MFVAYSVIGLTLWRRAVPGHQAHGANLRHLQAKKKFVKTMVLVVLTFAICW
LPYHLYFILGSFQEDIYCHKFIQQVYLALFWLAMSSTMYNPIIYCCLNHRFRS
GFRLAFRCCPWVTPTKEDKLELTPTTSLSTRVNRCHTKETLFMAGDTAPSE
ATSGEAGRPQDGSGLWFGYGLLAPTKTHVEI
>Homsa_NK3R
MATLPAAETWIDGGGGVGADAVNLTASLAAGAATGAVETGWLQLLDQAGN
LSSSPSALGLPVASPAPSQPWANLTNQFVQPSWRIALWSLAYGVVVAVAVL
GNLIVIWIILAHKRMRTVTNYFLVNLAFSDASMAAFNTLVNFIYALHSEWYFG
ANYCRFQNFFPITAVFASIYSMTAIAVDRYMAIIDPLKPRLSATATKIVIGSIWIL
AFLLAFPQCLYSKTKVMPGRTLCFVQWPEGPKQHFTYHIIVIILVYCFPLLIMG
ITYTIVGITLWGGEIPGDTCDKYHEQLKAKRKVVKMMIIVVMTFAICWLPYHIY
FILTAIYQQLNRWKYIQQVYLASFWLAMSSTMYNPIIYCCLNKRFRAGFKRAF
RWCPFIKVSSYDELELKTTRFHPNRQSSMYTVTRMESMTVVFDPNDADTTR
SSRKKRATPRDPSFNGCSRRNSKSASATSSFISSPYTSVDEYS
>Hypdu_TKR
MSATTLNYSTLDYSTMTLNGVEFFNGTTVSPEGSYNITCEGYGSAVPNGTK
RDFSLPLPSQVFYGTLCGLIALIGAVGNGTVIWICLYYRRMRTITNYFIANLAG
ADFLLCLLNVPLTSYFILTQNWPFGHELCTFINFIASTTVVASVFTMMCISIDR
YIAIVHPMRPRMSSTASYIIIFLIWSLAGLISLPPLLYSTTHHDELTGCTICSLSW
GGEGEDTAAAAAAATANSNYQIFYMVITYFVPLFALTFAYGRVANELWGSKS
IGESVSEAQLESVRSKKKLVKVLIWVMVTFMVCWAPYHCWFIITGIDPCVQQ
FDNISYVFTSAYVLAMSNSIYNPIIYIILNNKFRQGFRQVFRWLPCIHWKSSEAI
DHPHTAAGIRDRSSCTEFSTVKNGSIGALNSSLTLQHTRNNGRTVAAYKNN
VEKVHLVSRKGGVTSATAATAATALGGDREDTSFASGSAALADSLHSF
>Ixosc_RYaR
MDSTNGPSAPPTATSNWTSQPASTESAACDLPPPVPEGMQALMYIMYIAVS
VAAIGGNDIVCYIVLAYQRMRTVTNMFIMNLAIGDILMASLCIPFTFVSNLLLG

YWPFGGVMCVVVTYAQCVTVFISACTLIAISVDRYTAIVYPLRPRMTKLRSKII
IGVVWLVALVTPLPTALVTQLVPHPCANQTYYCLEQWGTPEQATYYSMALMI
LQYFFPLLALIFTYTRIAVVVWGKETPGEAQDERDQRMAASKRKMIKMMIAC
VAAFLLCWLPLNLFIVVSEQYPDVYDLNGIGYVWFVCHWLAMSHTCYNPLIY
FWMNAKFRTGLQAVFRCWHVPKKKSSCFVSTVKKVSSASGNTT
>Lymst_LuqR
MSMANSENSTSLFGIKRHADVTGPHSASHDVIDPSNTSVYYDHASNYESVL
STTSTLMLKLTDLVTPFNASEPDPESNGSDTDGGHAAISEQPMYAKVIIVLMY
VLIILVAVGGNLLFSYVIVMYPKMRSVTNLFLLNLAISDIVKAVICNPFAFIANLIL
LYWPYGEFMCQVVTYIQVVAVFLSAFTLVAMSVDRYVAILKPMRPRLSKRAF
AITMATIWILSLSAPLPTAITSRVTKQSNSTGLCLEHFENDHNRYIYSIVIMMLQ
YFVPLAVITVTNTHIGYIVWIKKTPGEAEEDRDRRMAASKRRLVKMIIIVVVIYA
VCWLPVHVITLVGDHNPDIYNQPHMNVVWLCAQWLAMSHSCYNPFVYFSL
SATFRRNLRRMTHACRLKQKRLRQHLSMRSSRADAWDRDTEVYGSAESIP
SKVSAGSLHSSNRGAKHVNTSSGEWQCLKEKKLKGVSNDMYL
>Musmu_NK1R
MDNVLPVDSDLFPNTSTNTSESNQFVQPTWQIVLWAAAYTVIVVTSVVGNV
VVIWIILAHKRMRTVTNYFLVNLAFAEACMAAFNTVVNFTYAVHNVWYYGLF
YCKFHNFFPIAALFASIYSMTAVAFDRYMAIIHPLQPRLSATATKVVIFVIWVLA
LLLAFPQGYYSTTETMPSRVVCMIEWPEHPNRTYEKAYHICVTVLIYFLPLLVI
GYAYTVVGITLWASEIPGDSSDRYHEQVSAKRKVVKMMIVVVCTFAICWLPF
HIFFLLPYINPDLYLKKFIQQVYLASMWLAMSSTMYNPIIYCCLNDRFRLGFKH
AFRCCPFISAGDYEGLEMKSTRYLQTQSSVYKVSRLETTISTVVGAHEDEPE
EGPKATPSSLDLTSNGSSRSNSKTMTESSSFYSNMLA
>Musmu_NK2R
MGAHASVTDTNILSGLESNATGVTAFSMPGWQLALWATAYLALVLVAVTGN
ATVIWIILAHERMRTVTNYFIINLALADLCMAAFNATFNFIYASHNIWYFGSTFC
YFQNLFPVTAMFVSIYSMTAIAADRYMAIVHPFQPRLSAPSTKAVIAVIWLVAL
ALASPQCFYSTITVDQGATKCVVAWPNDNGGKMLLLYHLVVFVLIYFLPLVV
MFAAYSVIGLTLWKRAVPRHQAHGANLRHLQAKKKFVKAMVLVVVTFAICW
LPYHLYFILGTFQEDIYYRKFIQQVYLALFWLAMSSTMYNPIIYCCLNHRFRS
GFRLAFRCCPWGTPTEEDRLELTHTPSISRRVNRCHTKETLFMTGDMTHSE
ATNGQVGGPQDGEPAGP
>Musmu_NK3R
MASVPTGENWTDGTAGVGSHTGNLSAALGITEWLALQAGNFSSALGLPVTS
QAPSQVRANLTNQFVQPSWRIALWSLAYGLVVAVAVFGNLIVIWIILAHKRM
RTVTNYFLVNLAFSDASVAAFNTLANFIYGVHSEWYFGANYCRFQNFFPITA
VFASIYSMTAIAVDRYMAIIDPLKPRLSATATKIVIGSIWILAFLLAFPQCLYSKI
KVMPGRTLCYVVQWPEGPKQHFTYHIIVIILVYCFPLLIMGVTYTIVGITLWGGE
IPGDTCDKYHEQLKAKRKVVKMMIIVVVTFAICWLPYHVYFILTAIYQQLNRW
KYIQQVYLASFWLAMSSTMYNPIIYCCLNKRFRAGFKRAFRWCPFIQVSSYD
ELELKTTRFHPTRQSSLYTVSRMESVTVLYDPSEGDPAKSSRKKRAVPRDP
SANGCSHREFKSASTTSSFISSPYTSVDEYS
>Octvu_TKR
MNASQKISAFSLARTTISSLLESQAVQDKSSSYDVTIDWADNTTTEELSTLAP
TNPFILPWWQQVFFIIIFLAMIIASIGGNLIVMWIVLWHKRMRTVTNYFLFNLAL
ADALISVWNTLFNTAYLLYSNWWFGEDFCKFSMFVAPCTTSASVFTLMAIAI
DRYLAIMRWVRMSAKVVIGLIVVIWLASCLISLPLAIYSKTETFSYADGSTRTL
CLQEWPGNQRSSSVELGYNIFLIIVNYFLPMFILIVTYTFLGKELWGSKAIGEN
TSIQQQRVKAKQKVVKMMIVVVIIFAVCWLPMHLYFLLVSSFPSINSYQYIQQI

FLIIYWMAMSNSMYNPIIYCWMNARFRQGFKLVFCIFPCVHVQKKRRPDRN
MTLSMSMSDTKGVNRNGSLMHTTMENMEESYNSPETIQETDWKQNSKDD
PAADDEYL
>Pladu_LuqR
TMLNSSAYGNGTELSAEDFSMPTYAQVVMILMYTTITVLAVGGNSIVVYIVLA
YQRMRTVTNYFIVNLSLSDIIMSCICIPFTFIANMLVHYWPFGAIMCPLVTYAQ
AVAVFLSAFTLVAISLDRYVAIIYPLRPRMTTNQAALSIFLIWFLSLAVPLPIAIV
SKVQPALQPTGVVRDECRENWPKAEQRYVFSVVIMVLQYFLPLFVLLFTYT
RIAIVIWVKRTPGEAENNRDQRMAASKRKMVKMMITVVIIYALCWLPLHTVTI
AGDLHPAVYEFRYIQVVWIACHWLAMSNSCYNPMVYCWMNSKYRNGFRY
VLRCCPFISYKEDDHGPYQMKRINTYISTMRSSTHDRKVMTTFSASPSPKRS
KDKFETTICKEEIALNHMSNGTNSTQWDGNIDGSERVPLAEHNDA
>Ramva_TKR
MELFDPNDNHTLVPNPVPLPLCEGYGSEDGSPQEYHLPLGWQYGYGLLCAI
ISIMGAVGNGIVIWICLYYRRMRTITNFFIANLAAADLLLSICNVPPTSYFILTQN
WPFGQAACAFVNFIATTPIVASVLTMMCISIDRYIAIVHPMRPRMSKYTAYVII
VAIWLTGGSVSFPALVYSATFANGNTTICSLQWDAPQSEKKVNSEFIYQFAY
MFITYFVPVVALTFAYGRVAKELWGTKSIGEISVAQIESIKSKKKLVKVLIWVM
TTCAICWAPFHIWFIVTSINRCVQLLKHASHIFTSSYVLAMSNSVYNPIIYVILN
QRFRQGFKQVFQWLPWVHFDPSEALDRPQRGTGQHHNITRDAFSSCHDM
NTMRNGGSSHALANSHSTLNNHNGTHRTSGASTFQSPTSRNTLTVDESTR
NRLLPSFALQDADGCRLGKKGMAKCDSIYSF
>Sacko_LuqR1
METADLTVVQHNITDYLWFGNITQNNPGLRHQVLPMMGYYQPLWLQIILAIL
YACTSILSVVGNLVVCYIVLGNRRMRTVTNYFIVNLAISDILMAVLCVNLTFYY
SINFHWPFGFVMCALVQFVQMVSVSISIFTLVAISLDRYVAIIHPLRPRMTKKQ
ACVVIITIWVLAASISLPAGINSEIDIPANSSLIGCTEKWQTDKQRSVYTITLMIL
QYFLPLVILSITYSIIGYAIWGRKPPGERERHRDARQAESKKKLVKMFALIVLIF
ALCYLPIHTFNLLLDYEPDVGFFRYIKLVYFAVHWTAMSNCIYNPFVYCWMN
AKFRDGFKYVFRFLPCVHYIPKDKFPVKRVNTCTTHVGSFKMNLLRENALSP
YVNGRARGMR
>Sacko_TKR1
MALSSSYVASLNFTLYSTLAPSIEYDYEYNWNETCQDFQQPIIETFIWTLLFG
AMIVTAVVGNCIVMWIVLAHRRMRTVTNYFIVNLALADALNAIFNISFTFTYVL
RNDWYFGNAYCKIVRFISPLTVASSIFTLMAISIDRYIAIVHPMRPRMSKVLAK
TIIAVVWIVSAVIALPWLIFTNIDFGACPPPAITDTPIYVRRVCATIWPDQENYG
DWYFWYSFSFMVVTYILPLIAQGVSYSIVGIKLWGSHAPGEISNRHREQLKA
KRKVVKMMILVVVLFAICWLPVHIYFLLGRSYSDVLYSHPNAREIYMAVFWLG
MSNSMYNPFIYCWLNDRFRRGFRKALRWLPCFKWAPGERVDSQRPATLS
GMYSTTQGMKLNEERIRHDNN
>Sacko_TKR2
MALSSSYIASLKFTINSTRAPSVEYDYEYNWNETCQDFQQPIIETFIWTLLFG
AMIVTAAVGNCIVMWIVLAHRRMRTVTNYFIVNLALADALNAIFNISFTFTYVL
RNDWYFGNAYCKIVRFISPMTVASSIFTLMAISIDRYIAIVHPMRPRMSKVLAK
TIIAVVWIASAAIALPWLIFTNIDFGACPPPAITDTPIYVRRVCATIWPDQENYG
DWYFWYSFSFMVATYFLPLIAQGVSYSIVGIKLWGSQAPGEISNRHREQLKA
KRKVVKMMILVVVIFAICWLPVHIYFLLGRSYSDVLYSHPNAREIYMAVFWLG
MSNSMYNPFIYCWLNDRFWRGFRKALRWLPCFKWAHDERVDSQRPTNLS
GTYSTSQGMKLMRNGSHMTTAVDESM
>Sacko_TKR3

MSLSSAYIASSNFTLNSTLVPSVEYDYEYNWNETCQDFKQPIIETFIWTLLFG
AMIITAAVGNCIVMWIVLAHRRMRTVTNYFIINLALADALNAIFNISFTFTYVLR
NDWYFGNAYCKIVRFISPLTVASSIFTLMAISIDRYIAIVHPMRPRMSKILAKTII
AVVWIASAAIALPWLIFTNIDFGACPPPAITDTPIYVRRVCATIWPDQENYGD
WYFWYSFSFMVATYFLPLIAQGVSYSFVGIKLWGSQAPGEISNRHREQLKA
KRKVVKMMIIVVVIFAICWLPVHIYFLLGRSYSDVLYSHPNAREIYMAVFWLG
MSNSMYNPFIYCWLNDRFRRGFRKALRWLPCFKWTQGERVDSQRPTTLS
GTYSTSQGMKLMRNGSDMTTTVDESMLENT
>Sacko_TKR4
MASNSSYIASLNFTLHSTLAPSVESDNEYNWNETCQDFQQPIIETFIWTLLFC
AMIVTAAVGNCIVMWIVLAHRRMRTVTNYFIVNLALADALNAIFNISFTFTYVL
RNDWYFGNAYCKIVRFISPLTVASSIFTLMAISIDRYIAIVHPMRPRMSKILAKT
IIAVVWIASAVIALPWLIFTNIDFSACPPPKITDTPIYVRRVCATIWPDQENYGD
WYFWYSFSFMVATYFLPLIAQGVSYSIVGIKLWGSQAPGEISNRHREQLKAK
RKVVKMMILVVVIFAICWLPVHIYFLLGRSYSDVLYSHPNAREIYMAVFWLGM
SNSMYNPFIYCWLNDSSYILCVFCG
>Sacko_TKR5
MALSFSYIASLNFTINSTIAPSVEYDYEYNWNETCRDFQQPIIETFIWTLLFGA
MIVTAAVGNCIVMWIVLAHRRMRTVTNYFIVNLALADALNAIFNISFTFTYVLR
NDWYFGNAYCKIVRFISPLTVASSIFTLMAISIDRYIAIVHPMRPRMSKVLAKTI
VAVVWIASAAIALPWFIFTNIDFGACPPPAITDTPIYVRRVCATIWPDQENYGD
WYFWYNFSFMVATYFLPLIAQGVSYSIIGIKLWGSHAPGEISNRHREQLKAK
RKVVKMMILVVIIFAICWLPVHIYFLLGRSYSDVLYSHPNAREIYMAVFWLGM
SNSMYNPFIYCWLNDRFRRGFRKALRWLPCFKWAPGERVDYQRPTNLSGT
YSTTQGMKLMKNESDMITTVDESMLENT
>Sacko_TKR6
MAVTVLPVVAYNFTSFYDDYEIHNISNTTVDVAQCSVSAFVQPVLTQFLWSLI
FGGLIIIAAGGNIIVIWIVLAHRRMRTVTNYFLLNLAFADALIAILNIPFNFSFLLN
NIWYFGFVYCKIAKFTGTLMVTASIFTLSAIAVDRYIAIVHPMKPRMSKTKAKSI
IFLVWVLSGVICFPNLVYAQLIYIPCFDGVRIVCILDFPDGIYNEYDFWYNISCM
VITYFLPLAAMGVSYGIVGVKLWGSHAPGESSSRHKEQLKAKRKVVKMMVI
VVLLFGICWIPVHIYFLLGRHHSYLYKYEHIQEIFLFLYWVAMSNSMYNPLIYC
WMNDKFRKGFKKALRFLPGVHWKPEDSIDAPRPRGKLVSSLYLVPLRYGIG
KVTYTLQSNNTHTTKYVSAFVLVGQSNTLSDTAPWGKDPQDSRKAEPRLAQ
DWTASRLAPGLSRTQTASVVEYSPRPTQTLFIHFQNSNQLQNSKKTLRRKL
Q
>Tetur_NTLR
MELLINSSMIPIVKDWKSLCAFENTQCIDLITFLNKSDLESLLTLYNDSLVTDP
SFAFASSVSSSISGSSFTSSSASSASAGSSKFVPELVVQVIWSIVFGLMILVA
MVGNTTVIWIVTGHRKMRTVTNVFLLNLTIADLIMATFNGVFNFVYMLHSHW
PFGQTYCKINNFIANVTVASSVFTITATSIDRYIAVVHPLKPRMTKHQVMLIIIM
VWSLAALLSLPNLLYSELFTIPFQDGGYRVVCILVWPDGYAGQSTLDYLYNL
TFLAITYVIPMISLIITYSLMSRVLWGSKGIGEETDIQRESIRSKQKVARMLIALV
VLFAICWLPYHAYFLYSYHYPHINQSVIIQHIYLFSYWLAMSNSMYNPLVYYW
MNKRFRSYFASIFCFFSRNRLKKQLSYNVKTDLSSNGFQHGTTHYRRTPVQ
SSNQIELHQHKVKP
>Tetur_RYaR
MKFPMLAEVLMYFMYSSICVIAITGNAIVCFIVIAYRRMQSVTNYFIVNLALSDI
LMASLCIPFTFVSNLILQHWPFGAFMCVLVSYSQAVSVFISAYTLIALSLDRYI
AILYPLRPKMTRFQAKLIIACVWIVALITPLPTAIVSKLVQPAVWVESGITDRYT

CTEDFANQTYRQVYSLALMILQYFFPFVVLLYTYTRIAIAVWGKTTPGEAEDA
RDKRLAASKRKMIKMMITCVTAFTLCWLPFNTFIVVGDNHPEIFSYSYIIYIWF
ACHWLAMSHACCNPIIYCWMNTRFREGFKYIFRFLPFIKKPCKPDFMISSSA
QSSQGATLRCYIGSRIKNGNLGKAQAHLQEVTSVEEIPLETK
>Tetur_TKLR1
MALQQPEVNLTYQRCSPSLLNSSSPCTDCASPLCLNMSSVFNLSLAALIHQE
AYSSIPSSLSSFSSSLPSSPSSVVSAAVATAAQPATTSTSPDDNSKAYFISFN
VKLLWIVVYVIMIMMALLGNLGIIWIITGHRKMRTVTNIFILNLSIADLITSMLNIT
FNFIFMLSGQWYFGYFFCKINTFIAYVTLSASVFTIMALSLERYRIVINPMKPRI
TKTTCVACLILIWLFSFLLSLPTIVYATYITYDMNGNYTRKICLLQWPDGLPGR
SEADFYFNLVIFAVDYAIPMVLMSVTYYRIGRVFWGSQAIGELTEGQREAIRG
RQRVVTMLITVTVLFAICWLPYHIYFIYIKYDPNIITHKYISNIYLTIYWLAMSNS
AINPVIYAILSKRYRTYFLKMIPCKLGKRAFDKDKSMKTSQKTVEIEML
>Tetur_TKLR2
MDYAQYKSLVNTSCDQSIVTDYLDQNVIEKCFSNNLYLSTTQSLVLVSTQST
LSSTESSTTVDGYRPFVPSLGLQFFWTVIFSLNVISSIIGNLIVILIICGYKQMRT
KTNLFLLNLSVADLMMATFNTMFNFIFMLKSHWPFGTTYCIINNFLSSVATSS
SVFTITVTCFDRYMAVVHPFKPRMNRTTSLIVVLLIWLLAGVLAIPTVLYSTTY
ELSYQDGSRVLCWIKTILSCLRRLHSNAENIYNVVFFVVTYAIPMFTMGLTYT
RMSFVLWGSKQIGEMTSHQLEAIRSKQKVVPMLIIVTIIFGLCWLPYQIYFLVV
FYIIELGQKEYVQHIYLAFYWLAMFNSSLNPIIYCLLNKRFRKYSKKIIITITHCG
KNHVINHRGVSIRSEDCSNMDHQTVNLSSHATRLDYKSDNL
>Tetur_TKLR3
MMKESSSGSRIYNLVDFYNQSDPLFWSNVTECVTSASDLTNGLIDSQSYSD
TSTRIPVVPSLGPGWTNSDSYLYPNQSQPGATNSSINLSTSRLLVECLKLRIG
SAPYILVPWARVIWILLFTGIITVTVIGNALVIWIILAHIRMRTITNLFLLNLALADL
LMALFNTAFNFTWMLNSHWPFGSVYCSINNFIAHLTVFSSVFILTVMSIDRYI
AIVRPLSSRMSRRTTLITLNLIWIIGAIISLPNLLFSMTVTYIYPDGSLRTVCLLK
WPDGFAGYSSFDYIYNILFLVITYFVPVITMAATYPQMISVLWGSQNIGEITER
LKSAIKAKRKIVRMLISVVFIFVICWLPYHIYFLIVFHFPKISHYKNIQNIYLAIYW
LAMSNSIYNPVIYYKMNGRFRRYFQSILCFCRSTQKDPGDSKWNNLSMHRR
VAGKRFYPNNMNNTGANVNHSTRSTRSNTCRSLTNGSVDVARMTEINSNC
NP
>Tetur_TKR
MYDNSMNISLFSNGVNNESYPGLTFSSTNSSSQFNLTNSDVYFINNSNYPQ
NNWSVSSSSPSPSDVNPLERNKFILPWWQQVIWTLVFGGMILVATGGNTIVI
WLVLAHKRMRTVTNYFIVNLSIADIMVSTLNVIFNFTCMLNGDWPFGTLYCKI
SNYIAIVSVSASVLTLMAISIDRYVAIIYPLKPRMSKKTTVFITLAIWIIGSILSLPN
ILYSTTKREYFLNGDYRDICFLDWPDGPATSSKFDYIYNVIILLVTYMVPICSM
CFTYSRVGLELWGSKGIGEYTEKQVESMKSKRKIVKMMIVVFVIFAVCWLPY
HGYFLLTHHFPEMMEADYVQQIYLIIYWLAMSNSMYNPIIYCWMNSRFREGF
KKIFCCLCYDNSDSDNFLESKLTRRGKSSFSEACITETKIRLNGNGHGTALM
MDETIKQAEYRL
>Trica_NTLR
MSTTESWKEEEGNGSFQGFDYGLLVPISSQTPVVVDSGENFMPIWVKVLW
TVIFMVMILIATGGNCIVIWIVTAHRRMRTVTNYFLVNLSLADLLLTTFNCIFNF
SYMIQRDWPFGSLYCIISNFIANATVAASVFTLTGISCDRYLAIVHPLQPRMSK
RASLITITFIWLASMTVAFPCLLYSTTITNKYKGVERTGCILIWPDGKVVGSHF
DFAYQMFFLIITYVIPVTLMSFSYTIMGKELWGSRSIGEMTQRQIDSIRSKRKV
VKMFIFVVFIFAICWLPYHGYFLYVYYDTDIIFSKYTQHVYLAFYWFAMSNAM

VNPLIYYWMNARFRQYFKTAICGWKECILHKKCQGDDSSLHTECRQSHSGF
YRSRKSKREDKLGADSPNNVASPTTKTHEFNETFKAKNNQRWVRSSFRND
SKRTTVNL
>Trica_RYaR
MDANTTRNESFSLDCELVNPNSTLANVYFLSAVYSMYAIIFVVALIGNSFVCYI
VLSSPPMRTVTNFFILNLAIGDVLITLLCVPFTSVSLLMQYWPFGGILCPVVNY
SQALSVFVSAYTLVAISIDKYMIIMWPLKPRISKRFATYIIALVWLIAGITVLPSA
TFTTLINDENILGTSAYEQCDKYICAEEYSKVGQEYGDLYTKVLMFLQYVIPS
LVLLFTYTSIGVVIWCHRIPGEAENSRDQRIAKNKTKMIKMMVTVVCVYTICW
LPYNVLMIFKEHISGSVMVYLYFPLHGLAMSHACYNPIIYCYMNARFRNGFL
QVMMSIPCLRRCNSINDISKILTCRWKVRRVHLYREITRAQPTSA
>Trica_TKR
MNFTQEFLYTTGYSIMDNSSEYDYTTNISYNDTEEGGNQFILPVWRQVLWSI
LYAGMVIVATGGNLIVIWIVFSHKRMRTVTNYFLLNLSVADTMVSTLNVTFNF
VYMLNSHWPFGELYCKISQFIAVLSVCASVFSLMSISIDRYMAIMTPLRPRMG
RTVTVLLAVTTWLLGVIIGSPSLMFFRTYTMPYKDGEERVICYPEWPDGTTN
ESMMEYAYNVGFLFVTYVVPIGSMTYTYARIGIELWGSQSIGECTQRQMENI
RSKRRVVKMMMVVVIIFAVCWLPYHLYFIVISYFPEITNSTYIQETYLAIYWLA
MSNSMYNPIIYCWMNARFRRGFKQFFSCLPFIHVSPGALTRREVLTSRRRS
YSGSPDHNRIIRNGTIRMNYMTRPSPTSSTNTCYSNLPDELGYHRNLEARR
WKGEELRSVS
>Varde_NTLR
MTFTMNQLAAVALNTTVSRCLKALNDSLSTTSPAAEVPADCVSLFDNVTSAD
GNITDLTSLGNSSFPGYVEGGSSLTFLLPMWQQVTFIMCFSLIVFAGILGNAI
VIWIVLAHQRMRTVTNYFLVNLSVADLTTAMFNVIFNAVFMMHSHWPFGALY
CRITNFISLLTVTSSVFTIKAMSIDRCIAIYNPLSHRLSRRCALIIVALIWLVSCVI
ALPGYAFAQTRSYEDRIVCSLSWPELGGVNPSQVDFIYNVVFMIATYFVPMV
TIAIAYSIMGHVLWRSKGIGEQTERQKEAIRSKQRVVRMLVVVVMIFGVCWL
PYHLYFIYTYLDPDVTYTTWAQPLYLVIYWLAMSNCMYNPFIYYWMNSRFR
GYFRYVLCYCCNLGSGLGHKCSEVDRLQSDWTFNNNSPHRRTLKSTAGTE
CLVLTTPPNNRNATGSQQKHPHSHKNGSWQVHSDSSTVTYVL
>Varde_TKR
MDVLEEMNLTFFNISEVFKIYRSNFSFEDTDYALFMPIYMEVIWCILFSVMIVV
AACGNLIVIWIVLAHKRMRTVTNYFIVNLSIADTMVSTLNVIFNFTFMLRSEW
WFGEWYCKFSNFVALVSVSASVFTLMAISIDRYMAIMHPLHPRMSRMMTLNI
ALCIWLLAGLLACPQYVYSRVKEQDNHTVCYMFLDEDGEITESRADYLYNLV
VLIVTYIIPMQAMAFTYFRVGRELWGQQSIGEVTRKQTEAINSKRKIVKMMIVI
VAIFGVCWLPYHLYFLLVHHYPDMRNSVYIQNIYLTIYFLAMSNSMYNPVIYC
WMNSRFREGFKAVFCCYALTGKQLSAFAKNNRKFARYSCASEPYSTTRVTL
NHLHTTQNINNHQLISNQADIDLQHQPDGSASTEPGTGENGIHRLLRSQQIRI
QRNSQLPFANGHTEV
