# Supplementary material for: Tachykinins: Neuropeptides That Are Ancient, Diverse, Widespread and Functionally Pleiotropic
Source: Front Neurosci. 2019 Nov 20;13:1262. doi: 10.3389/fnins.2019.01262 (PMC6880623; doi:10.3389/fnins.2019.01262)
Supplement: Supplementary file 2 [file Data_Sheet_2.PDF]

Supplementary Material

Tachykinins: neuropeptides that are ancient, diverse, widespread and functionally pleiotropic

Dick R. Nässel*, Meet Zandawala, Tsuyoshi Kawada and Honoo Satake

*** Correspondence:** Dick R Nässel, Department of Zoology, Stockholm University, SE-10691 Stockholm, Sweden, e-mail: dnassel@zoologi.su.se

1 Supplementary Figures and Tables

1.1 Supplementary Figures

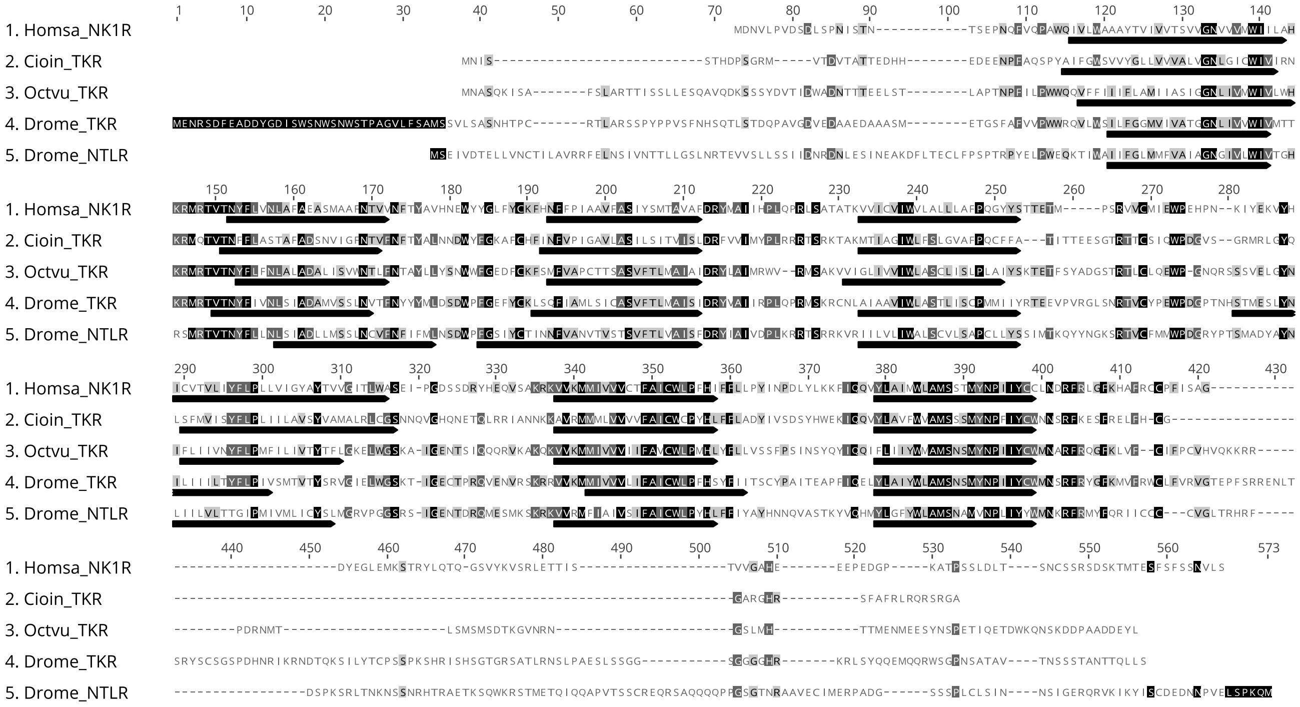

Supplementary Figure 1. Multiple sequence alignment of select TK and Natalisin receptor amino acid sequences. Transmembrane domains I-VII are indicated by black arrows. Conserved residues have been shaded according to the following scheme: 100% identical, black; 80% to 100%, dark grey; 60% to 80%, light grey; less than 60%, white. Abbreviations: Homsa, *Homo sapiens*; Cioin, *Ciona intestinalis*; Octvu, *Octopus vulgaris*; Drome, *Drosophila melanogaster*; TKR, Tachykinin receptor; NTLR, Natalisin receptor.

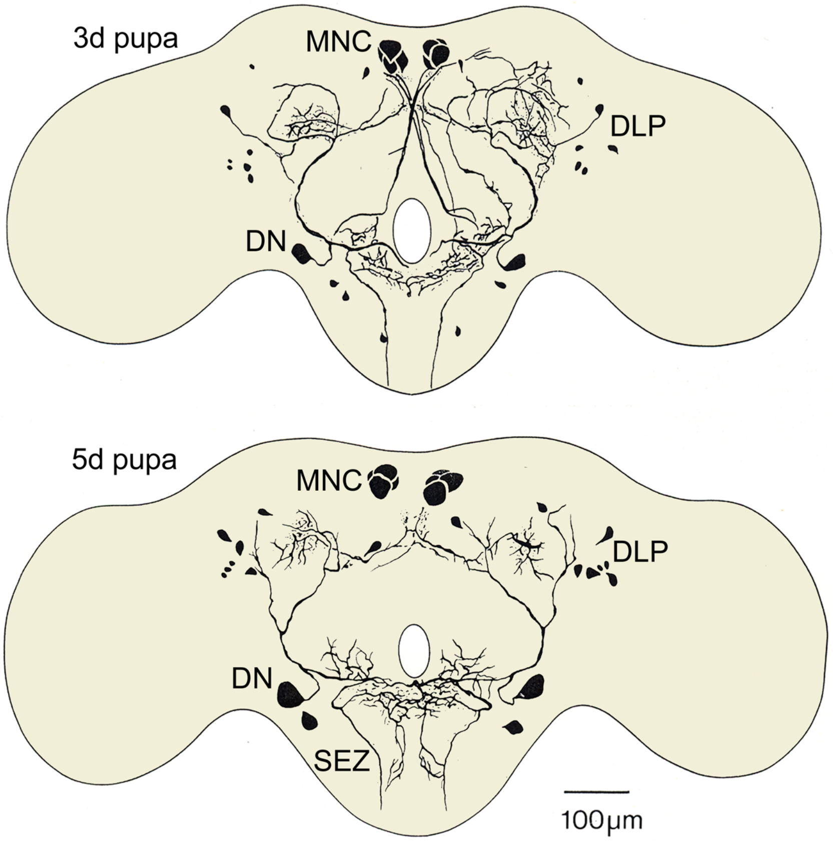

Supplementary Figure 2. TK immunoreactive neurons in the brain of the moth *Spodoptera litura*. The brains of 3 day and 5-day-old pupae are shown. The major TK neurons can be distinguished at this stage; only a smaller number of additional small neurons can be seen in the adult brain, especially in the subesophageal zone (SEG). A set of 8 median neurosecretory cells (MNC) expresses TK, and also a pair of descending neurons (DN). A bilateral cluster of TK neurons is also found in the dorsolateral protocerebrum (DLP). Image slightly altered from (Kim et al., 1998), with permission.

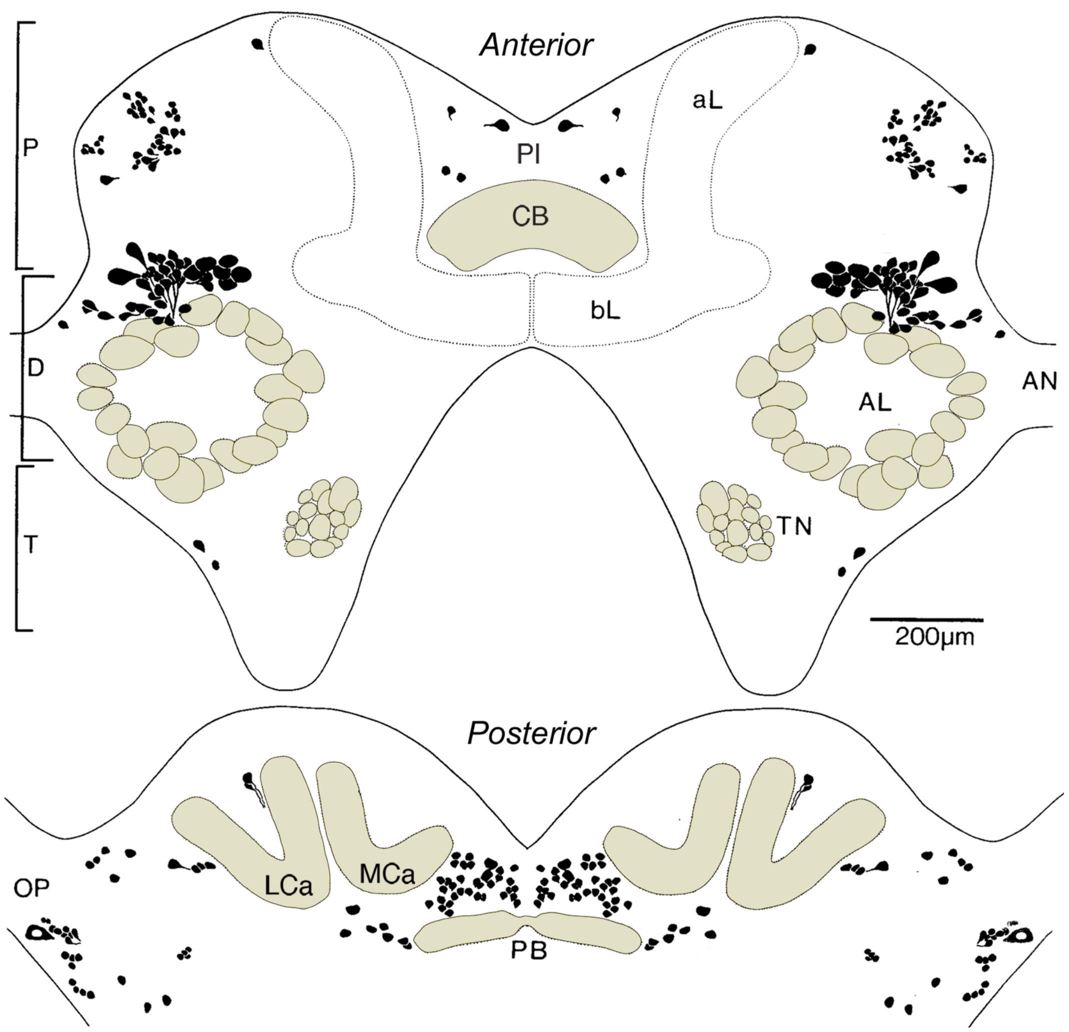

Supplementary Figure 3. TK immunoreactive neurons in the brain of the cockroach *Leucophaea maderae* (new name *Rhyparobia maderae*). Neuronal cell bodies are shown in black and neuropil regions innervated by TK processes are colored: central body (CB), antennal lobe (AL), tritocerebral neuropil (TN), calyces of the mushroom bodies (LCa and MCa) and the protocerebral bridge (PB). No labeling was detected in the alpha and beta lobes (aL and bL) of the mushroom bodies. However TK fibers are abundant in the pars intercerebralis (PI; not shown here). P, protocerebrum; D, deutocerebrum; T, tritocerebrum; AN, antennal nerve; OP, optic peduncle. Slightly modified from (Muren et al., 1995), with permission.

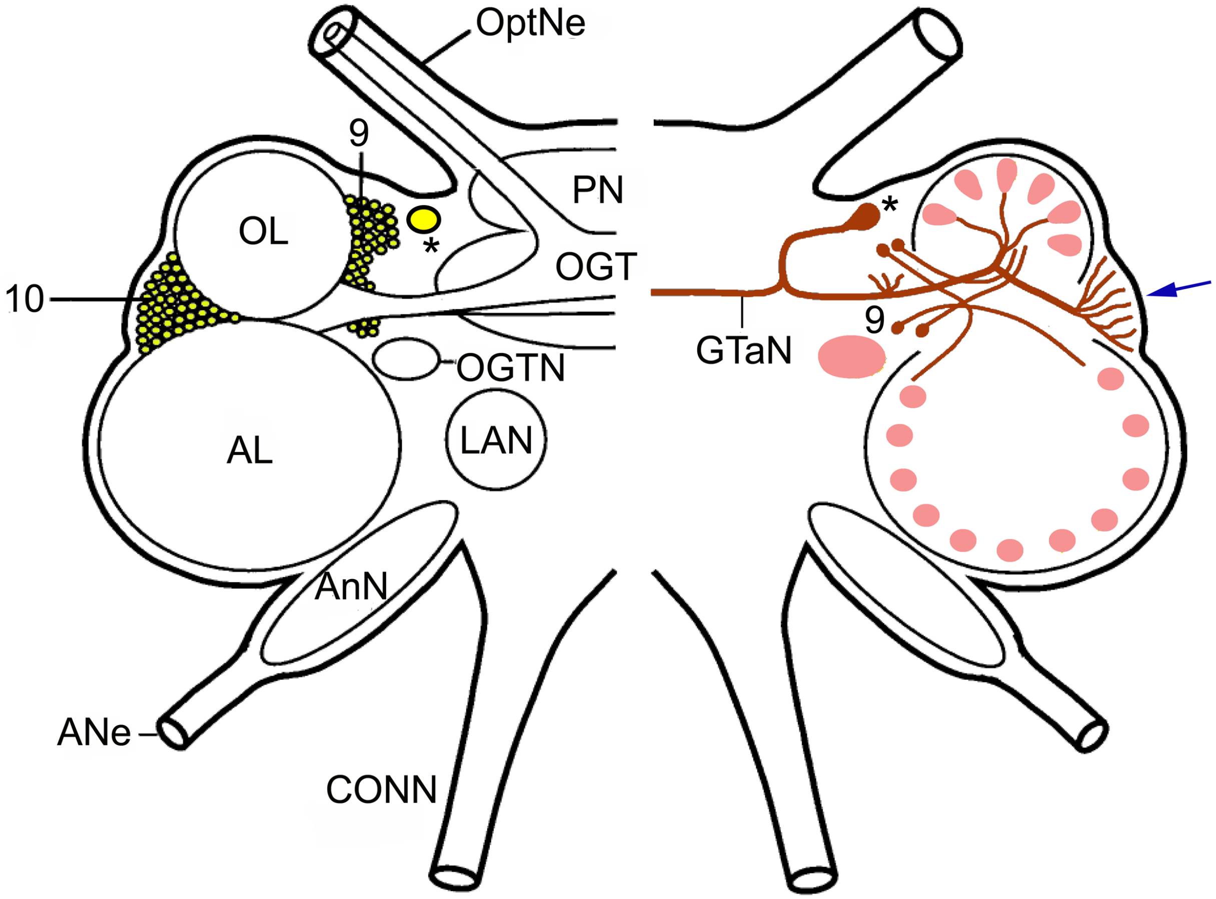

Supplementary Figure 4. Schematic depiction of TK immunoreactive neurons in the brain of the crayfish *Pacifastacus leniusculus* in horizontal view. In this figure only neurons associated with the olfactory midbrain are shown. This consists of the olfactory lobe (OL), accessory lobe (AL), lateral antennular neuropil (LAN), antennal neuropil (AnN) and the olfactory-globular tract (OGT). Cell clusters 9 and 10 are indicated (yellow). The TK expressing neuronal structures (red) and their arborizations in the OL and AL glomeruli (pink) and in cell cluster 10 are shown on the right side. The small olfactory-globular tract neuropil (OGTN) also contains immunoreactive fiber arborizations (pink to the right). One of the "giant TK-immunoreactive neurons" (GTaN) is shown schematically (cell body indicated by asterisk) and so are a few of the immunoreactive neurons of cluster 9. ANe, antennal nerve; CONN, connective; PN, protocerebral neuropils; OptNe, optic nerve connecting the brain with the eyestalk neuropils. Slightly altered from (Johansson et al., 1999), with permission.

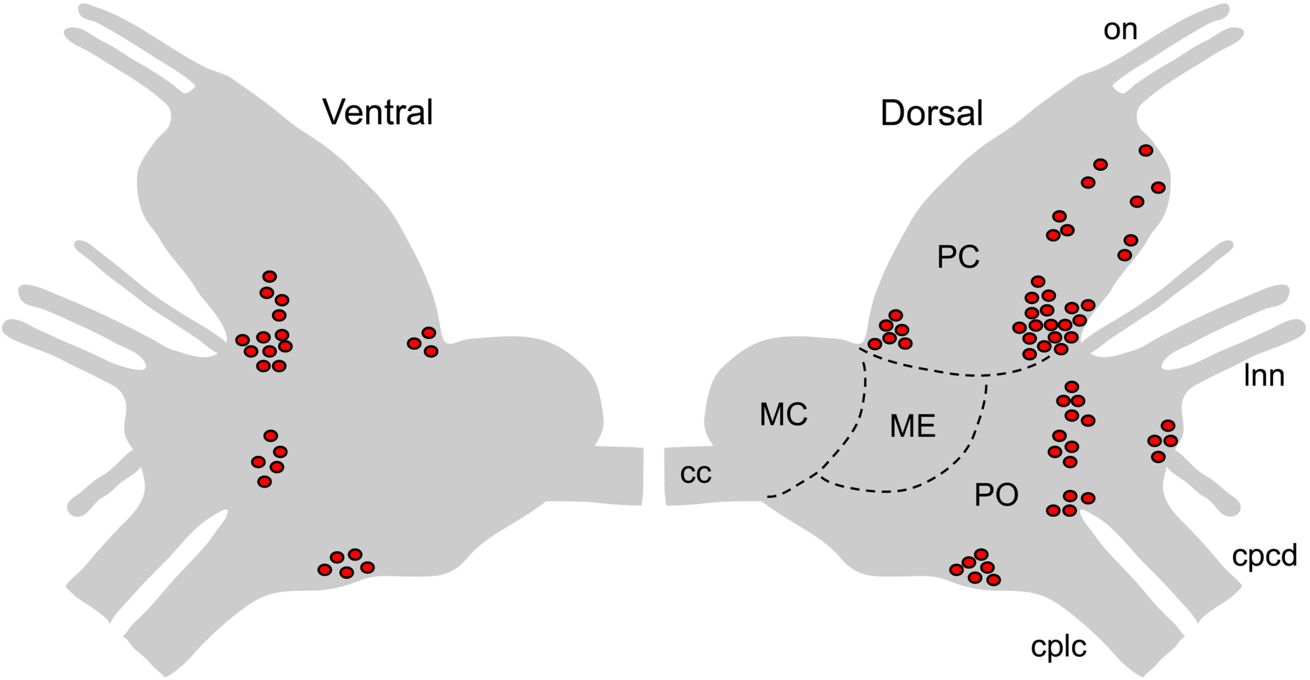

Supplementary Figure 5. Schematic depiction of TK immunoreactive neurons in the cerebral ganglia of the snail *Helix pomatia* in horizontal view. Right side shows dorsal surface; left side ventral surface. Neuronal cell bodies are shown in red (note that accurate numbers are not shown). PC, procerebrum; MC, mesocerebrum; ME, metacerebrum; PO, postcerebrum; cc, cerebral commissure; on, olfactory nerve; lnn, lip nerves; cplc, cerebro-pleural connective; cpdc, cerebro-pedal connective. Slightly altered from (Elekes and Nässel, 1994), with permission.

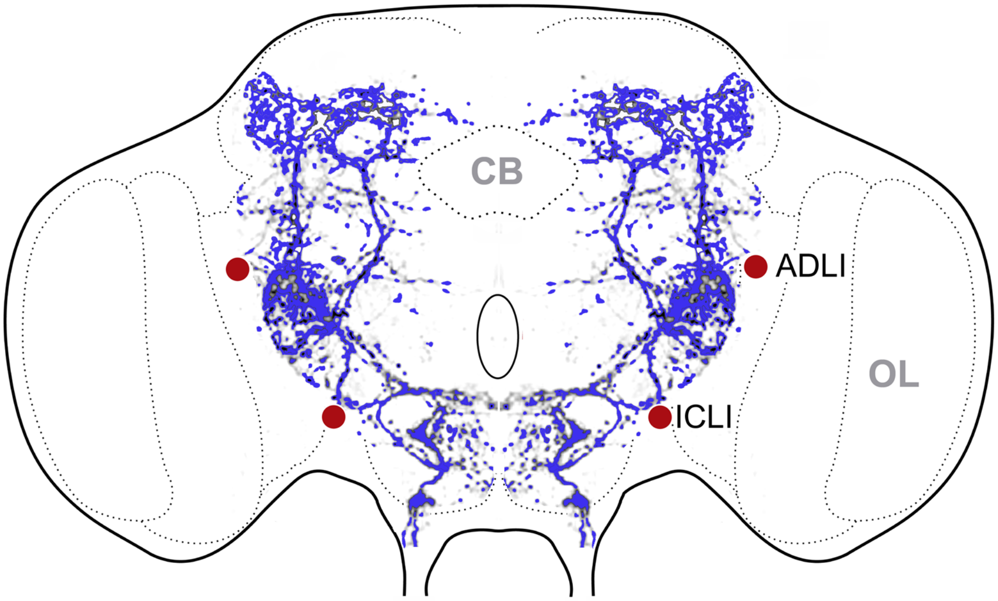

Supplementary Figure 6. Semi-schematic depiction of natalisin (NTL) expressing neurons in the brain of *Drosophila*. Two pairs of NTL neurons were shown with a NTL-Gal4 driver and antiserum (red cell bodies and blue processes). These neurons were designated ADLI and ICLI. Note that the NTL process do not innervate any of the major structured neuropils, like the central body (CB), optic lobes (OL) or others not shown (mushroom bodies, antennal lobes and so on). The NTL processes were traced from an image in (Jiang et al., 2013) and superimposed on a brain outline.

References to Figures

Elekes, K., and Nässel, D.R. (1994). Tachykinin-related neuropeptides in the central nervous system of the snail *Helix pomatia*: an immunocytochemical study. *Brain Res* 661**,** 223-236.

Jiang, H., Lkhagva, A., Daubnerova, I., Chae, H.S., Simo, L., Jung, S.H., Yoon, Y.K., Lee, N.R., Seong, J.Y., Zitnan, D., Park, Y., and Kim, Y.J. (2013). Natalisin, a tachykinin-like signaling system, regulates sexual activity and fecundity in insects. *Proceedings of the National Academy of Sciences of the United States of America* 110**,** E3526-3534.

Johansson, K.U.I., Lundquist, C.T., Hallberg, E., and Nässel, D.R. (1999). Tachykinin-related neuropeptide in the crayfish olfactory midbrain. *Cell and Tissue Research* 296**,** 405-415.

Kim, M.Y., Lee, B.H., Kwon, D., Kang, H., and Nässel, D.R. (1998). Distribution of tachykinin-related neuropeptide in the developing central nervous system of the moth *Spodoptera litura*. *Cell Tissue Res* 294**,** 351-365.

Muren, J.E., Lundquist, C.T., and Nässel, D.R. (1995). Abundant distribution of locustatachykinin-like peptide in the nervous system and intestine of the cockroach *Leucophaea maderae*. *Philos Trans R Soc Lond B Biol Sci* 348**,** 423-444.

2 Supplementary Tables

1

Supplementry Material Table 1. Tachykinin sequences from select invertebrates

Order	Species	Copies	Sequence / method	References
**Insects**				
Diptera	*Drosophila melanogaster*	6	Bioinformatics + MS	[1, 2]
	DTK-1		APTSSFIGMRa	
	DTK-2		APLAFVGLRa	
	DTK-3		APTGFTGMRa	
	DTK-4		APVNSFVGMRa	
	DTK-5		APNGFLGMRa	
	DTK-6[1]		pQRFADFNSKFVAVRa	
	Calliphora vomitoria	≥2	Chemical isolation	[3]
	CavTK-I		APTAFYGVRa	
	CavTK-II		GLGNNAFVGVRa	
	Culex salinarius	≥3	Chemical isolation	[4]
	CTK-I		APSGFMGMRa	
	CTK-II		APYGFTGMRa	
	CTK-III		APSGFFGMRa	
	Aedes aegypti	5	Bioinformatics + MS	[5]
	TKRP-1 and 4		APSGFLGLRa	
	TKRP-2		VPSGFTGMRa	
	TKRP-3		APSGFLGMRa	
	TKRP-5		VPNGFLGVRa	
Lepidoptera	*Bombyx mori*	5	Bioinformatics	[6]
	TK-1		IPQGFLGMRa	
	TK-2		APLGFTGVRa	
	TK-3		AANMHQFYGVRa	
	TK-4		PYDLSIRGKFIGVRa	
	TK-5		GQMGFFGMRa	
Coleoptera	*Tribolium castaneum*	8	Bioinformatics + MS	[7]
	TK-1		APSGFTGVRa	
	TK-2 x3		APSGFMGMRa	
	TK-3		APMGFMGMRa	
	TK-4		APSGFFGMRa	
	TK-5		MPRQAGFFGMRa	
	TK-6		YPYQFRGKFVGVRa	
Hymenoptera	*Apis mellifera*	7	Cloning + MS	[8-10]
	AmTRP1		APTGHQEMQa	
	AmTRP2		ALMGFQGVRa	
	AmTRP3		APMGFQGMRa	
	AmTRP4		APMGFYGTRa	
	AmTRP5		ARMGFHGMRa	
	AmTRP6		SPFRYLGARa	
	AmTRP7		NPRWEFRGKFVGVRa	

2

Hymenoptera	*Nasonia vitripennis*	9	Bioinformatics + MS	[11, 12]
	TK-1		ASMRGFQGMRa	
	TK-2 x3		APMGFQGMRa	
	TK-3		AMMGGFQGMRa	
	TK-4		ALLGFHGMRa	
	TK-5		PMMMGFHGMRa	
	TK-6[1]		SPYRFFGTRa	
	TK-7[1]		NPRWEMRGKFVGVRa	
Hemiptera	*Rhodnius prolixus*	7	Bioinformatics + MS	[13, 14]
	Rhopr-TRP1		SGPGFMGVRa	
	Rhopr-TRP2		TSMGFQGVRa	
	Rhopr-TRP3		APASGFFGMRa	
	Rhopr-TRP4		TPSDGFMGMRa	
	Rhopr-TRP5		APACVGFQGMRa	
	Rhopr-TRP6		GPSSSAFFGMRa	
	Rhopr-TRP7		SPATMGFAGVRa	
	Rhopr-TRP8		pQERRAMGFVGMRa	
Hemiptera	*Cimex lectularius*	8	Bioinformatics + MS	[15]
	TKRP-1		pQERRQLGFLGVRa	
	TKRP-2		APTLGFQGLRa	
	TKRP-3		APPMGFQGVRa	
	TKRP-4		GPTMGFVGMRa	
	TKRP-5 x2		APASGFFGMRa	
	TKRP-6		GPSNAGFFGMRa	
	TKRP-7		GPSGFLGLRa	
	TKRP-PP1 + TKRP-2		*DGTDEEFKR*APTLGFQGLRa	
Orthoptera	*Locusta migratoria*[2]	9	Chemical isolation + bioinf.	[16-18]
	Lom-TK-I		GPSGFYGVRa	
	Lom-TK-II		APLSGFYGVRa	
	Lom-TK-III		APQAGFYGVRa	
	Lom-TK-IV x 2		APSLGFHGVRa	
	Lom-TK-V		APMRGFQSVRa	
	Lom-TK-VI		ALKGFFGTRa	
	Lom-TK-VII		APSAGFHGVRa	
	Lom-TK-VIII		APVGFYGTRa	
Blattodea	*Leucophaea maderae*[2]	15	Chemical isolation + cloning	[19-21]
	LemTRP-1 (or LemTKRP-1)		APSGFLGVRa	
	LemTRP-2		APEESPKRAPSGFLGVRa	
	LemTRP-3		NGERAPGSKKAPSGFLGTRa	
	LemTKRP-3_{11-19}		APSGFLGTRa	
	LemTRP-4		APSGFMGMRa	
	LemTRP-5		APAMGFQGVRa	
	LemTRP-6		APAAGFFGMRa	
	LemTRP-7		VPASGFFGMRa	
	LemTRP-8		GPSMGFHGMRa	
	LemTRP-9 x 2		APSMGFQGMRa	
	LemTRP-10		GPNMGFMGMRa	
	LemTRP-11		MGFMGMRa	

3

	LemTRP-12		GPSVGFFAMRa	
	LemTRP-13		APSAGFMGMRa	
Blattodea	*Periplaneta americana*[3]	15		[21, 22]
	PaTK1		APSGFLGVRa	
	PaTK2		APEESPKRAPSGFLGVRa	
	PaTK3		NGERAPASKKAPSGFLGTRa	
	PaTK4		APSGFLGTRa	
	PaTK5		APGSGFMGMRa	
	PaTK6		APAMGFQGVRa	
	PaTK7		APASGFFGMRa	
	PaTK8		VPASGFFGMRa	
	PaTK9		GPSMGFHGMRa	
	PaTK10		APSLGFQGMRa	
	PaTK11		APNMGFMGMRa	
	PaTK12		MGFMGMRa	
	PaTK13		GPSVGFFAMR	
	PaTK14		APSAGFMGMRa	
	PaTK15		APSAGFHGMRa	
**Crustaceans**				
Decapoda	*Procambarus clarkii*	7	Cloning	[23]
	CabTRP-I x 7		APSGFLGMRa	
	Cancer borealis	≥1	Chemical isolation	[24]
	CabTRP-I		APSGFLGMRa	
	Carcinus maenas	4[4]	Bioinformatics	
	CabTRP-I x3		APSGFLGMRa	[25]
	CamTRP-2		TPSGFLGMRa	
Cladocera	*Daphnia pulex*	3	Bioinformatics + MS	[26]
	TRP1		TPNSRAFLGMRa	
	TRP2		KMHGEKFLGMRa	
	TRP3		APSSNSFMGMRa	
**Tick**				
Ixodida	*Ixodes scapularis*	4	Bioinformatics	[27-29]
	TKRP-1 x2		AFHAMRa	
	TKRP-2		GSGFFGMRa	
	TKRP-3		MSRTPGKEHPRSTFVATRa	
**Scorpion**				
Scorpiones	*Mesobuthus martensii*	≥6	Bioinformatics	[30]
	TK-1		pQSDNVGRTDVFVGTRa	
	TK-2		NHYYHPEKSRSSAELIFIEAI DKDSNIIYGEPIGFVATRa	
	TK-3 x3		SDSAQEEPIGFVATRa	
	TK-4		SDSAQEEPIGFVGARa	
**Spiders**				
Araneae	*Stegodyphus mimosarum*	≥2	Bioinformatics	[30]

4

	TK-1		LGNTHSPFVFLSSRSKGPAVN VHDPLAVLATGFMGSRa	
	TK-2		TDNNEIPVFANGFPAGRa	
**Tardigrade**				
Parachaela	*Hypsibius dujardini*	2[5]	Bioinformatics	[31, 32]
	TK-1		AKPSGFWGARa	
	TK-2		APSSSGFFGMRa	
**Annelids**				
Hirudinida	*Hirudo medicinalis*	≥1	Bioinformatics	[33]
	TK-1		GPPMGFHFVRa	
Polychaeta	*Capitella teleta*	5	Bioinformatics	
	TK-1		LSRGFYAARa	[33]
	TK-2		FSPKSFHFSRa	
	TK-3		AYPSGFTMPRa	
	TK-4		VPLGFQMVRa	
	TK-5		GLNKSSFFLARa	
Polychaeta	*Platynereis dumerilii*	4[6]	Bioinformatics + MS	[34]
	TK1-1[6]		MDSNQFFHMRa	
	TK1-2		QPPKGFHAVRa	
	TK2-1		MDSNQFFHMRa	
	TK2-2		QPPQGFHAVRa	
Echiura	*Urechis unicinctus*	7	Cloning + MS	[35]
	Uru-TK-I		LRQSQFVGARa	
	Uru-TK-II		AAGMGFFGARa	
	Uru-TK-III		AAPSGFFGARa	
	Uru-TK-IV[1]		PRAAYSGFFGARa	
	Uru-TK-IV		AAYSGFFGARa	
	Uru-TK-V		APSMGFFGARa	
	Uru-TK-VI[1]		APHMRFYGSRa	
	Uru-TK-VII		APKMGFFGARa	
**Molluscs**				
Bivalvia	*Anodonta cygnea*	≥1	Chemical isolation	[36]
	Anc-TK		pEYGFHAVRa	
Bivalvia	*Patinopecten yessoensis*	7[7]	Bioinformatics + MS	[37]
	TK-1		YGFHALRa	
	TK-2		NYGFHALRa	
Bivalvia	*Crassostrea gigas*	3		[38]
	Cragi-TK1		FGFAPMRa	
	Cragi-TK2		ARFFGLRa	
	Cragi-TK3		FRFTALRa	
Gastropoda	*Lottia gigantea*	4[8]	Bioinformatics	[39]
	TK-1 (ProTK1)		pQRTFGFVGTRa	
	TK-2 (ProTK1)		pQPHLGFHGMRa	

5

	TK-3 (ProTK2)		HPNFGFMGSRa	
	TK-4 (ProTK2)		pQPAFGFHAVRa	
Octopoda	*Octopus vulgaris*	7	Cloning	[40]
	Oct-TKRP-I		VNPYSFQGTRa	
	Oct-TKRP-II		LNANSFMGSRa	
	Oct-TKRP-III		TVSANAFLGSRa	
	Oct-TKRP-IV		SDALAFVPTRa	
	Oct-TKRP-V		MNSLSFGPPKa	
	Oct-TKRP-VI		YSPLDFIGSRa	
	Oct-TKRP-VII		ASLHNTHFIPSRa	
Sepiidae	*Sepia officinalis*	9	Bioinformatics	[41]
	TK-1		FSPYAFQGSRa	
	TK-2		AHASLGFVGSRa	
	TK-3		pQPASLGFVGSRa	
	TK-4		pQLNFIPSRa	
	TK-5		pQMSTAFVGSRa	
	TK-6		ISAEAFAPSRa	
	TK-7		LSSQAFFGSRa	
	TK-8		YSALGFMGSRa	
	TK-9		AAPFYHGFVASRa	
**Nematode**				
Rhabditida	*Caenorhabditis elegans*	7	Bioinformatics	[42]
	TK-1, 7		TPMQRSSMVRFa	
FLP-7	TK-2, 3, 4		SPMQRSSMVRFa	
	TK-5		SPMERSAMVRFa	
	TK-6		SPMDRSKMVRFa	
**Echinoderm**				
Forcipulatida	*Asterias rubens*	2		[43]
	ArTK1		QLWANQQSGLFa	
	ArTK2		GGGVPHVFQSGGIFa	
**Tunicate**				
Enterogona	*Ciona intestinalis*	2	Bioinformatics	[44]
	TK-1		HVRHFYGLMa	
	TK-2		ASFTGLMa	
**Cnidaria**				
Anthozoa	*Nematostella vectensis*[9]	10	Bioinformatics + MS	[45]
	TK-1		APPLDLSGPAYFHIRa	
	TK-2		GPPYIDLTEPSFFHIRa	
	TK-3		NPPIDLGPAYFHIRa	
	TK-4		pQPPIDLSPAAYFHIRa	
	TK-5		pQPPLDLGPAYFHIRa	
	TK-6		pQPPYLDLGEPSFFHIRa	
	TK-7		pQPPYLDLTPAYFHIRa	
	TK-8		pQPPYLDLTPSYFHIRa	
	TK-9		pQPPMIDLSEPAFFHIRa	
	TK-10		pQQPPMIDLSEPAFFHIRa	

6

Notes:

The C-terminal a in the sequences depics alpha-amidation

[1] Not confirmed by mass spectrometry

[2] Numbering not in the order of that in the gene

[3] This version after [22], in reference [21] a slightly different annotation was provided.

[4] There are 2 preprohormones for TKs. One precursor encodes 2 copies of CabTRP-I and the other precursor encodes one copy each of the two peptides. Both the precursors are partially elucidated so there could be additional peptides.

[5] At least 1 peptide each on 2 precursors

[6] Two precursors

[7] Only two are real TK

[8] Two genes encoding TK precursors, each with 2 TKs.

[9] These are unlikely to be *bona fide* TKs

References

1. Siviter, R.J., Coast, G.M., Winther, Å.M., Nachman, R.J., Taylor, C.A., Shirras, A.D., Coates, D., Isaac, R.E., and Nässel, D.R. (2000). Expression and functional characterization of a *Drosophila* neuropeptide precursor with homology to mammalian preprotachykinin A. J Biol Chem *275*, 23273-23280.
2. Winther, Å.M., Siviter, R.J., Isaac, R.E., Predel, R., and Nässel, D.R. (2003). Neuronal expression of tachykinin-related peptides and gene transcript during postembryonic development of *Drosophila*. J Comp Neurol *464*, 180-196.
3. Lundquist, C.T., Clottens, F.L., Holman, G.M., Nichols, R., Nachman, R.J., and Nassel, D.R. (1994). Callitachykinin I and II, two novel myotropic peptides isolated from the blowfly, Calliphora vomitoria, that have resemblances to tachykinins. Peptides *15*, 761-768.
4. Meola, S.M., Clottens, F.L., Holman, G.M., Nachman, R.J., Nichols, R., Schoofs, L., Wright, M.S., Olson, J.K., Hayes, T.K., and Pendleton, M.W. (1998). Isolation and immunocytochemical characterization of three tachykinin-rela ted peptides from the mosquito, Culex salinarius. Neurochem Res *23*, 189-202.
5. Predel, R., Neupert, S., Garczynski, S.F., Crim, J.W., Brown, M.R., Russell, W.K., Kahnt, J., Russell, D.H., and Nachman, R.J. (2010). Neuropeptidomics of the Mosquito Aedes aegypti. Journal of Proteome Research *9*, 2006-2015.
6. Roller, L., Yamanaka, N., Watanabe, K., Daubnerova, I., Zitnan, D., Kataoka, H., and Tanaka, Y. (2008). The unique evolution of neuropeptide genes in the silkworm *Bombyx mori*. Insect Biochem Mol Biol *38*, 1147-1157.
7. Hauser, F., Cazzamali, G., Williamson, M., Park, Y., Li, B., Tanaka, Y., Predel, R., Neupert, S., Schachtner, J., Verleyen, P., et al. (2008). A genome-wide inventory of neurohormone GPCRs in the red flour beetle *Tribolium castaneum*. Front Neuroendocrinol *29*, 142-165.
8. Takeuchi, H., Yasuda, A., Yasuda-Kamatani, Y., Sawata, M., Matsuo, Y., Kato, A., Tsujimoto, A., Nakajima, T., and Kubo, T. (2004). Prepro-tachykinin gene expression in the brain of the honeybee *Apis mellifera*. Cell Tissue Res *316*, 281-293.
9. Takeuchi, H., Yasuda, A., Yasuda-Kamatani, Y., Kubo, T., and Nakajima, T. (2003). Identification of a tachykinin-related neuropeptide from the honeybee brain using direct MALDI-TOF MS and its gene expression in worker, queen and drone heads. Insect Mol Biol *12*, 291-298.

7

10. Boerjan, B., Cardoen, D., Bogaerts, A., Landuyt, B., Schoofs, L., and Verleyen, P. (2010). Mass spectrometric profiling of (neuro)-peptides in the worker honeybee, Apis mellifera. Neuropharmacology *58*, 248-258.
11. Chang, J., Zhao, J., and Tian, X. (2018). In silico prediction of neuropeptides in Hymenoptera parasitoid wasps. PLOS ONE *13*, e0193561.
12. Hauser, F., Neupert, S., Williamson, M., Predel, R., Tanaka, Y., and Grimmelikhuijzen, C.J.P. (2010). Genomics and Peptidomics of Neuropeptides and Protein Hormones Present in the Parasitic Wasp Nasonia vitripennis. Journal of Proteome Research *9*, 5296-5310.
13. Ons, S., Sterkel, M., Diambra, L., Urlaub, H., and Rivera-Pomar, R. (2011). Neuropeptide precursor gene discovery in the Chagas disease vector *Rhodnius prolixus*. Insect Mol Biol *20*, 29-44.
14. Ons, S., Richter, F., Urlaub, H., and Pomar, R.R. (2009). The neuropeptidome of Rhodnius prolixus brain. PROTEOMICS *9*, 788-792.
15. Predel, R., Neupert, S., Derst, C., Reinhardt, K., and Wegener, C. (2018). Neuropeptidomics of the Bed Bug Cimex lectularius. Journal of Proteome Research *17*, 440-454.
16. Schoofs, L., Holman, G.M., Hayes, T.K., Nachman, R.J., and De Loof, A. (1990). Locustatachykinin I and II, two novel insect neuropeptides with homology to peptides of the vertebrate tachykinin family. FEBS Lett *261*, 397-401.
17. Hou, L., Jiang, F., Yang, P.C., Wang, X.H., and Kang, L. (2015). Molecular characterization and expression profiles of neuropeptide precursors in the migratory locust. Insect Biochem Molec *63*, 63-71.
18. Schoofs, L., Holman, G.M., Hayes, T.K., Kochansky, J.P., Nachman, R.J., and De Loof, A. (1990). Locustatachykinin III and IV: two additional insect neuropeptides with homology to peptides of the vertebrate tachykinin family. Regul Pept *31*, 199-212.
19. Muren, J.E., and Nässel, D.R. (1997). Seven tachykinin-related peptides isolated from the brain of the Madeira cockroach: evidence for tissue-specific expression of isoforms. Peptides *18*, 7-15.
20. Muren, J.E., and Nässel, D.R. (1996). Isolation of five tachykinin-related peptides from the midgut of the cockroach *Leucophaea maderae*: existence of N-terminally extended isoforms. Regul Pept *65*, 185-196.
21. Predel, R., Neupert, S., Roth, S., Derst, C., and Nässel, D.R. (2005). Tachykinin-related peptide precursors in two cockroach species. The FEBS journal *272*, 3365-3375.
22. Jung, J.W., Kim, J.-H., Pfeiffer, R., Ahn, Y.-J., Page, T.L., and Kwon, H.W. (2013). Neuromodulation of Olfactory Sensitivity in the Peripheral Olfactory Organs of the American Cockroach, Periplaneta americana. PLOS ONE *8*, e81361.
23. Yasuda-Kamatani, Y., and Yasuda, A. (2004). APSGFLGMRamide is a unique tachykinin-related peptide in crustaceans. European Journal of Biochemistry *271*, 1546-1556.
24. Christie, A.E., Lundquist, C.T., Nässel, D.R., and Nusbaum, M.P. (1997). Two novel tachykinin-related peptides from the nervous system of the crab *Cancer borealis*. J Exp Biol *200*, 2279-2294.
25. Christie, A.E. (2016). Expansion of the neuropeptidome of the globally invasive marine crab Carcinus maenas. Gen Comp Endocr *235*, 150-169.
26. Dircksen, H., Neupert, S., Predel, R., Verleyen, P., Huybrechts, J., Strauss, J., Hauser, F., Stafflinger, E., Schneider, M., Pauwels, K., et al. (2011). Genomics, transcriptomics, and peptidomics of daphnia pulex neuropeptides and protein hormones. J Proteome Res *10*, 4478-4504.
27. Christie, A.E. (2008). Neuropeptide discovery in Ixodoidea: an in silico investigation using publicly accessible expressed sequence tags. Gen Comp Endocrinol *157*, 174-185.

28. Neupert, S., Russell, W.K., Predel, R., Russell, D.H., Strey, O.F., Teel, P.D., and Nachman, R.J. (2009). The neuropeptidomics of Ixodes scapularis synganglion. Journal of Proteomics *72*, 1040-1045.
29. Gulia-Nuss, M., Nuss, A.B., Meyer, J.M., Sonenshine, D.E., Roe, R.M., Waterhouse, R.M., Sattelle, D.B., de la Fuente, J., Ribeiro, J.M., Megy, K., et al. (2016). Genomic insights into the Ixodes scapularis tick vector of Lyme disease. Nature communications *7*, 10507.
30. Veenstra, J.A. (2016). Neuropeptide evolution: Chelicerate neurohormone and neuropeptide genes may reflect one or more whole genome duplications. Gen Comp Endocrinol *229*, 41-55.
31. Christie, A.E., Nolan, D.H., Garcia, Z.A., McCoole, M.D., Harmon, S.M., Congdon-Jones, B., Ohno, P., Hartline, N., Congdon, C.B., Baer, K.N., et al. (2011). Bioinformatic prediction of arthropod/nematode-like peptides in non-arthropod, non-nematode members of the Ecdysozoa. Gen Comp Endocr *170*, 480-486.
32. Koziol, U. (2018). Precursors of neuropeptides and peptide hormones in the genomes of tardigrades. Gen Comp Endocr *267*, 116-127.
33. Veenstra, J.A. (2011). Neuropeptide evolution: Neurohormones and neuropeptides predicted from the genomes of Capitella teleta and Helobdella robusta. Gen Comp Endocr *171*, 160-175.
34. Conzelmann, M., Williams, E.A., Krug, K., Franz-Wachtel, M., Macek, B., and Jékely, G. (2013). The neuropeptide complement of the marine annelid Platynereis dumerilii. BMC Genomics *14*, 906-906.
35. Kawada, T., Masuda, K., Satake, H., Minakata, H., Muneoka, Y., and Nomoto, K. (2000). Identification of multiple urechistachykinin peptides, gene expression, pharmacological activity, and detection using mass spectrometric analyses. Peptides *21*, 1777-1783.
36. Fujisawa, J., Muneoka, Y., Takahashi, T., Takao, T., Shimonishi, Y., Kubota, I., Ikeda, T., Minakata, H., Nomoto, K., Kiss, T., et al. (1994). An invertebrate-type tachykinin isolated from the freshwater bivalve mollusc, *Anodonta cygnea*. In Peptide Chemistry 1993, Y. Okoda, ed. (Osaka: Protein Research Foundation), pp. 161–164.
37. Zhang, M., Wang, Y., Li, Y., Li, W., Li, R., Xie, X., Wang, S., Hu, X., Zhang, L., and Bao, Z. (2018). Identification and Characterization of Neuropeptides by Transcriptome and Proteome Analyses in a Bivalve Mollusc *Patinopecten yessoensis*. Frontiers in Genetics *9*, 197.
38. Dubos, M.-P., Zels, S., Schwartz, J., Pasquier, J., Schoofs, L., and Favrel, P. (2018). Characterization of a tachykinin signalling system in the bivalve mollusc *Crassostrea gigas*. Gen Comp Endocr *266*, 110-118.
39. Veenstra, J.A. (2010). Neurohormones and neuropeptides encoded by the genome of *Lottia gigantea*, with reference to other mollusks and insects. Gen Comp Endocrinol *167*, 86-103.
40. Kanda, A., Takuwa-Kuroda, K., Aoyama, M., and Satake, H. (2007). A novel tachykinin-related peptide receptor of *Octopus vulgaris* – evolutionary aspects of invertebrate tachykinin and tachykinin-related peptide. The FEBS Journal *274*, 2229-2239.
41. Zatylny-Gaudin, C., Cornet, V., Leduc, A., Zanuttini, B., Corre, E., Le Corguillé, G., Bernay, B., Garderes, J., Kraut, A., Couté, Y., et al. (2016). Neuropeptidome of the Cephalopod Sepia officinalis: Identification, Tissue Mapping, and Expression Pattern of Neuropeptides and Neurohormones during Egg Laying. Journal of Proteome Research *15*, 48-67.
42. Palamiuc, L., Noble, T., Witham, E., Ratanpal, H., Vaughan, M., and Srinivasan, S. (2017). A tachykinin-like neuroendocrine signalling axis couples central serotonin action and nutrient sensing with peripheral lipid metabolism. Nature communications *8*, 14237.

43. Semmens, D.C., Mirabeau, O., Moghul, I., Pancholi, M.R., Wurm, Y., and Elphick, M.R. (2016). Transcriptomic identification of starfish neuropeptide precursors yields new insights into neuropeptide evolution. Open Biol *6*, 150224.
44. Kawada, T., Sekiguchi, T., Sakai, T., Aoyama, M., and Satake, H. (2010). Neuropeptides, Hormone Peptides, and Their Receptors in *Ciona intestinalis*: An Update. Zoological Science *27*, 134-153, 120.
45. Hayakawa, E., Watanabe, H., Menschaert, G., Holstein, T.W., Baggerman, G., and Schoofs, L. (2019). A combined strategy of neuropeptide predictions and tandem mass spectrometry identifies evolutionarily conserved ancient neuropeptides in the sea anemone *Nematostella vectensis*. bioRxiv, 593384.

Supplementary Table 2. TKs identified in salivary and venom glands of invertebrates.

Species/peptide	Production site	Sequence	Reference
Aedes aegypti	Salivary gland		[1, 2]
Sialokinin I		NTGDKFYGLMa	
Sialokinin II		DTGDKFYGLMa	
Octopus vulgaris	Salivary gland		[3]
Oct-TK-I		KPPSSSEFIGLMa	
Oct-TK-II		KPPSSSEFVGLMa	
Eledone moschata	Salivary gland		[4]
Eledoisin		pEPSKDAFIGLMa	
Ampulex compressa	Venom gland		[5]
AcVTk1 x5		APMGFQGMRa	
AcVTk2		ALMGFQGMRa	
AcVTk3 x2		AVMGFQGMRa	
AcVTk4[1]		*DTDKR*GPMGFQGMRa	

Notes:

The C-terminal a in the sequences depics alpha-amidation

[1] Residues shown in italics may be cleaved off

References

1. Champagne, D.E., and Ribeiro, J.M. (1994). Sialokinin I and II: vasodilatory tachykinins from the yellow fever mosquito Aedes aegypti. Proceedings of the National Academy of Sciences *91*, 138.
2. Beerntsen, B.T., Champagne, D.E., Coleman, J.L., Campos, Y.A., and James, A.A. (1999). Characterization of the Sialokinin I gene encoding the salivary vasodilator of the yellow fever mosquito, Aedes aegypti. Insect Mol Biol *8*, 459-467.
3. Kanda, A., Iwakoshi-Ukena, E., Takuwa-Kuroda, K., and Minakata, H. (2003). Isolation and characterization of novel tachykinins from the posterior salivary gland of the common octopus Octopus vulgaris. Peptides *24*, 35-43.
4. Erspamer, V., and Anastasi, A. (1962). Structure and Pharmacological Actions of Eledoisin, Active Endecapeptide of Posterior Salivary Glands of Eledone. Experientia *18*, 58-&.
5. Arvidson, R., Kaiser, M., Lee, S.S., Urenda, J.-P., Dail, C., Mohammed, H., Nolan, C., Pan, S., Stajich, J.E., Libersat, F., et al. (2019). Parasitoid Jewel Wasp Mounts Multipronged Neurochemical Attack to Hijack a Host Brain. Molecular & Cellular Proteomics *18*, 99-114.

Supplementary Table 3. Representative frog TKs compared to human and fish TKs

Species[1]	Tissue[2]	Type	Amino acid sequence	References
Homo sapiens		SP	RPKPQQFFGLMa	[1, 2]
Xenopus tropicalis		SP-like	KPRPDQFYGLMa	NCBI search
Theroderma kwangsiensis	skin	SP-like	KPSPDRFYGLMa	[3]
Kassina senegalensis	skin	SP-like	DVPKSDQFVGLMa	[4]
Kassina senegalensis	skin	SP-like	DTPKSDQFIGLMa	[4]
Homo sapiens		NKA	HKTDSFVGLMa	[1, 2]
Xenopus tropicalis		NKA-like	YKSGSFFGLMa	NCBI search
Homo sapiens		NKB	DMHDFVGLMa	[1, 2]
Rana ridibunda	brain	NKB-like	DMHDFFVGLMa	[5]
Xenopus tropicalis		NKB-like	EMNDFFVGLMa	NCBI search
Danio rerio		NKF	YNDIDYDSFVGLMa	[6]
Xenopus tropicalis		NKF-like	FYDDDSFVGLMa	NCBI search
Physalaemus fuscumaculatus	skin		pEADPNKFYGLMa	[7]
Oddorana grahami	skin		DDTEDLANKFIGLMa	[8]
Oddorana grahami	skin		DDASDRAKKFIGLMa	[8]
Rana chensinensis	skin		DDTSDRSNGFIGLMa	[9]

Notes:
The C-terminal a in the sequences depics alpha-amidation
[1] Frog species shown with blue background
[2] Tissue in frogs

References

1. Satake, H., Aoyama, M., Sekiguchi, T., and Kawada, T. (2013). Insight into Molecular and Functional Diversity of Tachykinins and their Receptors. Protein Peptide Lett *20*, 615-627.
2. Steinhoff, M.S., von Mentzer, B., Geppetti, P., Pothoulakis, C., and Bunnett, N.W. (2014). Tachykinins and Their Receptors: Contributions to Physiological Control and the Mechanisms of Disease. Physiological Reviews *94*, 265-301.
3. Zhang, H., Wei, L., Zou, C., Bai, J., Song, Y., and Liu, H. (2013). Purification and characterization of a tachykinin-like peptide from skin secretions of the tree frog, *Theloderma kwangsiensis*. Zoolog Sci *30*, 529-533.
4. Anastasi, A., Montecucchi, P., Erspamer, V., and Visser, J. (1977). Amino acid composition and sequence of kassinin, a tachykinin dodecapeptide from the skin of the African frog *Kassina senegalensis*. Experientia *33*, 857-858.
5. O'Harte, F., Burcher, E., Lovas, S., Smith, D.D., Vaudry, H., and Conlon, J.M. (1991). Ranakinin: A Novel NK1 Tachykinin Receptor Agonist Isolated with Neurokinin B from the Brain of the Frog Rana ridibunda. Journal of Neurochemistry *57*, 2086-2091.
6. Biran, J., Palevitch, O., Ben-Dor, S., and Levavi-Sivan, B. (2012). Neurokinin Bs and neurokinin B receptors in zebrafish-potential role in controlling fish reproduction. Proceedings of the National Academy of Sciences *109*, 10269.
7. Bertaccini, G., Cei, J.M., and Erspamer, V. (1965). OCCURRENCE OF PHYSALAEMIN IN EXTRACTS OF THE SKIN OF PHYSALAEMUS FUSCUMACULATUS AND ITS PHARMACOLOGICAL ACTIONS ON EXTRAVASCULAR SMOOTH MUSCLE. Brit J Pharm Chemoth *25*, 363-379.
8. Li, J., Liu, T., Xu, X., Wang, X., Wu, M., Yang, H., and Lai, R. (2006). Amphibian tachykinin precursor. Biochem Bioph Res Co *350*, 983-986.
9. Wu, Y., Renjie, L., Jie, M., Mei, Z., Lei, W., Timothy, I.M., Jiqun, C., Tianbao, C., and Chris, S. (2013). Ranachensinin: A Novel Aliphatic Tachykinin from the Skin Secretion of the Chinese Brown Frog, Rana chensinensis. Protein & Peptide Letters *20*, 1217-1224.

Supplementary Material Table 4. Colocalization of TK or natalisin with neuropeptides and neurotransmitters in neurons and endocrine cells of *Drosophila* and *Locusta migratoria*[1].

Tissue	Cell type	Substances[2]	Reference
Drosophila melanogaster			
Brain	ITPn (lateral neurosecretory cells)	ITP, sNPF, TK	[1]
Brain	LN (local neurons; antennal lobe)	TK, GABA[4]	[2]
Brain	LN (local neurons; antennal lobe)	TK, MIP	[3]
Brain	LN (local neurons; antennal lobe)	TK, Ast-A	[3]
Midgut	Endocrine cells	TK, NPF	[4]
Midgut	Endocrine cells, posterior	TK, DH31	[4]
Brain	ICLI (large natalisin interneurons)	NTL, AstA, MIP	[5]
Locusta migratoria			
Brain	Central complex	TK, LK	[6]
Brain	Central complex	TK, octopamine	[6]
Brain	Central complex	TK, GABA	[6]
Legs	Sensory neurons	TK, AT, FMRFamide, ACh	[7]
Midgut	Endocrine cells in midgut ampullae	TK, DH44, FMRFamide	[8]

Notes

[1] Using antisera and/or Gal4 lines

[2] Substances (in order of appearance in Table)
ITP, ion transport peptide
sNPF, short neuropeptide F
TK, tachykinin
GABA, gamma aminobutyric acid
MIP, myoinhibitory peptide
Ast-A, allatostatin A
NPF, neuropeptide F
DH31, diuretic hormone 31
NTL, natalisin
LK, leucokinin
AT, allatotropin
ACh, acetylcholine
DH44, diuretic hormone 44

References

1. Kahsai, L., Kapan, N., Dircksen, H., Winther, Å.M., and Nässel, D.R. (2010). Metabolic stress responses in *Drosophila* are modulated by brain neurosecretory cells that produce multiple neuropeptides. PLoS ONE *5*, e11480.
2. Ignell, R., Root, C.M., Birse, R.T., Wang, J.W., Nässel, D.R., and Winther, Å.M. (2009). Presynaptic peptidergic modulation of olfactory receptor neurons in *Drosophila*. Proc Natl Acad Sci U S A *106*, 13070-13075.
3. Carlsson, M.A., Diesner, M., Schachtner, J., and Nässel, D.R. (2010). Multiple neuropeptides in the *Drosophila* antennal lobe suggest complex modulatory circuits. J Comp Neurol *518*, 3359-3380.
4. Veenstra, J.A., Agricola, H.J., and Sellami, A. (2008). Regulatory peptides in fruit fly midgut. Cell Tissue Res *334*, 499-516.
5. Diesner, M., Predel, R., and Neupert, S. (2018). Neuropeptide Mapping of Dimmed Cells of Adult Drosophila Brain. J Am Soc Mass Spectrom *29*, 890-902.
6. Vitzthum, H., and Homberg, U. (1998). Immunocytochemical demonstration of locustatachykinin-related peptides in the central complex of the locust brain. J Comp Neurol *390*, 455-469.
7. Persson, M.G., and Nässel, D.R. (1999). Neuropeptides in insect sensory neurones: tachykinin-, FMRFamide- and allatotropin-related peptides in terminals of locust thoracic sensory afferents. Brain Res *816*, 131-141.
8. Johard, H.A., Coast, G.M., Mordue, W., and Nässel, D.R. (2003). Diuretic action of the peptide locustatachykinin I: cellular localisation and effects on fluid secretion in Malpighian tubules of locusts. Peptides *24*, 1571-1579.

1

Supplementary Table 5. Natalisin sequences from select arthropods

Order	Species	Copies	Sequence[1] / method	Reference
**Insects**				
Diptera	*Drosophila melanogaster*	5	Bioinformatics	[1]
	1		EKLFDGYQFGEDMSKENDPFIPPRa	
	2		HSGSLDLDALMNRYEPFVPNRa	
	3		DKVKDLFKYDDLFYPHRa	
	4		HRNLFQVDDPFFATRa	
	5		LQLRDLYNADDPFVPNRa	
	Aedes aegypti	8	Bioinformatics	[1]
	1		EILTHPSGGDIPLLDTQQHLRa	
	2		LPFYVDEPRYVVIa	
	3		PSLFQSNGGFSFIPQRa	
	4		ELSIQQMLQGSDYFVPNRa	
	5		IKFDDILGSDELFIPNRa	
	6		ELFDLFPAMTRa	
	7		ESDSGGELFYPTRa	
	8		NILENLAQSQDTFFSSRa	
	Anopheles gambiae	6	Bioinformatics	[1]
	1		ESASPSEAETGa	
	2		GLPSPLVFMPARa	
	3		GDYFVPNRa	
	4		APTNGELIKKa	
	5		FDVLLGGSPDEYFFPNRa	
	6		NLLENLANEHKDTFFSSRa	
Lepidoptera	*Bombyx mori*	11	Bioinformatics	[1]
	1		IHNEPPFWAIRa	
	2		IGLWNEPDLKHPANFWANRa	
	3		DLRQENDPFWGNRa	
	4		EEAFWSSKa	
	5		TEENPFWANRa	
	6		DSNTDVDPFWGSRa	
	7		SPGAGLNFWLNRa	
	8		SSAEDDPFYISRa	
	9		YYLKYNFGRPa	
	10		SVRNDPYYIARa	
	11		LAAQLQNDPYFASRa	
Coleoptera	*Tribolium castaneum*	2	Bioinformatics	[1]
	1		ASGQEEFGPFWANRa	
	2		DDNDINDNEPFYVTRa	
Hemiptera	*Rhodnius prolixus*	4	Bioinformatics	[1]
	1		AVLGSSEAEPGFWPTRa	
	2		GDSSSTEEVQPPFWAHRa	
	3		DTMEQDPFWVSRa	
	4		ITVTNSFAGEMRGLWSLa	

2

Crustaceans				
Cladocera	*Daphnia pulex*	5	Bioinformatics	[1]
	1		GNTDQDMFWAARa	
	2		DGTFWAARa	
	3		YAADGGDGVPFWATRa	
	4		GDLEIPFWAARa	
	5		PASQAAEPFWAARa	
**Arachnids**				
Trombidiformes	*Tetranychus urticae*	2	Bioinformatics	[1]
	1		SAFNGMRa	
	2		ARPFAAMLa	
Parasitiformes	*Varroa destructor*	2	Bioinformatics	[2]
	1		SIGGPAPGFVGARa	
	2		GGVPGFVGARa	

Note:

[1] The C-terminal a in the sequences depics alpha-amidation

References

1. Jiang, H., Lkhagva, A., Daubnerova, I., Chae, H.S., Simo, L., Jung, S.H., Yoon, Y.K., Lee, N.R., Seong, J.Y., Zitnan, D., et al. (2013). Natalisin, a tachykinin-like signaling system, regulates sexual activity and fecundity in insects. Proc Natl Acad Sci U S A *110*, E3526-3534.
2. Jiang, H., Kim, D., Dobesh, S., Evans, J.D., Nachman, R.J., Kaczmarek, K., Zabrocki, J., and Park, Y. (2016). Ligand selectivity in tachykinin and natalisin neuropeptidergic systems of the honey bee parasitic mite Varroa destructor. Sci Rep *6*, 19547.
